# Tailoring HIPEC with Patient-Derived Organoids in Colorectal Peritoneal Metastases: Results from the First Stage of the Prospective Phase II OrganoHIPEC Clinical Trial (Clinicaltrials.gov NCT06057298)

**DOI:** 10.3390/cancers18162722

**Published:** 2026-08-21

**Authors:** Dario Baratti, Luca Varinelli, Marcello Guaglio, Shigeki Kusamura, Tommaso Cavalleri, Davide Battistessa, Giovanna Sabella, Gaia Colletti, Manuela Gariboldi, Marcello Deraco

**Affiliations:** 1 Peritoneal Malignancy Program, Department of Surgery, Fondazione IRCCS Istituto Nazionale dei Tumori, via Venezian 1, 20133 Milan, Italy; marcello.guaglio@istitutotumori.mi.it (M.G.); shigeki.kusamura@istitutotumori.mi.it (S.K.); tommaso.cavalleri@istitutotumori.mi.it (T.C.); gaia.colletti@istitutotumori.mi.it (G.C.);; 2 Molecular Epigenomics Unit, Department of Experimental Oncology, Fondazione IRCCS Istituto Nazionale dei Tumori, via G. Venezian 1, 20133 Milan, Italy; luca.varinelli@istitutotumori.mi.it (L.V.); davide.battistessa@istitutotumori.mi.it (D.B.); manuela.gariboldi@istitutotumori.mi.it (M.G.); 3 Department of Pathology and Laboratory Medicine, Fondazione IRCCS Istituto Nazionale dei Tumori, via Venezian 1, 20133 Milan, Italy; giovanna.sabella@istitutotumori.mi.it

**Keywords:** colorectal cancer, peritoneal metastases, patient-derived organoids, cytoreductive surgery, hyperthermic intraperitoneal chemotherapy, HIPEC, tailored therapy

## Abstract

**Background/Objectives**: OrganoHIPEC is a phase-II, two-stage, open-label clinical trial that investigates if cytoreductive surgery (CRS) and patient-tailored HIPEC, based on a preclinical platform using patient-derived organoids, can improve disease control in peritoneal metastases from colorectal cancer (CRC-PM). **Methods**: Adults with limited CRC-PM and no distant metastases were included. CRC-PM were sampled for organoid development during diagnostic laparoscopy. These organoids were used in an in vitro HIPEC model to test various drugs suitable for intraperitoneal administration. After 3–6 months of systemic chemotherapy, patients without progression underwent CRS/HIPEC with personalized regimens based on organoid drug response. To detect an increase in 12-month peritoneal disease-free survival from 40% to 60%, 24 patients are needed. According to the two-stage design, if <7 of 10 patients in Stage-1 remain PM-free at 12 months, the trial is terminated. **Results**: Forty-seven patients were enrolled. Among 31 patients with available organoid data, the most active drugs were mitomycin-C (*n* = 14), cisplatin/mitomycin-C (*n* = 12), and low-dose (120 min) oxaliplatin (*n* = 4). No patient was sensitive to high-dose oxaliplatin (30 min) and cisplatin/doxorubicin. Ten patients had a potential follow-up >12 months. Peritoneal relapse occurred at 8 months in two patients, and one died of liver metastases at 7 months. Seven patients remained PM-free for >12 months (median 16.4, range 12.6–28.4). **Conclusions**: A comprehensive precision approach using patient-derived organoids to guide personalized HIPEC is feasible and shows promising early results. High-dose oxaliplatin is poorly active. As 7/10 patients achieved the endpoint of 12-month PM-free survival, Stage-1 was successfully completed. The trial is proceeding to Stage-2.

## Simple Summary

Peritoneal metastases are the second most common metastatic site in colorectal cancer. Patients with peritoneal metastases experience significant cancer-related morbidity and a dismal prognosis. Although cytoreductive surgery and hyperthermic intraperitoneal chemotherapy (CRS/HIPEC) have been shown to improve survival in selected patients, peritoneal relapses remain common. A recent randomized trial has further questioned the role of oxaliplatin-based HIPEC, but there is currently no consensus on the drug of choice for HIPEC. We hypothesize that inefficiency in eliminating microscopic residual disease in a subset of patients explains the high relapse rates after HIPEC with routinely administered drug schedules. Therefore, we designed a comprehensive approach involving peritoneal metastases-derived organoids and an in vitro HIPEC model to select the most active drug(s) at the individual patient level, to increase the efficacy of CRS and patient-tailored HIPEC in controlling peritoneal disease. We present the results of the successful first stage of our phase-2 clinical trial.

## 1. Introduction

Colorectal cancer (CRC) is the third most common cancer worldwide [1]. Peritoneal metastases (PM) remain a major cause of cancer-related mortality, as modern highly effective systemic chemotherapy (s-CT) combined with targeted agents still results in lower survival benefit compared to extra-peritoneal metastases [2]. Nevertheless, survival improvements have been reported in selected patients treated by aggressive cytoreductive surgery combined with hyperthermic intraperitoneal chemotherapy (CRS-HIPEC) to control the microscopic residual disease [3,4].

The curative-intent approach involving CRS/HIPEC has been shown to increase survival over palliative s-CT in a landmark randomized trial [5]. However, the role of HIPEC has been recently questioned following the randomized Prodige-7 trial, which failed to demonstrate a survival benefit from adding oxaliplatin-based HIPEC to complete CRS and six months of s-CT [6].

The limited efficacy of intraperitoneal oxaliplatin is a possible explanation for the negative results of the Prodige-7 trial [7], but there is currently no consensus about the optimal drug regimen for HIPEC in CRC-PM. Mitomycin-C, alone or combined with cisplatin, is widely used, but retrospective comparisons with oxaliplatin have yielded conflicting results [8,9,10]. The combination of oxaliplatin and irinotecan has not shown an additional benefit over oxaliplatin alone [11]. Furthermore, we are unable to predict how effective a given drug will be at the individual patient level.

Recent advances in tridimensional cell culture, such as patient-derived organoids (PDO), have provided more specific human cancer models that can be used to predict therapeutic responses in individual patients and develop new therapeutic strategies [12,13]. Our group, along with others, has established CRC-PM-derived organoids that maintain the morphological, immunohistochemical, and mutational features of the tumor of origin [13,14]. Furthermore, other groups, as well as our group, have developed in vitro HIPEC models to assess drug activity under conditions closely resembling the clinical setting, demonstrating that sensitivity to commonly used chemotherapeutics varies widely among PDO [14,15,16].

We hypothesized that inefficiency in eliminating microscopic residual disease in a subset of patients explains the high relapse rates after HIPEC with routinely administered drugs. We also hypothesized that PDO can be used as representative in vitro models of CRC-PM to identify the most active HIPEC treatment on an individual patient basis. Herein, we present the preliminary results of a clinical trial designed to investigate if CRS and patient-tailored (PT)-HIPEC, based on a preclinical platform using patient-derived CRC-PM organoids, can improve disease control.

## 2. Materials and Methods

The OrganoHIPEC trial is an investigator-initiated, phase II, open-label, single-arm, two-stage clinical study. The study protocol is presented in Supplement S1.

### 2.1. Study Objectives and Endpoints

The primary objectives are to assess feasibility, efficacy, and safety of PT-HIPEC in the treatment of CRC-PM. The primary endpoint is peritoneal metastasis-free survival, defined as the time interval from the date of CRS/PT-HIPEC to the date of recurrent PM diagnosis. Feasibility is assessed by the proportion of enrolled patients who undergo CRS/PT-HIPEC. A subset of patients is expected to be unable to complete the study protocol for reasons including, but not limited to, patient withdrawal, failure to retrieve adequate tumor samples, establish PDO, and perform in vitro drug sensitivity tests, or disease progression during preoperative s-CT. Toxicity is scored using the Common Terminology Criteria for Adverse Events (CTCAE) version 4.0 [17].

### 2.2. Study Population

A complete list of eligibility criteria is provided in the study protocol (see Supplement S1). Briefly, patients with a pathological diagnosis of PM from colorectal adenocarcinoma of limited to moderate extent, and potentially amenable to complete surgical cytoreduction were considered for inclusion if they met the following criteria: absence of systemic metastases; age ≥ 18; performance status ≤ 2 according to the World Health Organization score; provision of informed consent.

Patients underwent an intensive preoperative work-up including physical examination, colonoscopy, contrast-enhanced thoracic, abdominal and pelvic computed tomography (CT), CEA and CA19.9 measurements. We performed additional studies, such as fluorodeoxyglucose positron emission tomography or nuclear resonance imaging, as needed. All patients were discussed at multidisciplinary meetings.

### 2.3. Interventions

**Figure 1:**
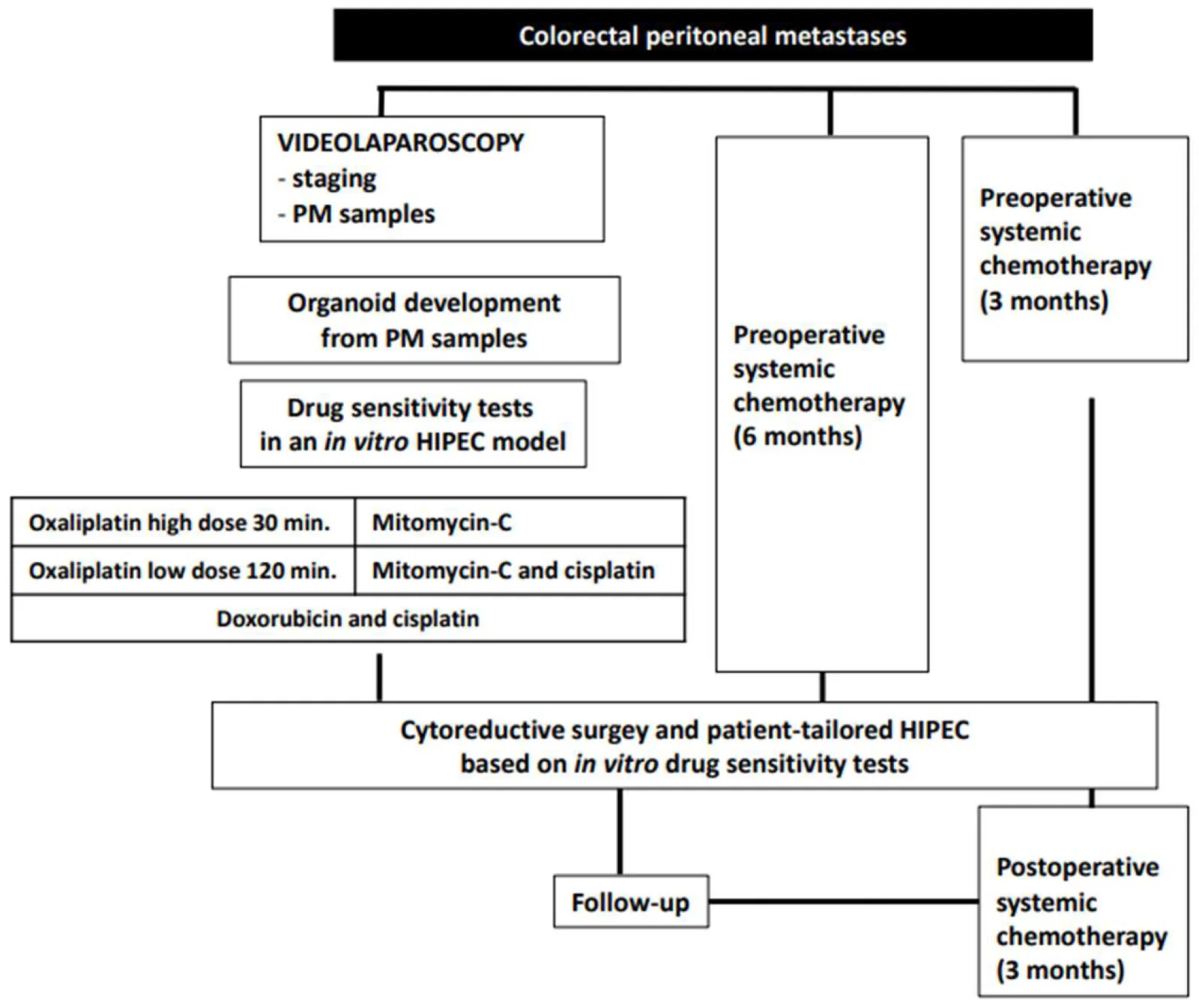
**Study flow-chart.** PM: peritoneal metastases; HIPEC: hyperthermic intraperitoneal chemotherapy.

Figure 1 illustrates the flowchart of the trial procedures.

#### 2.3.1. Human Samples Collection

Samples of the peritoneal tumor for PDO development were obtained during laparoscopic procedures performed to confirm CRC-PM, stage the disease, or palliate symptoms, such as obstruction, perforation, acute bleeding, or severe anemia. Patients undergoing open surgery were not excluded. During surgery, the abdominal cavity was thoroughly assessed, and the extent of peritoneal involvement scored using the peritoneal cancer index (PCI) [18]. Representative samples of CRC-PM ≥ 5 mm in diameter were collected from at least three different peritoneal sites, whenever technically possible and with reasonable risk.

#### 2.3.2. Organoid Development and In Vitro Drug Assays

Our methodology of TDO development and in vitro drug assays has been previously described in detail [14,16]. Briefly, surgical specimens were digested with standard enzymatic methods and cultured in Matrigel and DMEM F12 medium, under serum-free conditions and factors specific to the colonic niche [19]. To mimic HIPEC treatment, a 96-well plate was coated with Matrigel. PDOs were resuspended in 2% Matrigel/growth media and dispensed into wells. A dilution series of drugs suitable for HIPEC was added, and cell viability was assayed. Dose–response curves were fitted to the luminescent signal intensities [20], and the half-maximal inhibitory concentrations (IC50) were determined [21]. HIPEC drug schedules tested in vitro were derived from published dose-finding trials or, if phase I studies were not available, large literature series reporting safety, tolerability, and efficacy, as shown in Table 1 [22,23,24].

#### 2.3.3. Preoperative Systemic Chemotherapy

All enrolled patients received s-CT in the preoperative phase, to comply with our national guidelines [25,26], and to not remain without treatment during the time interval needed for establishing PDO and performing drug-sensitivity assays. Perioperative s-CT was administered according to current standards [27], and consisted of either four/eight 3-weekly cycles of capecitabine with oxaliplatin (XELOX), six/twelve 2-weekly cycles of 5-fluorouracil/leucovorin with oxaliplatin (FOLFOX), six/twelve 2-weekly cycles of 5-fluorouracil/leucovorin with irinotecan (FOLFIRI), or FOLFOXIRI. Bevacizumab, or anti-epidermal growth factor receptor (EGFR) agents in RAS/RAF wild-type tumors could be added. In case of unacceptable toxicity, s-CT was switched to fluoropyrimidine or discontinued. The choice between delivering s-CT exclusively in the preoperative phase, or partly in the preoperative phase and partly in the postoperative phase was left to the treating medical oncologists.

#### 2.3.4. Response Assessment

Response to s-CT was assessed after either the first four cycles of XELOX, or six cycles of FOLFOX, FOLFIRI, or FOLFOXIRI by clinical examination, CEA and CA19.9 measurement, and thoracic-abdominal CT scan. In patients with measurable peritoneal lesions, the response was graded according to RECIST 1.1 criteria [28]. Patients with response or stable disease were continued on the same treatment until eight cycles of XELOX, or twelve cycles of FOLFOX, FOLFIRI, or FOLFOXIRI, or moved to CRS/PT-HIPEC.

#### 2.3.5. Cytoreductive Surgery and Patient-Tailored HIPEC

CRS/PT-HIPEC was planned within four/six weeks after the completion of preoperative s-CT, and at least six/eight weeks after the last administration of bevacizumab. Cytoreductive surgery was aimed at removing all macroscopic tumors by peritonectomy procedures and visceral resection, as needed [29,30]. The extent of the peritoneal tumor remaining after the surgical cytoreduction was scored by the completeness of the cytoreduction (CCR) score [18].

#### 2.3.6. Patient-Tailored Hyperthermic Intra-Peritoneal Chemotherapy

HIPEC was performed according to the closed abdomen technique. Carrier solution volume was calculated based on the body surface area, at 3 L/mq, and further adjusted according to factors such as abdominal wall compliance and the type and number of organs removed during the CRS (which translate into increased empty volume). Generally, four/six L were needed. The perfusion flow was set at of 600/700 mL/min, and target temperature inside the abdomen was 42.0–42.5 °C. Drug schedules are shown in Table 1.

#### 2.3.7. Follow-Up

Follow-up visits were scheduled three-monthly during the first two years, and six-monthly thereafter. Thoracic and abdominal CT scans, CEA and CA19.9 determination, renal, hepatic and hematological function tests were performed at each visit. Colonoscopy is performed at 12, and 36 months postoperatively.

### 2.4. Statistics

Based on literature data, approximately 50–65% of patients are expected to remain free of CRC-PM one year after CRS/HIPEC [4,5,6]. In a recent collaborative Italian study, in which we participated, 12-month peritoneal disease-free survival was 60% [31]. To demonstrate an absolute increase of 20%, from 60% to 80%, with a type I error rate (alpha) = 0.1, and power (1-ß) = 0.8, a total of 24 patients are needed.

The study is conducted according to the two-stage design described by Fleming and Jung [32,33]. The first stage is intended to exclude treatments that are clearly ineffective, treating as few patients as possible. The second stage determines whether the treatment achieves a success rate above a designated threshold level, and therefore warrants further evaluation. Treatment success is defined as the absence of clinically and radiologically demonstrable CRC-PM at 12 months following CRS/PT-HIPEC. Ten patients are enrolled in the first stage of the study. If ≥7 patients are free of PM at 12 months, the study proceeds until a total of 24 patients (including the first ten patients) have been enrolled. The null hypothesis is rejected if at least 18 patients are free of PM at 12 months.

Categorical variables were described in terms of frequency and percentages, and continuous variables in terms of mean, standard error, median, and inter-quartile ranges. Differences between groups were assessed by the Student t-test, Chi-square test, or Fisher’s exact test, as appropriate. All statistical analyses were conducted by SPSS, version 20.0.0 (IBM Corporation, Armonk, NY, USA). Two-sided *p* values < 0.05 are considered significant.

## 3. Results

From May 2021 to March 2026, 47 patients were enrolled. Their clinical–pathological characteristics are shown in Table 2.

### 3.1. PM Sample Collection

PM samples were collected during minimally invasive procedures in 37 patients (78.7%), consisting of staging laparoscopy (*n* = 26), diverting ostomies (*n* = 5), bilateral adnexectomy (*n* = 4), right colectomy (*n* = 1), and anterior rectal resection (*n* = 1). Median operative time was 85 min (range 43–201). Only one patient experienced a major (grade 3–4) postoperative complication, namely colostomy prolapse.

Open surgery was performed in the remaining 10 patients (21.3%), including right colectomy (*n* = 2), bilateral adnexectomy (*n* = 2), wide excision of abdominal wall metastases (*n* = 2), colon resection with histero-adnexectomy (*n* = 2), small bowel resection (*n* = 1), Hartmann procedure with bilateral adnexectomy (*n* = 1). Major complications occurred in two patients following open surgery.

In the overall series, median PCI was 8 (range 0–30). PCI > 15 was recorded in seven patients. Twenty patients received adjuvant s-CT at the time of primary resection (42.6%). s-CT was oxaliplatin-based in 18 of them. Ten (21.3%) patients received s-CT for the treatment of PM before tumor sampling (oxaliplatin-based in nine).

### 3.2. Organoid Development and In Vitro Drug Assay

The radiological diagnosis of PM was not confirmed at laparoscopy in three patients. Organoids are still being generated for one additional patient. Drug sensitivity test results are available for 31 of the remaining 43 patients, corresponding to a success rate of 72.1%.

**Figure 2:**
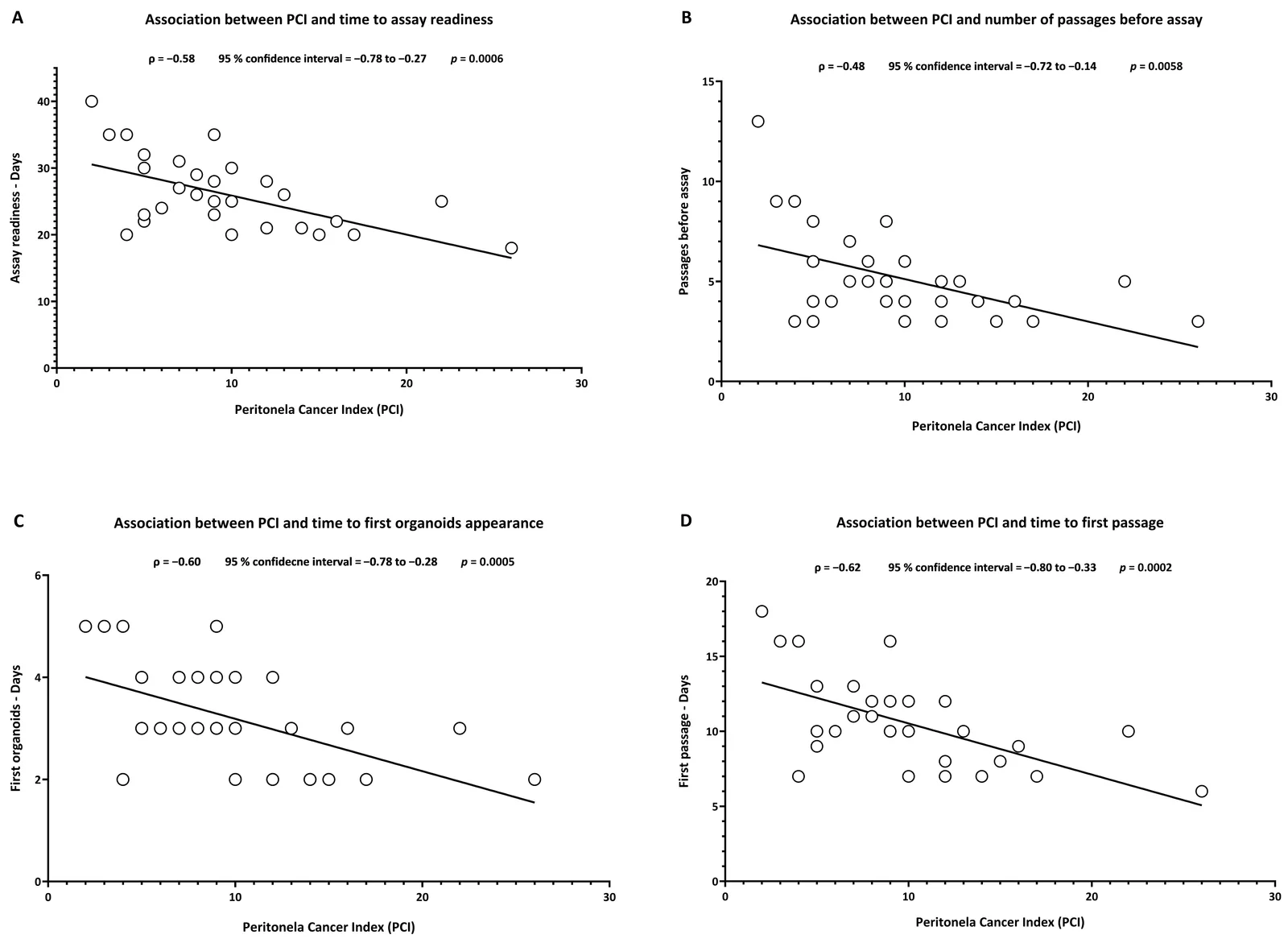
**Association between peritoneal disease burden and PDO establishment and expansion parameters.** Scatter plots showing the association between the Peritoneal Cancer Index and (**A**) time to assay readiness, (**B**) number of passages required before assay completion, (**C**) time to first organoid appearance, and (**D**) time to first passage. Each point represents one successfully established patient-derived organoid line (*n* = 31). Associations were assessed using Spearman’s rank correlation coefficient, and the corresponding ρ values, 95% confidence intervals, and two-sided *p*-values are reported. Regression lines are shown solely as visual guides and do not represent the statistical model used for the correlation analysis. All temporal parameters were calculated from the date of tissue processing. PCI, Peritoneal Cancer Index; PDO, patient-derived organoid.

Regarding PDO development, the median time to first organoid appearance was 3 days (range 2–5), the median time to first passage was 10 days (range 6–18), and the median time to assay readiness was 25 days (range 18–40). A median of four passages was required before assay completion (range, 3–13). Patient-level data are reported in Supplement S2. Higher PCI values were significantly associated with shorter time to assay readiness (Spearman’s ρ = −0.58; *p* = 0.0006). Consistent inverse associations were observed between PCI and time to first organoid appearance (ρ = −0.59; *p* = 0.0005), time to first passage (ρ = −0.62; *p* = 0.0002), and number of passages required before assay completion (ρ = −0.48; *p* = 0.0058) (See Figure 2). These results indicate that PDOs derived from patients with a greater peritoneal disease burden tended to establish and expand more rapidly ex vivo.

The most active drug or drug combination was mitomycin-C in 13 patients, cisplatin plus mitomycin-C in 12, and low-dose oxaliplatin in 4. One patient showed equal sensitivity to mitomyxcin-C alone and mitomyxcin-C plus cisplatin. One patient was resistant to all tested drugs. No organoid line was sensitive to high-dose oxaliplatin and cisplatin plus doxorubicin.

Table 3 shows the correlation between several clinical–pathological variables and both TDO development and sensitivity test results. There was a trend toward a higher likelihood of successful TDO development in patients with intestinal-type histology, mutated KRAS, and no prior s-CT, although these associations did not reach statistical significance. Analogously, no correlation was observed between the variables analyzed and the sensitivity to the different drugs. Interestingly, all four cases in which oxaliplatin was the most active drug were not previously exposed to systemic oxaliplatin.

### 3.3. Preoperative Systemic Chemotherapy and Response Assessment

Excluding three patients in whom PM were not confirmed, and one patient who died from disease progression shortly after the laparoscopy, preoperative s-CT was started in 43 patients. S-CT was oxaliplatin-based in 20 patients, irinotecan-based in 17, and fluoropyrimidine-based in two. Three patients received FOLFOXIRI, and one Trifluridine/Tipiracil plus capecitabine. Bevacizumab was administered to 31 patients, and anti-EGFR agents to 12.

Disease control (complete/partial response or stable disease) was obtained in 20 patients (55.5%). Conversely, disease progression during s-CT occurred in 16 patients (44.5%), involving the peritoneum (*n* = 11), liver (*n* = 2), lung (*n* = 1), distant nodes (*n* = 1), and both liver and bone (*n* = 1). The remaining seven patients are currently receiving s-CT.

### 3.4. Cytoreductive Surgery and PT-HIPEC

Among the 21 patients who did not experience disease progression, 13 underwent CRS and PT-HIPEC according to the organoid sensitivity tests. Four additional patients underwent CRS/HIPEC according to the protocol routinely adopted in our center (cisplatin plus mitomycin-C) because organoids could not be established. One patient is waiting for CRS/PT-HIPEC, and two refused surgery. An additional patient, in whom organoids could not be established, was shown to have a complete response at repeat laparoscopy and was not operated on.

The characteristics of the 10 patients undergoing CRS and PT-HIPEC during Stage-1 are shown in Table 4. The mean operative time was 552 min (SD 92; median 537; range 413–797). Cytoreduction was macroscopically complete in nine patients and nearly complete in one. Major complications occurred in five patients, requiring reoperation in four, with no operative death. Mean hospital stay was 22.7 days (SD 11.6; median 17; range 11–41).

### 3.5. Survival Outcomes

**Figure 3:**
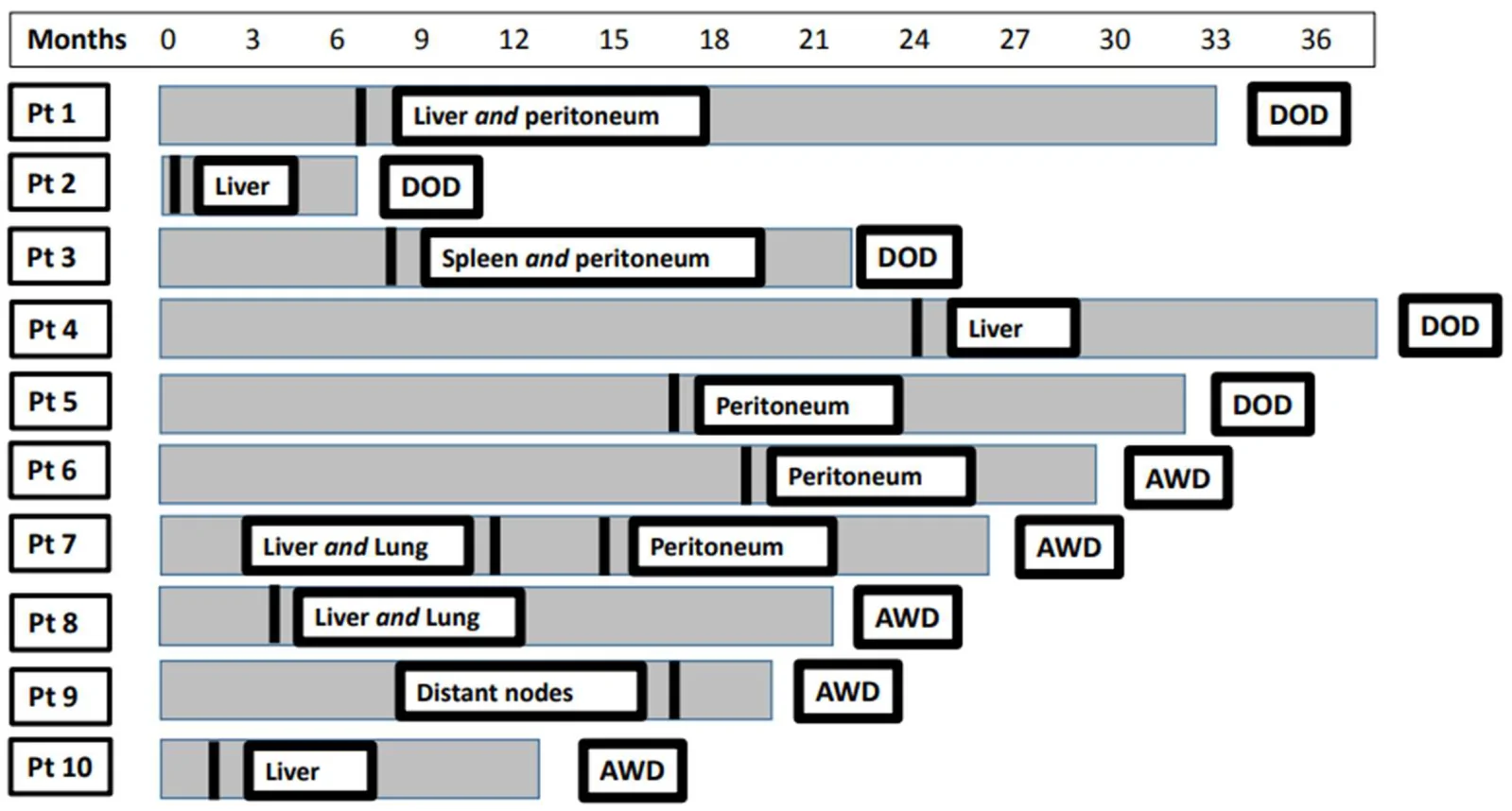
**Oncological outcomes of 10 patients undergoing cytoreductive surgery and patient-tailored hyperthermic intraperitoneal chemotherapy.** DOD: dead of the disease; AWD: alive with the disease.

To date, ten patients enrolled in the first stage of the study have reached a potential follow-up >12 months. Peritoneal recurrences occurred in five of them at 8, 8, 17, 19, and 15 months after CRS/PT-HIPEC, respectively. One patient died at 7 months of distant metastases, without evidence of peritoneal relapse. The remaining four patients remained free of PM for 22, 27, 17, and 12 months, respectively. Therefore, seven of ten patients achieved the primary endpoint of 12-month peritoneal disease-free survival (see Figure 3).

Distant metastases occurred in eight patients, involving the liver in four, both liver and lung in two, and spleen and mediastinal nodes in one case each. Three of them experienced peritoneal recurrences also. Five patients have died at a median of 32 months (range 7–37), and five are alive at a median of 21 months (range 12–30).

Three patients have undergone CRS/PT-HIPEC after the completion of the first stage. Regarding the 16 patients who experienced disease progression under s-CT, 10 have died after a median of 10 months from enrollment in the study.

## 4. Discussion

Despite recent advances in systemic chemotherapy, targeted therapy and immunotherapy, colorectal peritoneal metastases are still a major cause of cancer-related morbidity and mortality. Although the combined approach of CRS/HIPEC has improved survival, a substantial proportion of patients still experience disease recurrence after optimal multimodal treatment and ultimately die of their disease [4].

Given the inherently incomplete nature of cytoreductive surgery, the use of intraperitoneal chemotherapy is supported by a robust rationale, namely the need to target the microscopic residual disease. However, the unsuccessful Prodige-7 trial has recently questioned the value of oxaliplatin-based HIPEC [6,7]. Conversely, phase III studies in ovarian and gastric cancer, as well as in the prophylactic setting for colon cancer, have provided clinical evidence supporting HIPEC efficacy [34,35,36]. Taken together, these findings suggest that efforts should be directed toward improving HIPEC efficacy, rather than abandoning its use in CRC-PM treatment.

The present study investigates a treatment strategy combining CRS and patient-tailored HIPEC, guided by a preclinical platform based on PDO and an in vitro HIPEC model. Our objective is to demonstrate the potential of PM-derived organoids as a predictive surrogate of PM to identify the most effective intraperitoneal drugs for each patient and thereby enhance HIPEC efficacy. To our knowledge, this strategy has never been prospectively evaluated in a formal clinical trial. Nevertheless, our preliminary results demonstrate that this approach is feasible and safe. As seven out of ten patients achieved the endpoint of 12-month PM-free survival, Stage-1 was successfully completed, and the trial has progressed to Stage-2.

A reliable clinical decision-making model for selecting the most active drugs or drug combinations is essential for the implementation of individualized HIPEC treatment. However, no standardized preclinical platform is currently available, as conventional cell lines or patient-derived xenografts fail to fully recapitulate the characteristics of their tumor of origin [16]. PDOs represent an intermediate approach between these models, and have shown promise in predicting therapeutic responses across several tumor types, making them valuable tools for treatment selection [13,37,38,39,40].

We have developed extensive expertise in generating organoids from CRC-PM [14,16]. PDO adhere to a layer of basement membrane extract (Matrigel), thus mimicking the biological and spatial characteristics of microscopic tumor deposits on the peritoneum. In our in vitro HIPEC model, PDO are in directly exposed to the chemotherapy solution, like micrometastases during HIPEC. This model enables us to assess drug activity under the same conditions as the clinical setting [14,15,16]. Moreover, our preclinical platform provides opportunities to test new drugs, perform multiomic analyses, investigate metabolic pathways, and identify new targets for molecular therapies [41].

An exploratory analysis showed consistent inverse associations between PCI and PDO establishment and expansion parameters: PDOs from patients with greater peritoneal disease burden appeared earlier, reached first passage sooner, required fewer passages, and became assay-ready faster. However, these parameters reflect a combination of initial establishment and subsequent expansion rather than standardized measures of intrinsic proliferation, and may be influenced by tissue amount and quality, tumor cell content, post-digestion viability, and the initial number of organoid-forming cells. Furthermore, PCI reflects the extent of peritoneal dissemination and should not be regarded as a direct surrogate of biological aggressiveness. Once accrual and follow-up are complete, associations between PDO growth kinetics and peritoneal recurrence or survival will be investigated in a larger cohort, adjusting for relevant clinical and biological variables where feasible.

Patients received preoperative systemic chemotherapy during the interval needed for PDO development. CRC-PM typically originate from advanced primary tumors [4,5,6], with a substantial risk of systemic failure [42], and perioperative s-CT is aimed at eradicating micrometastases. Preoperative administration offers several advantages, particularly in the context of a demanding procedure, such as CRS-HIPEC. Preoperative s-CT is generally better tolerated, can start earlier, and can be more effective, as surgery induces growth factors potentially stimulating tumor proliferation [43]. Preoperative s-CT can also decrease the peritoneal tumor load, thus increasing the likelihood of achieving a complete cytoreduction while reducing the extent of surgery, which translates into lower postoperative morbidity [44,45]. Lastly, assessment of the response to preoperative s-CT improves patient selection for CRS/HIPEC [46].

Overall, the available literature suggests a benefit of perioperative s-CT, although the evidence remains conflicting [31,47]. Two systematic reviews reported a trend toward increased survival with perioperative s-CT, although the authors emphasized the low quality of the current scientific evidence [48,49]. The recent randomized trial CAIRO-6 demonstrated superior progression-free survival in patients with resectable CRC-PM treated by perioperative s-CT, compared with CRS/HIPEC alone, but no difference in overall survival was observed [50].

We acknowledge several limitations of the present study, including the highly selected population that could not represent the true CRC-PM population, because only selected cases potentially amenable to complete CRS were included. Furthermore, the relatively small sample size limited our ability to fully explore the factors potentially influencing the likelihood of developing TDO, and their differential sensitivity to the various drugs tested. Nevertheless, the study yielded several noteworthy findings. First, oxaliplatin showed meaningful activity only when administered at low concentrations over a long exposure time (120 min), and poor activity at a higher dose over 30 min, as in the Prodige-7 trial [6,51]. This may partially explain the failure of the French trial.

Second, administration of s-CT shortly before PM-tissue collection appeared to reduce the probability of obtaining the TDOs. Accordingly, in Stage-2 we decided to exclude from organoid derivation those patients who had received s-CT after PM diagnosis. Finally, the four patients in whom low-dose/long-course oxaliplatin was identified as the most active drug have never been treated with systemic oxaliplatin. Indeed, acquired resistance following previous systemic oxaliplatin administration has been proposed as an additional explanation for Prodige-7 failure [7].

## 5. Conclusions

The comprehensive precision approach using patient-derived organoids to guide personalized HIPEC is feasible and shows promising early results. High-dose oxaliplatin is poorly active as a drug for HIPEC administration. Stage-1 has been successfully completed, and the trial has proceeded to Stage-2.

## Figures and Tables

**Table 1:** HIPEC drug schedules.

Drug	Dose	Time	Carrier	Ref.	Notes
Oxaliplatin	360 mg/mq	30 min.	Dextrose 5%	28,6	The dose-finding trial was performed with the open-abdomen technique. The dose of 360 mg/mq for the closed abdomen technique was assessed in a large randomized trial (Prodige-7 trial). 5-Fluoruracil (400 mg/mq) and folinic acid (20 mg/mq) are given intravenously 30 min before HIPEC
Oxaliplatin	200 mg/mq	120 min.	Dextrose 5%	29	
Mitomycin-C + cisplatin	3.3 mg/mq/L + 25 mg/mq/L (max 240 mg)	90 min.	Ringer lactate	30–31	Cisplatin dose more than 240 mg was demonstrated to increase both surgical morbidity and systemic toxicity
Mitomycin-C	35 mg/mq	60 min.	Ringer lactate	32	
Doxorubicin + cisplatin	15 mg/L + 45 mg/L (max 240 mg)	90 min.	Ringer lactate	33	.

HIPEC: hyperthermic intraperitoneal chemotherapy.

**Table 2:** Patient characteristics.

Variables	Categories	N.	%
Sex	Male	22	46.8
	Female	25	53.2
Age at enrollment	Mean (SD)	58.8	−12.1
	Median (IQ range)	60.3	(49.6–68.0)
ASA score	2	35	74.5
	3	11	23.4
	4	1	2.1
Charlson score	Mean (SD)	7.6	−1.3
	Median (IQ range)	8	(6.5–8)
Body Mass Index	≤18.5	2	4.3
	>18.5–25	28	59.6
	>25	17	36.1
Primary site	Right	14	29.8
	Left/sigmoid	27	57.4
	Rectum	6	12.8
Histological type	Intestinal	27	57.4
	Mucinous	14	29.8
	Signet ring cell	6	12.8
T category	2	3	6.4
	3	17	36.1
	4a	16	34
	4b	4	8.5
	Not resected	7	14.9
N category	0	14	29.8
	1a/b/c	13	27.6
	2a/b	13	27.6
	Not resected	7	14.9
Peritoneal	Synchronous	27	57.4
metastases	Metachronous	20	42.6
Histological grade	1	6	12.8
	2	15	31.9
	3	19	40.4
	na	7	14.9
Microsatellites/MMR	MSS/pMMR	46	97.9
status	MSI/dMMR	1	2.1
KRAS ^1^	Wild type	20	44.4
	Mutated	25	55.6
NRAS ^1^	Wild type	42	93.3
	Mutated	3	6.7
BRAF ^1^	Wild type	42	93.3
	Mutated	3	6.7
CEA ^1^	≤5 ng/mL	13	28.9
	>5 ng/mL	32	71.1
CA19.9 ^2^	≤37 UI/mL	22	57.9
	>37 UI/mL	16	41.1
Adjuvant syst. chemotherapy ^3^	Done	20	42.6
	Not done	27	57.4

SD: standard deviation; IQ: inter-quartile; MSS: microsatellites stable; MSI: microsatellite instability; pMMR: proficient mismatch repair; dMMR: deficient mismatch repair; ^1^: data available for 45 patients; ^2^: data available for 39 patients; ^3^: at the time of previous primary resections; na: not available.

**Table 3:** Patient-derived organoids development and sensitivity test results.

	TDO Development			Chemosensitivity Test Results				
	No	Yes	*p* Value	mmc	mmc/cis	oxl	*p* Value	None
	12 (27.3%)	31 (72.1%) *		14 (54.2%)	13 (41.9%)	4 (12.9%)		1 (3.2%)
Histological type								
Intestinal	4 (17.4%)	19 (82.6%)	0.208	8 (42.1%)	8 (42.1%)	2 (10.5%)	0.865	1 (5.3%)
Mucinous	5 (35.7%)	9 (64.3%) *		4 (44.4%)	4 (44.4%)	2 (22.2%)		-
SRC	3 (50.0%)	3 (50.0%)		2 (66.6%)	1 (33.3%)	-		-
KRAS								
Wild type	7 (41.2%)	10 (58.8%) *	0.174	7 (70.0%)	3 (30.0%)	-	0.127	1 (10.0%)
Mutated	5 (20.0%)	20 (80.0%)		7 (35.0%)	9 (45.0%)	4 (20.0%)		-
BRAF								
Wild type	11 (28.9%)	27 (71.1%) *	1	13 (48.1%)	11 (40.7%)	4 (14.8%)	0.48	-
Mutated	1 (33.3%)	2 (66.6%)		-	1 (50.0%)	-		1 (50.0%)
Previous syst. Oxalipatin ^1^								
Yes	4 (44.4%)	5 (55.6%)	0.237	3 (60.0%)	2 (40.0%)	-	0.587	-
No	8 (18.2%)	26 (81.8%)*		11 (42.3%)	11 (42.3%)	4 (15.4%)		1 (3.8%)
Adjv. syst. Oxaliplatin ^2^								
Yes	6 (40.0%)	9 (60.0%)	0.287	4 (44.5%)	3 (33.3%)	2 (22.2%)	0.583	-
No	6 (21.4%)	22 (78.6%)*		10 (45.4%)	10 (45.4%)	2 (9.1%)		1 (4.5%)
Any prior oxaliplatin-								
based chemotherapy								
Yes	8 (38.1%)	13 (61.9%)	0.185	6 (46.2%)	5 (38.5%)	2 (15.3%)	0.916	-
No	4 (18.2%)	18 (81.8%) *		8 (44.4%)	8 (44.4%)	2 (11.1%)		1 (5.6%)

TDO: tumor-derived organoids; mmc: mitomycin-C; cis: cisplatin; oxl: oxaliplatin; SRC: signet ring cell; *: one patient was equally sensitive to mitomycin-C and mitomycin-C plus cisplatin; ^1^: systemic chemotherapy delivered to treat peritoneal metastases before tissue collection; ^2^: adjuvant systemic chemotherapy delivered at the time of previous primary resection.

**Table 4:** Clinical characteristics of 10 patients undergoing cytoreductive surgery and patient-tailored HIPEC.

Pt	Sex	Age	Primary Tumor	Site	Histology	s-CT pre	Mutations	PCI	CCR	HIPEC
1	M	46.9	pT3 G2 N2a M1c	rectum	intestinal	folfiri + beva × 6	Nras Q61R	8	0	mmc
2	F	54.5	ypT3 G3 N2a M1c	right	intestinal	folfox + beva × 7	Kras Q61K	15	0	cis/mmc
3	M	66.6	pT3 G3 N0 M0	left	mucinous	folfox + beva × 12	Kras G12A	12	0	cis/mmc
4	F	66.4	pT3 G3 N1b M0	right	intestinal	folfiri + tem. + beva × 12	Kras G12V	3	0	mmc
5	F	57.5	pT3 G1 N1c M0	left	mucinous	folfiri + beva × 12	Kras G12D	12	0	mmc
6	M	54.3	ypT3 G2 N0 M0	rectum	mucinous	folfox + beva × 7	Kras Q61H	3	0	oxl 120
7	F	43.6	pT4a G3 N2b M1c	left	mucinous	folfiri + cetux × 8	-	4	0	mmc
8	M	63.2	pT4a G2 N2a M1c	left	intestinal	folfiri + beva × 12	Kras G12V	11	0	cis/mmc
9	M	69.7	pT4 G2 N1b M0	right	intestinal	folfiri + beva × 13	Kras G12D	11	0	mmc
10	M	41.2	ypT4a Gx N2a M1c	left	intestinal	folfox + cetux × 10	-	15	1	mmc

M: male; F: female; s-CT: systemic chemotherapy; beva: bevacizumab; cetux: cetuximab; tem: tomozolomide; mmc: mitomycin-C; cis: cisplatin; oxl: oxaliplatin.

## Data Availability

The data presented in this study are available on request from the corresponding author due to legal reasons.

## Supplementary Materials

The following supporting information can be downloaded at: https://www.mdpi.com/article/10.3390/cancers18162722/s1, Supplement S1: Study protocol of the OrganoHIPEC clinical trial. Supplement S2: Patient-by-patient data.

## Abbreviations

The following abbreviations are used in this manuscript:

ASA	American Society of Anestesiology
CCR	completeness of cytoreduction
CRC	colorectal cancer
CRS	cytoreductive surgery
CT	computed tomography
CTCAE	Common Terminology Criteria for Adverse Events
dMMR	deficient mismatch repair
EGFR	epidermal growth factor receptor
HIPEC	hyperthermic intraperitoneal chemotherapy
INT	Istituto Nazionale dei Tumori (National Cancer Institute)
IQ	inter-quartile
MSI	microsatellite instability
MSS	microsatellites stable
PCI	peritoneal cancer index
PM	peritoneal metastases
pMMR	proficient mismatch repair
PT	patient tailored
PDO	patient-derived organoids
s-CT	systemic chemotherapy
SD	standard deviation
SRC	signet ring cells
TNM	tumor node metastasis
WHO	World Health Organization
VLS	videolaparoscopic surgery
