# Supplementary material for: Tailoring HIPEC with Patient-Derived Organoids in Colorectal Peritoneal Metastases: Results from the First Stage of the Prospective Phase II OrganoHIPEC Clinical Trial (Clinicaltrials.gov NCT06057298)
Source: Cancers (Basel). 2026 Aug 21;18(16):2722. doi: 10.3390/cancers18162722 (PMC13511742; doi:10.3390/cancers18162722)
Supplement: Supplementary file 1 [file cancers-18-02722-s001.zip › Supplement S1.pdf]

Organoids from colorectal peritoneal metastases to improve cytoreductive surgery and patient-tailored hyperthermic intraperitoneal chemotherapy (HIPEC).

Short title: OrganoHIPEC

VERSION 1.1, 28. 12. 2020

Clinicaltrials.gov number NCT06057298

Sponsor: Fondazione IRCCS istituto Nazionale dei Tumori di Milano

Principal Investigator:

Dario Baratti, MD
Struttura Semplice Tumori Peritoneali
Struttura Complessa Chirurgia Colo-rettale
Fondazione IRCCS Istituto Nazionale dei Tumori
Via Venezian, 1 20133 Milano

Co-principal Investigator:

Marcello Deraco, MD
Responsabile Struttura Semplice Tumori Peritoneali
Struttura Complessa Chirurgia Colo-rettale
Fondazione IRCCS Istituto Nazionale dei Tumori
Via Venezian, 1 20133 Milano

Coordinating Investigators:

Manuela Gariboldi, PhD
Struttura Complessa di Genomica Tumorale
Dipartimento di Ricerca
Fondazione IRCCS Istituto Nazionale dei Tumori
Via Venezian, 1 20133 Milano

Italia Bongarzone, PhD
Struttura Semplice Dipartimentale Meccanismi Molecolari
Dipartimento di Ricerca
Fondazione IRCCS Istituto Nazionale dei Tumori
Via Venezian, 1 20133 Milano

Collaborators

Kusamura Shigeki, MD, PhD
Struttura Semplice Tumori Peritoneali
Struttura Complessa Chirurgia Colo-rettale
Fondazione IRCCS Istituto Nazionale dei Tumori
Via Venezian, 1 20133 Milano

Marcello Guaglio, MD
Struttura Semplice Tumori Peritoneali
Struttura Complessa Chirurgia Colo-rettale
Fondazione IRCCS Istituto Nazionale dei Tumori
Via Venezian, 1 20133 Milano.

Luca Varinelli, PhD
Struttura Semplice Dipartimentale Meccanismi Molecolari
Dipartimento di Ricerca
Fondazione IRCCS Istituto Nazionale dei Tumori
Via Venezian, 1 20133 Milano.

SYNOPSIS

**Title**	**Organoids from colorectal peritoneal metastases to improve cytoreductive surgery and patient-tailored hyperthermic intraperitoneal chemotherapy (HIPEC).**
**Sponsor**	Fondazione IRCCS Istituto Nazionale dei Tumori, Via Venezian, 1 20133 Milano (Italy)
**Principal Investigator**	Dario Baratti, MD<br>S. S. Tumori Peritoneali<br>S. C. di Chirurgia Generale ad Indirizzo Oncologico 2: Colo-rettale<br>Fondazione IRCCS Istituto Nazionale dei Tumori<br>Via Venezian, 1 20133 Milano
**Study phase**	Non comparative, single-center, open-label, clinical study.
**Background**	Peritoneal metastases (MP) are one of the most common site of progression for colorectal cancer (CRC), after liver and lung metastases, and still carry a poorer prognosis than extra-peritoneal metastases. Cytoreductive surgery (CRS) combined with hyperthermic intraperitoneal chemotherapy (HIPEC) to control the microscopic residual disease has improved survival over historical controls and in a successful randomized trial. However, about 50% of patients still experience peritoneal relapse after CRS/HIPEC. Also, the added value of HIPEC has been questioned by a recent randomized trial that showed no survival benefit by adding oxaliplatin (OXL) based HIPEC to CRS and modern systemic chemotherapy (s-CT). Intraperitoneal OXL efficacy issues have been pointed out as a possible reason for this lack of benefit, but there is currently no consensus about the drug of choice for HIPEC, as retrospective studies have provided conflicting results.<br>Since clinical proof that HIPEC can effectively target minimal peritoneal residual disease has been provided by phase III studies in ovarian and gastric cancer, efforts should be made to improve HIPEC efficacy, rather than omitting HIPEC from treatment.<br>Recent advances in 3D culture technology, such as tumor-derived organoids (TDO), provide the opportunity to use more specific and relevant human cancer models. TDO retain genetic and phenotypic characteristics of their tumor of origin, and can be used as *in vitro* surrogate to predict therapeutic responses. Our group and other groups have demonstrated that CRC-PM-derived organoids that closely reflect the original cancer can be established. Also, in recently developed *in vitro* HIPEC models that mimic the same hyperthermic conditions as the clinical setting, drug sensitivity has been demonstrated to vary widely in CRC-PM organoids derived from individual patients. This inefficiency in eliminating microscopic residual disease, at least in a subset of patients, explains the high relapse rates after CRS/HIPEC.<br>We hypothesize that TDO can be used as a representative CRCPM model to test current agents *in vitro* under the same conditions as clinical HIPEC, in order to select patient-tailored (PtT)-HIPEC regimens, and improve the efficacy of this comprehensive treatment approach
**Study objectives**	**The Primary Objective** of this study is to demonstrate the efficacy in controlling peritoneal disease of CRS with individual patient-tailored HIPEC, based on drug sensitivity tests performed in an *in vitro* HIPEC model on CRC-PM-derived organoids.<br>**Secondary Objectives** are to assess feasibility, toxicity and impact on the quality of life (QoV) of CRS with PtT-HIPEC. Overall survival, disease-free survival (peritoneal and distant), and pattern of disease progression after the procedure will also be assessed.
**Study design**	This is a prospective, single-center, open-label, non-comparative clinical trial. The study is conducted according to Fleming's design. Patients with CRC-MP will be treated with diagnostic laparoscopy, peri-operative S-CT, cytoreductive surgery and

	HIPEC according to the current clinical practice. The study will assess a strategy for the use of drugs for HIPEC on a personalized base rather than on a routine base.<br><br>We will prospectively enroll 24 patients with pathologically proven, limited, and surgically resectable CRC-PM, no distant metastases, and no contraindication to CRS/HIPEC. After signature of informed consent, eligible patients will have laparoscopy to confirm PM diagnosis, stage the peritoneal disease, confirm surgical resectability, and provide samples of CRC-PM to develop TDO. Patients will receive 3-6 month preoperative s-CT with targeted agents, according to current guidelines.<br>Patient-derived organoids will be used in an *in vitro* HIPEC model to assess responses of a set of candidate drugs suitable for intraperitoneal administration: oxaliplatin, mitomycin-C, irinotecan, cisplatin, caboplatin, melphalan, doxorubicin. Different concentrations will be tested to generate reproducible dose-response curves. *In vitro* drug response will be used to select the most active HIPEC regimens in individual patients.<br><br>Patients not experiencing disease progression during preoperative s-CT will have CRS and PtT-HIPEC with drugs selected on the organoid-based preclinical model. Additional postoperative s-CT will be administered at the discretion of medical oncologists. Patients will undergo clinical radiological follow-up to record the occurrence of peritoneal recurrences, as well as systemic (extra-peritoneal) metastases, delayed treatment-related toxicities, impact on QdV, and death for any cause.<br><br>All patients screened for the present study will be recorded and prospectively followed. They will constitute three prospective cohorts, as follows:<br>• Cohort 1: patients screened for potentially curative-intent treatment (CRS/HIPEC), but excluded for advanced and/or not surgically resectable disease, or any other cause;<br>• Cohort 2: patients eligible to CRS/HIPEC who do not complete the study protocol due logistic reasons: failure in performing the laparoscopic access, retrieving adequate tumor samples, establishing TDO, performing in vitro drug sensitivity tests, or any other cause. These patients will be offered CRS with mitomycin-C-based-HIPEC, according to our institutional routine practice.<br>• Cohort 3: patients who complete all the study protocol and have CRS with PtT-HIPEC.
**Primary end-point**	The efficacy of CRS and PtT-HIPEC will be assessed by measuring one-year peritoneal metastasis-free survival from the date of the combined procedure for patients included in Cohort 3.
**Secondary end-points**	• Feasibility will be determined as the number of patients who have CRS and PtT-HIPEC (Cohort 3) among all patients selected to be included in the study who sign the informed consent form (Cohort 2 and Cohort 3). The rate between the total number of patients for which tumor samples are retrieved to establish TDO and the actual number of those with available results from *in vitro* drug sensitivity tests will also be calculated.<br>• Overall survival will be measured from the date of CRS and PtT-HIPEC to the date of death for any cause or, for patients still alive at the date of the last available follow-up (Kaplan-Meier).<br>• Disease-free survival will be measured from the date of CRS and PtT-HIPEC to the date of MP diagnosis, systemic metastases or death.<br>• The pattern of disease recurrence will be determined by recording the anatomical

	site and date of onset of peritoneal disease recurrences after CRS and PtT-HIPEC, as well as systemic metastasis (extraperitoneal), and deaths.<br>• QoV will be evaluated with EORTC QLQ-C30 and EORTC QLQ-CR29 questionnaires.
**Number of Subject**	Patients will be enrolled until the number of 24 patients in Cohort 3 (able to have CRS with PtT-HIPEC) is reached.
**Statistics**	Based on literature data, it can be estimated that approximately 60% of patients are expected to be free of CRC-PM one year after CRS/HIPEC. Success from experimental treatment is defined as the absence of clinically and radiologically detectable PM at 12 months from CRS and PtT-HIPEC. The sample size is calculated to reduce the number of patients undergoing treatment that may be ineffective.<br>To demonstrate an absolute increase of 20% in 12-month peritoneal free-survival (from 60% to 80%), with a type 1 error rate (alpha) =0.1, and power (1-ß) =0.8, 24 patients will be treated in 3 years. The null hypothesis will be rejected if 17 or more responses (absence of PM at 12 months) will be observed. Due to failure to establish PM-derived organoids or disease-progression during preoperative s-CT, it is expected that 70% of registered patients will be eligible, resulting in a total of 35 patients to enroll in 3 years.<br>The study will be performed according to the two-stage design described by Fleming and Yung: 16 patients will be enrolled in the first stage of the study. If >10 MP-free patients at 12 months are observed in the first phase, the second phase will begin with the enrolment of 24 total patients. The null hypothesis will be rejected when a total of 18 MP-free patients are observed at 12 months.<br>Exploratory analyses will be performed to compare peritoneal metastasis-free survival, overall survival, progression-free survival, treatment-related toxicity, and QoV between Cohort 2 (CRS and mitomycin-C-based HIPEC on a routine base) and Cohort 3 (CRS and PtT-HIPEC).
**Study population**	Patients with pathologically proven, limited, and surgically resectable CRC-PM, no distant metastases, and no contraindication to CRS/HIPEC.
**Inclusion and exclusion criteria**	Patients will be enrolled according to the following eligibility criteria:<br>**INCLUSION CRITERIA**<br>1) diagnosis of peritoneal metastases from intestinal-type or mucinous colo-rectal adenocarcinoma, by histological/cytological confirmation or clinical/radiological evidence, with or without concomitant elevation of circulating tumor markers.<br>2) limited to moderate peritoneal involvement: peritoneal cancer index (PCI) ≤ 20;<br>3) peritoneal disease potentially amenable to complete surgical cytoreduction;<br>4) no evidence of hepatic, extra-regional nodal, or extra abdominal metastases<br>5) age >18;<br>6) World Health Organization (WHO) performance status ≤2;<br>7) willingness to undergo preliminary laparoscopy, perioperative s-CT, and post-operative follow-up;<br>8) signature of informed consent.<br>**EXCLUSION CRITERIA**<br>1) active sepsis;<br>2) impaired cardiac function (history of previous heart failure or 40% FE);<br>3) impaired renal function (serum creatinine >1.5 normal value or creatinine clearance < 60 ml/min);<br>4) impaired liver function (AST, ALT, bilirubin > 1.5 normal value);<br>5) impaired bone marrow function (leukocytes <4000/mm^3, neutrophils <1500/mm^3, platelets <80000/mm^3);<br>6) impaired lung function (diagnosis of severe COPD or 50% FEV1 or 40% DLCO adjusted for age);

	7) dehydropyrimidine dehydrogenase deficiency**;**<br>8) pregnancy or lactation in progress;<br>9) haemorrhagic diathesis or coagulopathy;<br>10) any other condition or comorbidity that prevents safe administration of systemic chemotherapy (e.g. severe diarrhoeia, stomatitis or ulceration in the mouth or gastrointestinal tract);<br>11) psychiatric or neurological conditions that preclude the procedures of the protocol;<br>12) any contraindication to laparoscopy;<br>13) known hypersensitivity to any of the chemotherapy agents used for HIPEC in the present study and/or to any of their excipients;<br>14) history of previous malignancies treated in the last three years, excluding cutaneous spinocellular carcinoma and/or basocellular carcinoma;<br>15) previous CRS/HIPEC
**Treatment**	**Video laparoscopy (VLS)**<br>VLS will be performed either before or after preoperative s-CT, depending on when the patient is enrolled in the study. Alternatively, it may be done at any time during the s-CT for patients who do not receive bevacizumab, by setting an interval of two weeks from the last cycle and one-week before the next cycle. Representative samples of PM will be taken to develop CRC-PM-derived TDO, and peritoneal disease accurately staged to confirm resectability.<br>**Preoperative systemic chemotherapy (s-CT)**<br>At the discretion of treating medical oncologists, s-CT will consist at least of either four 3-weekly cycles of capecitabine with oxaliplatin (XELOX), six 2-weekly cycles of 5-fluorouracil/leucovorin with oxaliplatin (FOLFOX), six 2-weekly cycles of 5-fluorouracil/leucovorin with irinotecan (FOLFIRI), or six 2-weekly cycles of FOLFOXIRI. Bevacizumab, or anti epidermal growth factor receptor (EGFR) agents in RAS/RAF wild-type tumors may be added. s-CT may be performed exclusively in the preoperative setting, or partly in the preoperative and partly in the postoperative settings.<br>**Cytoreductive surgery (CRS)**<br>CRS and PtT-HIPEC will be scheduled within six weeks and at least 4 weeks after the completion of preoperative s-CT, and at least six weeks after the last administration of bevacizumab. CRS will be aimed at surgically removing all the macroscopic (visible) peritoneal tumor by means of peritonectomy procedures combined with (multi) organ resections, as needed, according to the technique described by Sugarbaker.<br>**Patient-tailored hyperthermic intraperitoneal chemotherapy HIPEC**<br>HIPEC will be performed according to the closed-abdomen technique. The Performer RT (Rand, Medolla, Italy) extra-corporeal circulation device will be used. Perfusion temperature will be 42.5°C, and perfusate volme 4-6 l. Drugs will be selected according to the results of the sensitivity tests on the organoid-based preclinical model. For each drug, the dose delivered to the patient will be based on published phase I trials and large series assessing activity and safety.
**Post-operative follow-up**	To monitor post-operative adverse events, patients will undergo clinical evaluation (daily), and blood chemistry (every other day) until discharge, and then to the same outpatient controls on 7°, 15° and 30° postoperative day. On such occasions the QoV will also be evaluated. For monitoring disease relapse, patients will undergo clinical evaluation, determination of CEA and CA19.9 every three months, and a thoracic-abdomen CT scan every six months.
**Study Duration**	The duration of the study will be 36 months for patient enrolment, followed by 36-month follow-up.

LIST OF ABBREVIATIONS:

ADR= Adverse drug reaction
AE= Adverse Event
ASA= American Society of Anesthesiologists
BMI= Body Mass Index
BSC = best supportive care
CEA= Carcino Embrionary Antigen
CRC= Colo rectal cancer
CRS= Cytoreductive surgery
CT = Computed tomography
CTCAE= Common Terminology Criteria for Adverse Event
ECOG= Eastern Cooperative Oncology Group
EGFR = Epidermal growth factor receptor
FOLFIRI = irinotecan, 5-fluorouracil, and folinic acid
FOLFOX = Oxaliplatin, 5-fluorouracil, and folinic acid
FOLFOXIRI =Oxaliplatin, irinotecan, 5-fluorouracil, and folinic acid
FFPE: Formalin-fixed, paraffin-embedded
HIPEC= Hyperthermic intraperitoneal chemotherapy
IHC: immunohistochemical
ITT= Intention to treat
IRI = irinotecan
MMC = Mitomycin-C
MP= Peritoneal metastasis
NMR = Nuclear magnetic resonance
OXL = Oxaliplatin
PBMC: peripheral-blood mononuclear cells
PCP= Per-protocol
PET = Positron emission tomography
PtT-HIPEC =patient-tailored HIPEC
QoV = Quality of life
SAE= Serious adverse event
S-CT= Systemic chemotherapy
TNM= Tumor Nodes Metastasis
VEGF = Vascular endothelial growth factor
VLS = Video laparoscopy
WHO = World Health Organization
XELOX = Oxaliplatin, oral capecitabine, and folinic acid

INDEX:

Synopsis………2
List of Abbreviations……6

1.0 Background and rationale….…………………………………………………………………………………………9
1.1 Peritoneal metastases from colorectal cancer……………………………………………………………………………9
1.2 Treatment by exclusive systemic chemotherapy…………………………………………………………………………9
1.3 Cytoreductive surgery and hyperthermic intraperitonela chemotherapy (HIPEC)….…………………………10
1.4 The role of HIPEC………12
1.5 Tumor derived organoids (TDO)…………………………………………………………………………………………13
1.6 Rationale for patient-tailored HIPEC……………………………………………………………………………………13
1.7 Rationale for perioperative systemic chemotherapy……………………………………………………………14
2.0 Study Objective……15
2.1 Primary Objective……15
2.2 Secondary Objectives……16
3.0 Study Design………16
3.1 Primary End-Point……18
3.2 Secondary End-Points……19
3.2.1 Feasibility of patient-tailored HIPEC…………………………………………………………………………………19
3.2.2 Overall survival and disease-free survival…………………………………………………………………………19
3.2.3 Safety of CRS and patient-tailored HIPEC…………………………………………………………………………19
3.2.4 Quality of life (QoV)………………………………………………………………………………………………………20
4.0 Patient Enrollment……………………………………………………………………………………………………20
4.1 Selection Criteria………21
4.1.1 Inclusion Criteria……21
4.1.2 Exclusion Criteria……21
4.2 Withdrawal of Subjects……22
4.2.1 Reason for Withdrawal……………………………………………………………………………………………………22
4.2.2 Management of Withdrawal and losses at follow-up………………………………………………………………22
5.0 Operative Procedures…………………………………………………………………………………………………23
5.1 Preoperative Assessment………………………………………………………………………………………………………23
5.2 Laparoscopic Exploration………………………………………………………………………………………………………23
5.2.1 Timing of laparoscopic Exploration …………………………………………………………………………………23
5.2.2 Pre-laparoscopy assessments……………………………………………………………………………………………25
5.2.3 Pre-laparoscopy procedures………………………………………………………………………………………………26
5.2.4 Technique of laparoscopic exploration………………………………………………………………………………26
5.3 Organoid development and *in vitro* drug assays……………………………………………………………………26
5.3.1 Translational research……………………………………………………………………………………………………27
5.4 Preoperative systemic chemotherapy………………………………………………………………………………………29
5.4.1 Timining of preoperative systemic chemotherapy ………………………………………………………………29
5.5 Cytoreductive surgery and patient-tailored HIPEC………………………………………………………………30
5.5.1 Preoperative assessment…………………………………………………………………………………………………30
5.5.2 Preoperative procedures…………………………………………………………………………………………………31
5.5.3 Cytoreductive surgical procedures……………………………………………………………………………………31
5.5.4 HIPEC………32
5.5.6 Personalized drug schedules……………………………………………………………………………………………33
5.5.6 End of combined procedure……………………………………………………………………………………………34
5.6 Postoperative surveillance……………………………………………………………………………………………………34
5.7 Postoperative systemic chemotherapy……………………………………………………………………………………35
5.8 Oncological follow-up………35

5.9 Quality of life assessment……36
6.0 Assessment of adverse events……36
6.1.1 Adverse events……36
6.1.2 Adverse Drug Reaction (ADR)……37
6.1.3 Adverse Event or Serious Adverse Event (SAE)……37
6.1.4 Other events to be treated as SAE……37
6.1.5 Events not to be treated as SAE……37
6.2 Recording and assessment of adverse event……38
6.3 Reporting of Adverse Events……39
6.4 Monitoring of subjects with adverse events……39
7.0 Statistics……39
7.1 Study Population……39
7.2 Sample Size……40
7.3 Statistical analyses……41
8.0 Data Management……42
8.1 Collection of Data……42
8.2 Storage of Data……42
8.3 Conservation of Study Documents……42
9.0 Ethical Aspects……42
9.1 Ethics committee approval……42
9.2 Informed Consent……42
10.0 Administrative Aspects……43
10.1 Confidentiality……43
10.2 Amendments to the protocol……43
10.3 Deviations from the protocol……43
11.0 Benefit and Risk Assessment for participating patients……43
12.0 Bibliography……44

1. BACKGROUND AND RATIONALE

1.1 PERITONEAL METASTASES FROM COLORECTAL CANCER

Colorectal cancer (CRC) is the third most common cancer in the world. In 2012, almost 1.4 million new patients were diagnosed, with an expected incidence of 2.4 million in 2035.[1] The peritoneum is one of the most common sites of tumor progression in CRC patients. In recent population studies, the prevalence of synchronous peritoneal metastases (PM) diagnosed at the same time as the primary tumor is 3.8-4.3%, second only to hepatic metastases. The percentage of patients who experience metachronous PM (after a free time interval from potentially curative treatment) is 3.5-4.2%, second to liver and lung metastases. Since PM are clinically and radiologically difficult to diagnose, literature data are likely underestimated.[2-4] In fact, metachronous PM are diagnosed in 4-19% of patients after potentially curative surgery, up to 44% of patients undergoing re-operative surgery for disease relapse and in 40-80% of cases in autoptic series.[5]

Peritoneal dissemination from CRC or other gastro-intestinal and gynecological malignancies has been traditionally regarded as end-stage disease only amenable to palliation by systemic chemotherapy (s-CT), or best supportive care (BSC). In recent years, better knowledge of the natural history and patterns of tumor dissemination has made increasingly clear that involvement of peritoneal surfaces by CRC may either occur in the absence of hematogenous metastases, or represent the dominant clinical picture.[6]

The use of surgical and/or local-regional therapies to treat metastatic tumor regionally confined to specific organs (such as liver or lung) is one of the most important improvements in modern oncology.[7] Consistently with the current understanding of peritoneal disseminations as local-regional disease entities, the term peritoneal carcinomatosis is being abandoned in favor of peritoneal metastases, that implies the possibility of cure, in contrast with the former that suggests incurable disease. Furthermore, a curative-intent treatment approach has been developed, that combines aggressive cytoreductive surgery (CRS) and intraoperative/perioperative intraperitoneal chemotherapy to control the microscopic residual disease.[6] A similar paradigm shift has occurred in the management of colorectal liver metastases.[7]

1.2 TREATMENT BY EXCLUSIVE SYSTEMIC CHEMOTHERAPY

PM are categorized as stage IV CRC. Accordingly, patients with PM have been traditionally treated by palliative s-CT. However, there are poor literature data on s-CT in CRC-PM, because of both a nihilistic attitude toward these conditions, and poor accuracy of modern radiological tools in staging PM and assessing response to treatment. These patients are often excluded from clinical trials because they have “non-measurable disease”.[6-8]

Historically, median survival was only 5.2–7 months in unselected series treated with BSC, outdated 5-fluorouracil-based s-CT, and/or palliative surgery.[9-11] In a randomized study, median survival of selected patients with potentially resectable CRC-PM was 12.6 months with 5-fluorouracil-based s-CT.[12] More recently, median survival exceeding 30 months have been reported in all-type metastatic CRC by modern combinations, such as XELOX (oxaliplatin, oral capecitabine, and folinic acid), FOLFOX (oxaliplatin, 5-fluorouracil, and folinic acid), FOLFIRI (irinotecan, 5-fluorouracil, and folinic acid), FOLFOXIRI (oxaliplatin, irinotecan, 5-fluorouracil, and folinic acid) plus bevacizumab, or anti epidermal growth factor receptor (EGFR) agents in K-RAS wild-type tumors. However, it remains unclear if such results are replicable in the setting of PM.[13-14] Selected literature series of s-CT for CRC-PM are summarized in Table 1. No randomized trial was conducted in this specific setting. Overall, outcome results suggest that survival depends on patient selection and increasingly aggressive systemic treatments. Oxaliplatin/irinotecan-containing combinations were associated with median survival ranging from 10.1 to 15 months, as compared with 9.0-11.0 months with 5-fluorouracil-based s-CT. The use of monoclonal antibodies against vascular endothelial growth factor (VEGF) or EGFR receptors may result in further improvements (median 15.2–18-2 months).[8,15-21].

It has been demonstrated that even the most effective and modern systemic combinations result in lower survival benefit for CRC-PM, as compared with extra-peritoneal metastases. Franko analyzed 2101 patients included in two cooperative randomized trials of the North Cancer Central Treatment Group (N9741 and N9841). In 364 patients with PM (17.4%) median survival was 12.7 months, as compared with 17.6 months in patients with extra-peritoneal PM (P=0.001).[18] Similar results were found by the same author by reviewing 10553 patients enrolled in 14 randomized trials. 194 patients (1.8%) had isolated CRC-PM, and 1181 (11.2%) had CRC-PM plus other organ involvement. Overall survival of isolated CRC-PM was worse than that of isolated non-peritoneal metastases (P=0·003), and similar to patients with two of more non-peritoneal sites of metastasis (P=0.69) and with PM plus one other site of metastasis (P=0.37).[22] The lower effectiveness of modern s-CT and targeted antibodies was further confirmed by a retrospective analysis of the Dutch randomized studies CAIRO and CAIRO2. Median survival was decreased in patients with PM, as compared with those without PM: 10.4 vs. 17.3 months in CAIRO (P=0.001), and 15.2 vs. 20.7 in CAIRO2 (P < 0.001).[20]

Table 1. Selected series of colorectal peritoneal metastases treated by systemic chemotherapy.

**Author [ref.]**	**Study design**	**Setting**	**Pts (no.)**	**Systemic treatment**	**Median survival (mos)**
Désolneux [16]	Prospective surgical series	Treated by complete CRS	50	FOLFOX/FOLFIRI ± BA	32.4
Zani [21]	Retrospective	Palliative	91	5FU/FA	9.0
			82	FOLFOX/FOLFIRI + BA	16.3
Klaver [19]	Retrospective	Palliative	28	FOLFOX	10.1
			22	FOLFOX + BA	18.2
Klaver [20]	Subset analysis of RCT	Palliative	34	XELOX/XELIRI	10.4
			47	XELOX + BA	15.2
Franko [18]	Subset analysis of RCT	Palliative	364	FOLFOX/FOLFIRI/ irinotecan/oxaliplatin + irinotecan	12.7
Chua [15]	Retrospective series	Palliative	184	BSC	3.0
				5FU/FA	11.0
				FOLFOX/FOLFIRI	15.0
				FOLFOX/FOLFIRI + BA	23.0
Franko [17]	Control arm of non-RCT	Potentially amenable to complete CRS	38	FOLFOX/FOLFIRI ± BA	16.8

CRS: cytoreductive surgery; 5FU: 5-fluorouracil; FA: folinic acid; FOLFOX: 5-fluorouracil, folinic acid and oxaliplatin; FOLFIRI: 5-fluorouracil, folinic acid and irinotecan; XELOX: capecitabine and oxaliplatin, XELIRI: capecitabine and irinotecan; BA: biological agents (bevacizumab, cetuximab, transtuzumab); RCT: randomized controlled trial; BSC: best supportive care

1.3 CYTOREDUCTIVE SURGERY AND HYPERTHERMIC INTRAPERITONEAL CHEMOTHERAPY (HIPEC)

This new curative-intent treatment approach has been developed during the last decade of the past century.[23] CRS may be seen as a tool to maximize response to intraperitoneal chemotherapy, because locally delivered drugs penetrate in tumor tissue not more than a few millimeters. On the other side, the role of local-regional chemotherapy is to preserve the macroscopically complete surgical response by eradicating microscopic residual disease.[24-26]

Cytoreductive surgical procedures have been formalized by Sugarbaker.[23] The loose attachment of parietal peritoneum makes stripping of serosal layers possible by means of diaphragmatic, anterior abdominal wall, omental bursa, and pelvic peritonectomy, depending on PM distribution. Greater and lesser omentectomy are usually performed for oncologic reasons, and to facilitate intra-abdominal drug circulation. Because visceral peritoneum is more intimately attached to underlying structures, tumor implants on visceral surfaces require organ resections, except for liver and pancreas capsulectomy. Cholecystectomy, splenectomy, partial gastrectomy, appendectomy, right, sigmoid, or total colectomy, hysterectomy, bilateral salpingo-oophorectomy, and small bowel resections are the organ resections most commonly performed.

Local-regional chemotherapy has been performed either as Hyperthermic IntraPEritoneal Chemotherapy, (HIPEC), normothermic Early Postoperative Intraperitoneal Chemotherapy (EPIC), or Sequential Postoperative Intraperitoneal Chemotherapy (SPIC). The pharmacological rationale of intraperitoneal administration relies on the dose intensification originated by the presence of a semi-permeable plasma-peritoneal barrier. Intraperitoneal drug delivery allows higher local-regional concentration with minimal systemic toxicity. This advantage is expressed by the high ratio between the area under the curve of intraperitoneal vs. plasma time-concentration.[27] Intraoperative/early postoperative time setting allows optimal distribution throughout the abdominal cavity before postoperative adhesions develop, and tumor cells entrapped in scar tissue give rise to disease recurrence.[23,27] Mild hyperthermia (41–43◦C) has a direct cytotoxic effect by several mechanisms, including DNA repair impairment, protein denaturation, oxidative metabolism inhibition, and increased apoptosis. Also, it increases both activity and penetration into tumor tissue of several antiblastic agents, such as mitomycin-C, doxorubicin, melphalan, and platinum compounds.[28-29]

HIPEC techniques vary widely among centers, in terms of close vs. open-abdomen techniques, drug(s), drug dosage, target temperature, duration, choice and volume of carrier solutions. However, no technical variation has been tested in comparative trials. The choice of antiblastic drugs is based on their clinical efficacy and pharmacokinetics. The perfect agent should be idrophilic, have a high molecular weight to limit its passage through the peritoneal-plasma barrier, high plasma clearance, and mechanisms of action potentiated by hyperthermia. Cell-cycle phase non-specific agents are indicated for this single-shot treatment.[29]

All published randomized or controlled non-randomized studies involving CRS/HIPEC to treat colorectal peritoneal metastases are shown in table 2, along with selected large observational series. A Dutch trial randomized CRC-PM patients to either standard therapy (s-CT and palliative surgery, if needed) or an experimental arm treated by s-CT and CRS/HIPEC.[12] The study demonstrated a significant survival advantage in CRS/HIPEC arm (22.3 vs. 12.6 months; P=0.032) These results were confirmed by four non-randomized studies, that retrospectively compared patients treated by CRS/HIPEC with controls with CRC-PM potentially amenable to surgical cytoreduction, but treated with s-CT and no CRS/HIPEC.[17,31-33] A Swedish study randomized CRS and SPIC vs. s-CT. Overall survival was 25 and 18 months, respectively, in experimental and control arm (P<0.05).[34]

In more recent papers, median survival has increased to about three years, as compared with 22.3 months in CRS/HIPEC arm of the Dutch randomized trial, that was published in 2003.[12] This increase may be explained in part by the fact that patients enrolled in the Dutch trial received outdated 5-fluoruracil-based s-CT, while modern and more effective combinations were administered in the recent series.[17,30-39] Also, criteria to select patients more likely to benefit from CRS/HIPEC have evolved over the years, as it has become increasingly clear that extensive PM, disease not amenable to complete CRS, poor general conditions, and extra-peritoneal metastases are major adverse prognostic factors.[8] Better patient selection, improved surgical technique and perioperative management has also resulted in reduced operative mortality, even though treatment-related complication rates remain relatively high in most international centers.[40-41]

Table 1. Selected series of colorectal peritoneal metastases treated by cytoreductive surgery and HIPEC

**Author** ref.	**Design**	**Treatment**	**Pts (n.)**	**Median surv.(m)**		**Operative morbidity**	**Operative mortality**
Verwaal [12]	Randomized	CRS/HIPEC (mmc)	54	22.3	P=0.032	53.0%	8.0%
		s-CT (No CRS/HIPEC)	51	12.6			
Matheme[33]	Controlled	CRS/EPIC (5-fu)	18	32.0	P=0.02	-	-
		s--CT (No CRS/EPIC)	18	14.0		-	-
Elias [30]	Observational	CRS/HIPEC	523	30.1	-	33.8%	3.3%
Elias [31]	Controlled	CRS/HIPEC (oxl)	48	62.7	P<0.05	-	-
		s-CT (No CRS/HIPEC)	48	23.0		-	-
Franko [17]	Controlled	CRS/HIPEC (mmc)	67	34.7	P<0.001	-	-
		s-CT (No CRS/HIPEC)	38	16.8		-	-
Kuijpers [35]	Observational	CRS/HIPEC (mmc/oxl)	660	33.0		34.5%	3.3%
Esquivel [32]	Controlled	CRS/HIPEC	609	41.0	NS	-	-
		s-CT (No CRS/HIPEC)	275	10.0		-	-
Cashin [34]	Randomized	CRS/SPIC (5-fu)	24	25.0	P<0.05	33.0%	0
		s-CT (No CRS/SPIC)	24	18.0		-	-
Hompes [35]	Observational	CRS/HIPEC (oxl)	48	88.7%	-	52.1	0
Kozman [36]	Observational	CRS/HIPEC (oxl/mmc) ± EPIC	260	35.0	-	35.0%	0.8%
Beal [37]	Observational	CRS/HIPEC (oxl/mmc)	298	32.7	-	20.4%	2.7%
Quenet [39]	Randomized	CRS/HIPEC (oxl)	133	41.7	0.995	24.1%	1.5%
		CRS and NO HIPEC	132	41.2		13.6%	1.5%
Baratti [38]	Controlled	CRS/HIPEC (mmc)	48	34.8	0.702	27.1%	0
		CRS and NO HIPEC	48	39.3		29.2%	0

CRS: cytoreductive surgery; HIPEC: hyperthermic intraperitoneal chemotherapy; EPIC: early postoperative intraperitoneal chemotherapy; SPIC: sequential postoperative intraperitoneal chemotherapy; 5fu: 5-fluorouracil; oxl: oxaliplatin; mmc: mitomycin-C; SCT: systemic chemotherapy; NS: not stated.

1.4 THE ROLE OF HIPEC

Criticisms to the randomized Dutch trial were centered on the impossibility to determine the benefit associated with HIPEC in addition to CRS.[6] Therefore, French investigators designed the Prodige-7 trial to assess the specific value of HIPEC after complete CRS and s-CT.[39] Patients with histologically proven, low-to-moderate extent CRC-PM were randomized after optimal surgery (residual tumor <1mm) to either no HIPEC or oxaliplatin-based HIPEC. All patients received six months of oxaliplatin or irinotecan-containing s-CT, either preoperatively, postoperatively, or both. The primary endpoint was overall survival. Over a six-year period (2008-2014), 265 patients were randomized in 17 French centers. After a 64 month median follow-up, median overall survival was 41.7 months in HIPEC arm, and 41.2 months in non-HIPEC arm (hazard ratio [HR]=1.00; 95% confidence interval [CI]=0.73-1.37; P=0.995). In the two groups, disease-free survival was 13.1 versus 11.1 months (HR=0.91; 95%CI=0.69-1.19; P=0.486), respectively. Sixty-day severe morbidity was higher in HIPEC group (24.1% vs. 13.6%; P=0.030).

A relevant finding of the French trial is the unexpectedly high survival rate in the arm treated by CRS alone, that highlights the leading role of surgery in patients' outcome. It has not to be forgotten that until a couple of decades ago these patients were regarded to as terminally ill patients only to be palliated. Conversely, HIPEC had no effect on overall and recurrence-free survival. This lack of benefit may have several explanations. First of all, the study design: Prodige-7 trial was designed to demonstrate an overall survival increase from 30 (non-HIPEC arm) to 48 months (HIPEC arm), with a two-sided 5% significance level

and 80% power.[41] Presumably, there has been an underestimation of survival in controls, and overestimation of the desired treatment effect. Second, the uncertain efficacy of intraperitoneal oxaliplatin. Although oxaliplatin is one of the drugs of choice for metastatic CRC, factors, such as previous oxaliplatin-based s-CT, may induce alterations in chemosensitivity. Also, exposure time is a major determinant of platinum compounds efficacy, and has to be taken into considerations also the possible adverse effects of carrier solution (5% dextrose), and hyperthermia that may exert antiapoptotic and proliferative effects, induce resistance to chemotherapy and affect tumor immunity.[42-44]

The only comparative non-randomized series assessing the added value of mitomycin-C-based HIPEC is a study by our group. We compared 48 patients treated by perioperative s-CT and CRS with no HIPEC with 48 matched controls treated by s-CT CRS and HIPEC. Analogously to the Prodige-7 trial, survival was not different between groups (34.8 vs. 39.3 months; P=0.702) but, unlike the French trials, severe morbidity was also not different (29.2% vs. 27.1%; P=1.000).[38]

1.5 TUMOR-DERIVED ORGANOIDS (TDO)

Recent advances in 3D culture technology, such as tumor-derived organoids (TDO), provide the opportunity to use more specific and relevant human cancer models in a comprehensive bench-to-bedside strategy.[45] TDO retain genetic and phenotypic characteristics of their tumor of origin, and reflect more closely the original cancer. TDO can be used as *in vitro* surrogate to predict therapeutic responses in a personalized way.[46] Organoids derived from several tumors have shown to respond to standard therapies, thus mimicking patient response.[47-51] Moreover, TDO can be used to develop new therapeutic strategies to circumvent drug resistance.[45] Taken together, these evidences highlight the exceptional value of TDO as clinical tools that can guide prospective cancer patient management. The concordance between TDO and parent tissues can be evaluated using phenotyping technologies to compare molecular and metabolic pathways in TDO and PM, thus providing the opportunity to study response to treatment on individual-patient level.

Our group and another group have demonstrated that CRC-PM-derived organoids can be established, that harbor signatures of the parental CRC (same mutational profile; expression of specific IHC markers of CRC and the stem cell marker LGR5.[52] Also, we and other groups have set *in vitro* HIPEC models to assess drug activity under the same hyperthermic conditions and concentrations as the clinical setting.[52-53]

Ubink have recently tested mitomycin-C and oxaliplatin in 5 CRC-PM-derived organoids, using an *in vitro* model of HIPEC. Drug sensitivity varied widely in individual patient-derived organoids. However, the drug concentrations required to eliminate 50 per cent of the tumour cells (IC50) were higher than the median clinical dose in two of five organoid lines for mitomycin-C, and all five lines for oxaliplatin, indicating a resistance to chemotherapy in a number of patient-derived organoid cultures. This inefficiency in eliminating microscopic residual disease at least in a subset of patients explains the high relapse rates after CRS/HIPEC. Also, combination with inhibitors of the replication checkpoint kinase ATR increased TDO sensitivity to MMC.[53]

1.6 RATIONALE FOR PATIENT-TAILORED HIPEC

CRS combined with HIPEC to control the microscopic residual disease has improved survival of patients with CRC-PM, but at least 50-70% of them still experience peritoneal relapse after combined treatment.[8,17,30-39] Also, the benefit by adding oxaliplatin (OXL)-based HIPEC to CRS and s-CT has been questioned by the Prodige-7 randomized trial, that failed to show any survival advantage in the study arm receiving HIPEC.[39] Intraperitoneal OXL efficacy issues have been pointed out as a possible reason for this lack of benefit,[42-44] but there is currently no consensus on the drug of choice to perform HIPEC in CRC-PM.

Several drugs and drug, selected on the base of their activity in CRC and pharmacokinetics profile suitable for intraperitoneal delivery, has been tested in phase I trials to determine their optimal dosage for HIPEC administration.[29,54] Additionally, most of them have been reported to be associated with good outcome results, low toxicity and high tolerance in large literature series.[30-38,54] Mitomycin-C (MMC) alone or combined with cisplatin is largely used in HIPEC procedures,[55] but has never been directly tested against OXL, and retrospective studies have provided conflicting results.[56-58] The combination of OXL and irinotecan (IRI) has not shown an added value over OXL alone.[59] Other drugs suitable for intraperitoneal administration (carboplatin, melphalan, doxorubicin, 5-fluoruracil) are infrequently used.[8,29,54]

Clinical proof that HIPEC can effectively target minimal peritoneal residual disease has been provided by phase III studies in ovarian and gastric cancer,[60-61] Since variable numbers of patients are reported to experience either long-term survival or early failure after HIPEC with any tested drug or drug combination, it is unlikely that every patient could benefit from the same HIPEC regimen routinely administered to all patients. Analogously, it is unlikely that the same HIPEC regimen could be totally ineffective in every patient.

The above considerations strongly suggest that efforts should be made to improve HIPEC efficacy rather than omitting HIPEC from treatment of CRC-PM. The first and most obvious answer can be a clinical decision model to select the most optimal HIPEC treatment for every individual patient. However, clinical tests to predict who will and who will not benefit from a given intraperitoneal drug or drug combination are lacking. There is an urgent unmet clinical need to select the most active HIPEC regimens at the individual patient level. Representative in vitro models of CRC-PM are required to test the optimal agent, in order to select patent-tailored HIPEC regimens and develop new combination therapies. TDO are specific and highly reliable human cancer models that retain the genetic and phenotypic characteristics of their tumor of origin, reflect more closely the original cancer, and can be used in vitro to predict therapeutic responses.[45]

It has been demonstrated that organoids cultures can be established from CRCPM, and the concordance between TDO and parent CRC-PM tissues has been evaluated using phenotyping technologies (mutational profile; expression of specific immunohistochemical markers, characterization of molecular and metabolic pathways).[52-53] *In vitro* models of HIPEC (developed by our group and other groups) give the opportunity to test drug activity in TDO derived from CRC-PM under conditions comparable with the clinical setting (drug concentrations, temperature, time, type and volume of carrier solutions).[53]

Taken together, all these developments provide the rationale bases to clinically assess a comprehensive strategy involving CRC-PM-derived organoids, and an *in vitro* HIPEC model to test the activity of different drugs to increase HIPEC efficacy by selecting the most active regimens on an individual-patient level.

1.7 RATIONALE FOR PERIOPERATIVE SYSTEMIC CHEMOTHERAPY

The role of perioperative s-CT in the comprehensive treatment of CRC-PM is not yet standardized, as it may improve prognosis, but also induce unnecessary treatment-related side effects, and impact quality of life in patients who do not benefit from the systemic treatment.

Perioperative s-CT has both advantages and disadvantages. Antiblastic agents may eradicate systemic micrometastases, and decrease the risk of systemic failure, that is quite common after CRS-HIPEC.[62] CRC-PM mostly arise from advanced primary tumors with a high risk of systemic spread,[2-6,8] and lymph node positivity is associated with poor outcomes after CRS-HIPEC.[30] As a significant proportion of patients is not amenable to postoperative s-CT because of surgical complications or rapid disease progression, treatment has shifted to neoadjuvant chemotherapy in many centers. Preoperative s-CT may decrease the peritoneal tumor load, and increase the likelihood to obtain a complete cytoreduction, and/or perform less extensive surgery, that in turn potentially leads to lower postoperative morbidity. Also, postoperative s-CT

may eradicate residual cancer cells after CRS/HIPEC.[8,40-41] Lastly, response vs. resistance to preoperative s-CT could improve patient selection for CRS-HIPEC.[63-64] Potentially harmful CRS/HIPEC may be avoided in patients with early progression who are unlikely to benefit due to an unfavorable tumor biology, whereas patients with a favorable response could achieve relevant long-term survival.

On the other hand, the potential drawbacks of perioperative s-CT are as follows: disease progression during preoperative s-CT can lead to unresectable disease, or patients could become ineligible to CRS/HIPEC due to preoperative s-CT-related toxicity.[65-66] Also, perioperative s-CT may hamper its reintroduction at disease recurrence after CRS/HIPEC.[30] Specifically, preoperative administration of bevacizumab has been reported to increase postoperative complications of CRS-HIPEC.[67]

A few literature series have specifically addressed the added value of perioperative s-CT as an adjunct to CRS/HIPEC. Overall, these studies seem to suggest an advantage for patients receiving perioperative s-CT, even though results are conflicting.[37,67-72] Bevacizumab increased survival in one paper,[69] but did not in another paper,[67] that reported increased severe complications after CRS/HIPEC. Kujipers reported a survival advantage with preoperative s-CT, but only in patients with positive lymph-nodes.[70] Two systematic literature reviews have addressed the issue of perioperative s-CT, and conclude that the available data suggest a survival advantage for patients who receive it. However, the authors also highlight the low quality of the current scientific evidence.[73-74] As the timing of sCT is concerned, the existing literature suggests a role for preoperative s-CT (or preoperative plus postoperative s-CT), and may question postoperative s-CT as standard care, but again a low level of evidence has to be taken into consideration.[66,71-72]

Presently, there two different attitudes toward perioperative s-CT: French centers generally administer s-CT preferably in the preoperative setting, and exclude patients with disease progression during s-CT.[30-31] Analogously, both the Lombardy region and Italian Association of Medical Oncologist (AIOM) recommend preoperative s-CT.[75-77] On the contrary, Dutch centers tend to perform upfront CRS/HIPEC, even if s-CT is given to a number of their patients.[62.68-73] Two phase II trials and one phase III trial (CAIRO-6) are presently ongoing in The Netherlands to assess the role of s-CT in association with CRS/HIPEC in CRC-PM patients.[78-80]

In conclusion, the role of complete cytoredcutive surgery in the management of CRC-PM is generally accepted, but the efficiency of HIPEC in eliminating the microscopic residual disease has been questioned by the results of the randomized trial Prodige-7,[39] literature data,[8] and the findings of a recent translational study.[53] However, these data provide the evidence that HIPEC is effective at least in a percentage of patients, that achieve long survival or even cure.[8] Tumor-derived organoids represent *in vitro* CRC-PM model to test current agents, in order to select patent-tailored HIPEC regimens and develop new combination therapies.[45-47]

2. STUDY OBJECTIVE

2.1 PRIMARY OBJECTIVE

The primary objective of this study is to demonstrate the efficacy of patient-tailored (PtT)-HIPEC in controlling peritoneal disease. Personalized HIPEC regimens will be selected by drug sensitivity tests performed in an *in vitro* preclinical HIPEC model on CRC-PM-based organoids derived from the individual patients.

2.2 SECONDARY OBJECTIVES

The secondary objectives are feasibility, toxicity and impact on the quality of life (QoV) of PtT-HIPEC. Furthermore, overall survival, disease-free survival (peritoneal and systemic), and the pattern of disease progression after the combined procedure of CRS and PtT-HIPEC will be assessed.

3. STUDY DESIGN

This is prospective, single-center, open-label, non-comparative clinical trial. The study will prospectively enroll 24 patients with pathologically proven, limited, and surgically resectable CRC-PM, no distant metastases, and no contraindication to CRS/HIPEC. Patients will be treated with diagnostic laparoscopy, peri-operative s-CT, cytoreductive surgery and HIPEC according to the current clinical practice;[75-77] the study will assess a strategy for the use of drugs for HIPEC on a personalized basis, rather than on a routine basis.

Figure 1. General flow-chart of the study

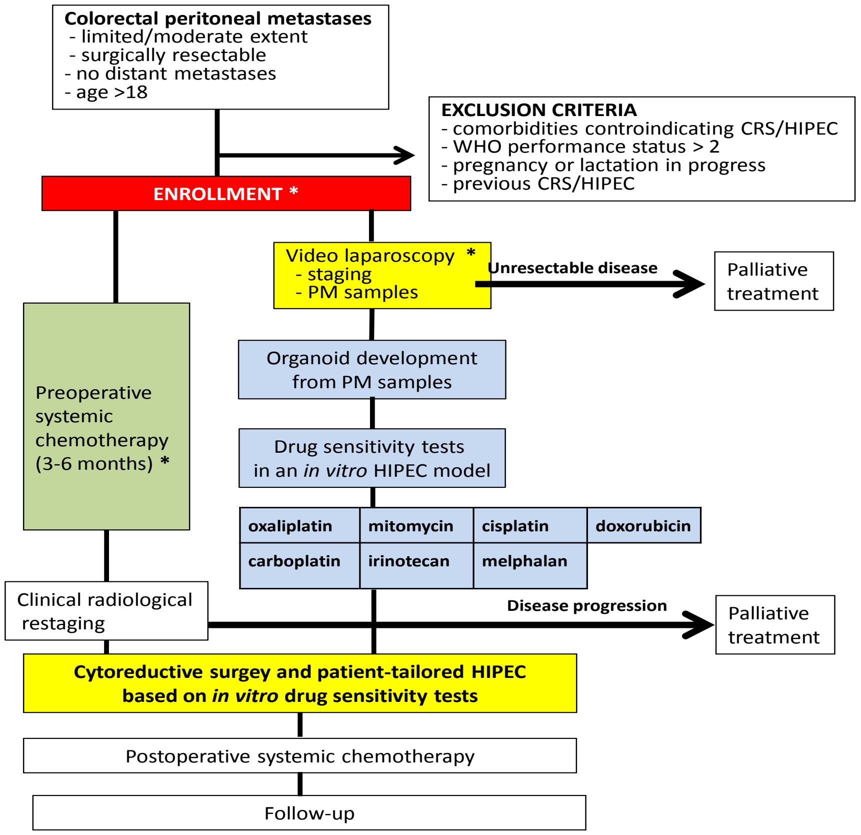

CRS: cytoreductive surgery; HIPEC: hyperthermic intra-peritoneal chemotherapy; WHO: World Health Organization; PM: peritoneal metastases; *: see figure 2.

After signature of informed consent, eligible patients will have laparoscopy to confirm PM diagnosis, stage the peritoneal disease, confirm surgical resectability, and provide samples of CRC-PM to develop TDO. Patients will receive 3-6 month preoperative s-CT with targeted agents, according to current guidelines.[75-77]

Patient-derived organoids will be used in an *in vitro* HIPEC model to select the most active HIPEC regimen on an individual patient level. A set of candidate drugs suitable for intraperitoneal administration will be tested on TDO under HIPEC conditions. Different concentrations will be tested to generate reproducible dose-response curves. *In vitro* drug response will be used to select HIPEC regimens in individual patients.

Figure 2. Flow-chart of the phases of enrollment, preoperative systemic chemotherapy, preliminary videolaparoscopy, and organoid development for *in vitro* drug sensitivity testing.

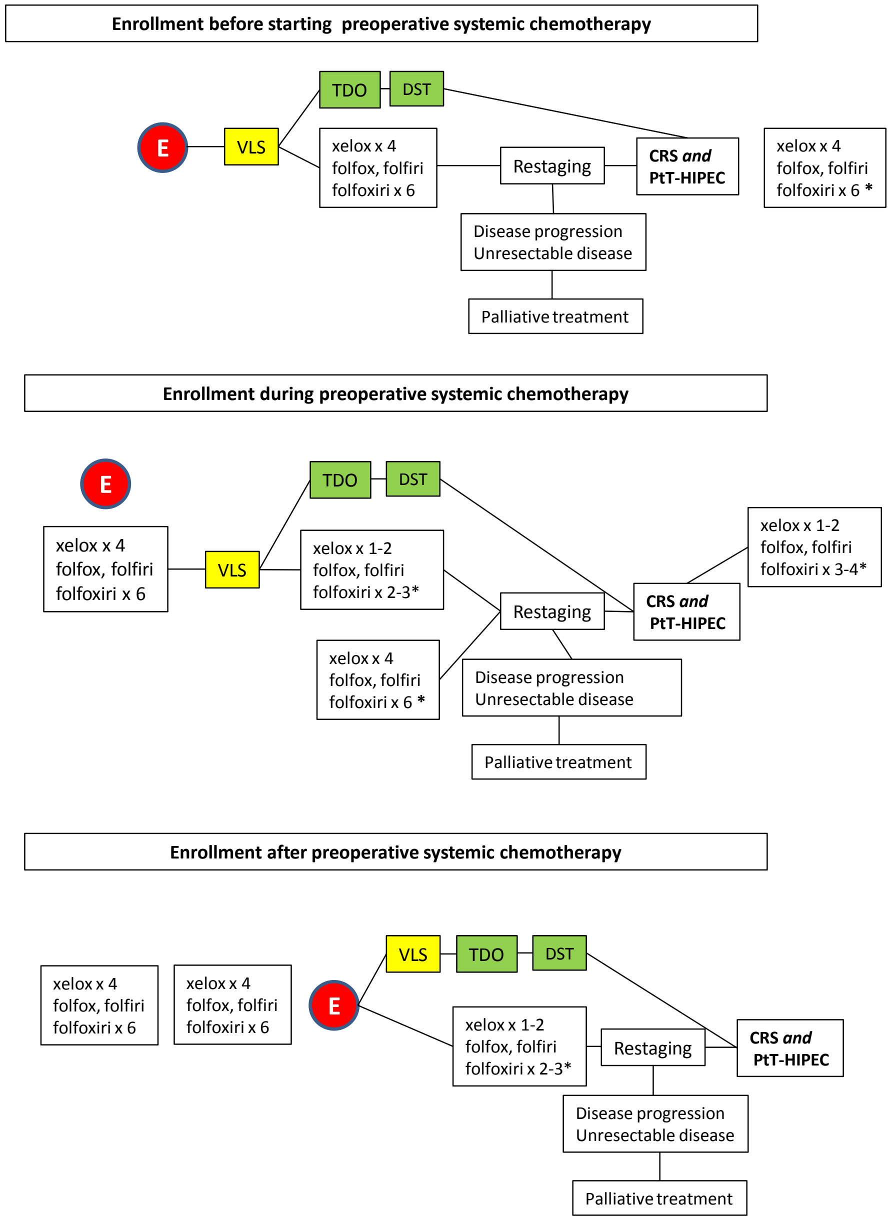

CRS: cytoreductive surgery; PtT-HIPEC: patient-tailored hyperthermic intra-peritoneal chemotherapy; E: enrollment; TDO: tumor-derived organoids; DST in vitro drug sensitivity tests; VLS: video laparoscopy; *: at the discretion of treating medical oncologists.

Patients not experiencing disease progression during preoperative s-CT will have CRS and HIPEC with drugs selected on the organoid-based preclinical model. Additional postoperative s-CT will be administered at the discretion of medical oncologists. Patients will undergo clinical radiological follow-up to record the occurrence of peritoneal recurrences, as well as systemic (extra-peritoneal) metastases, delayed treatment-related toxicities, impact on QdV, and death for any cause.

All patients screened for the present study will be recorded and prospectively followed. They will constitute three prospective cohorts, as follows:

- Cohort 1: patients screened for potentially curative-intent treatment (CRS/HIPEC), but excluded for advanced disease, not surgically resectable PM, disease progression during preoperative s-CT, or any other cause;
- Cohort 2: patients potentially eligible to potentially curative-intent treatment (CRS/HIPEC) who do not complete all the study protocol due to patient refusal, logistic reasons (e.g. failure in performing the laparoscopic access, retrieving adequate tumor samples, establishing TDO, performing *in vitro* drug sensitivity tests), or any other cause. These patients will be offered CRS with mitomycin-C-based-HIPEC, according to our institutional routine practice.
- Cohort 3: patients who complete all the study protocol and have CRS with PtT-HIPEC.

Figure 1 shows a general flowchart of the study, and figure 2 shows in particular the phases of patient enrollment, preoperative s-CT, and preliminary laparoscopic exploration.

3.1 PRIMARY END-POINT

The efficacy of CRS and PtT-HIPEC will be assessed by measuring peritoneal metastasis-free survival from the date of CRS and PtT-HIPEC to the date of recurrent PM diagnosis in patients included in Cohort 3.

Peritoneal metastases will be defined by:

- histological or cytological confirmation of peritoneal tumor after complete CRS and PtT-HIPEC by surgical exploration, imaging-guided biopsy, or endoscopic biopsy;
- computed tomography (CT)-scan, or other relevant imaging study, such as nuclear magnetic resonance (NMR) or fluorodeoxyglucose positron emission tomography (PET)-scan, showing any new lesion compatible with peritoneal tumor localizations (nodules, masses, implants, plaques, or collections involving the peritoneal space) that was not seen at previous examinations, according to modified Response Evaluation Criteria in Solid Tumor Group (RECIST).[81]
- clinical abnormalities (palpable lesions, or other significant objective evidence) consistent with peritoneal tumor localizations that was not seen at previous examinations, with or without concomitant elevation of circulating tumor markers.

Involvement of diaphragm and abdominal wall will be considered as peritoneal recurrence, unless it could be positively demonstrated that the mechanism of development is not tumor cell dissemination from the peritoneal cavity. Ovarian recurrences will be considered as PM.

Systemic metastases will be defined as parenchymal involvement of solid organs within the abdomen (e.g. liver, spleen), or any tumor localization outside the abdomen, either involving solid organs (e.g. lung, bone, brain) or any other anatomical site (e.g. soft tissues). Any effort will be made to discriminate between true hematogenous metastases and implants on the capsule of organs, such as liver or spleen, leading to entrapment of tumor in anatomical fissures, that expand into parenchyma resembling metastatic disease. Involvement of lymph-nodes other than local-regional will be also considered systemic metastases.

An invasive diagnostic approach with imaging-guided or surgical biopsies will be considered for patients with non-conclusive radiological findings. Attempts will be made to confirm the presence of recurrences in

patients with increased tumor markers after CRS/HIPEC. In the event of an isolated increase in circulating tumor marker values at two consecutive follow-up visits, without concomitant clinical and radiological evidence of peritoneal metastases, the patient will be offered the option of a surgical exploration. If no pathological, clinical, or radiological evidence of disease could be demonstrated, the patient will not be considered to have recurrent disease.

3.2 SECONDARY END-POINTS

3.2.1 Feasibility of patient-tailored HIPEC

Feasibility will be determined as the number of patients who have CRS and PtT-HIPEC (Cohort 3) among all patients selected to be included in the study who sign the informed consent form (Cohort 2 and Cohort 3). A number of patient is expected to be not able to complete all the study protocol and have CRS with PtT-HIPEC. The reason for every failure to complete the study protocol will be recorded. These may include (but not be limited to) patient refusal or withdrawal, failure in performing the laparoscopic access, retrieving adequate tumor samples, establishing TDO, performing *in vitro* drug sensitivity tests, or disease progression during or after preoperative s-CT.

The rate between the total number of patients for which tumor samples are retrieved to establish TDO and the actual number of those with available results from *in vitro* drug sensitivity tests on PM-derived TDO will also be calculated. This information will be useful to specifically assess the feasibility of the preclinical phase of the protocol, and better define its operative aspects.

3.2.2 Overall survival and disease-free survival

For Cohort 3 (and Cohort 2), overall survival will be measured from the date of CRS with PtT-HIPEC to the date of death for any cause or, for patients still alive, the date of the last available follow-up. Disease-free survival will be measured from the date of CRS with PtT-HIPEC to the date of diagnosis of peritoneal or systemic (extraperitoneal) metastases or death.

The pattern of disease recurrence after CRS with PtT-HIPEC will be determined by recording both peritoneal recurrences, systemic (extraperitoneal) metastases and deaths. Extra-peritoneal metastases at both haematogenic and lymphatic distances, confirmed histologically or radiologically, will be considered systemic metastases.

Overall survival in Cohort 2 will be compared with Cohort 3. For Cohort 1, overall survival will be measured from the date of CRC-PM diagnosis to the date of death for any cause or, for patients still alive, the date of the last available follow-up. Overall survival from the date of CRC-PM diagnosis will be assessed also for Cohort 2 and Cohort 3, to allow comparison with Cohort 1 (that may be considered to include patients with worse prognosis).

3.2.3 Safety of CRS and patient-tailored HIPEC

Patients will be monitored for adverse events occurring during the intraoperative time and the postoperative hospital stay, irrespective of their potential correlation with the surgical cytoreductive procedures, administration of Pt-T-HIPEC, or both. After discharge, patients will be monitored for late adverse events. Adverse events of all grades, deaths, and hospital readmissions will be recorded for each patient.

Safety of CRS and Pt-T-HIPEC will be assessed as the number of patients who experience severe postoperative complications up to 90 days after the combined procedure. The severity of post-operative adverse events will be defined according to the National Cancer Institute Common Terminology Criteria for

Adverse Events, version 5.0 (NCI-CTCAE).[82] Complications graded as 3 to 5 will be considered severe. Complications (any grade) will be compared with literature data, historical institutional data, and between Cohort 3 (CRS with PtT-HIPEC) and Cohort 2 (CRS with mitomycin-C-based HIPEC).

NCI-CTCAE is a standard tool for describing the toxicity of chemotherapy treatments. It consists of a list of adverse events commonly encountered in clinical trials in oncology, accompanied by a scale of severity of each event, ranging from 1 (mild) to 5 (death). For its completeness and flexibility, it is also commonly used for the assessment of the toxicity profile in local-regional ontological therapies and in particular in intraperitoneal chemotherapy.[82]

The following table summarizes the general principles for defining the severity of adverse events. The third column contains the principles by which NCI-CTCAE is applied to surgical complications.

Table 3: guidelines for severity assessment of adverse events according to NCI CTCAE v.5.0

Grade	Severity of adverse event<br>Oncological therapy complications	Severity of adverse event<br>Surgical complications
1	Mild; asymptomatic or mild symptoms; clinical or diagnostic observations only; intervention not indicated.	No treatment required
2	Moderate; minimal, local or noninvasive intervention indicated; limiting age-appropriate instrumental activities of daily living.	Only pharmacological treatment
3	Severe or medically significant but not immediately life-threatening; hospitalization or prolongation of hospitalization indicated; disabling; limiting self care activities of daily living.	Interventional radiology or endoscopy procedures are required.
4	Life-threatening consequences; urgent intervention indicated.	Surgical procedures or intensive cares are required.
5	Death related to the adverse event.	Death related to the adverse event.

3.2.4 Quality of life

Two questionnaires will be used to measure quality of life, EORTC QLQC30 [83] and QLQ-CR29 [84], whose psychometric properties have been recognized in several international oncological clinical trials. The EORTC QLQ-C30 is a tool designed to be integrated with the disease-specific module. Both questionnaires are available in Italian (see annex 3-6). Qdv will be compared with literature data, historical institutional data, and between Cohort 3 (CRS with PtT-HIPEC) and Cohort 2 (CRS with mitomycin-C-based HIPEC).

4. PATIENT ENROLLMENT

The enrolment in the study will be offered to eligible patients who are diagnosed with peritoneal metastases from colorectal cancer at the Fondazione IRCCS Istituto Nazionale dei Tumori (Milan, Italy). Enrolment will also be open to patients diagnosed with CRC-PM in other centers and referred for clinical evaluation and definitive treatment to the dedicated clinics of the Fondazione IRCCS Istituto Nazionale dei Tumori (Milan, Italy), either at the time of PM diagnosis, during or after the completion of preoperative sCT, provided that they meet the selection criteria and time limits defined by this protocol.

Patients will receive detailed information about the study, and will be informed of the potential risks and benefits of the study procedures and any alternative therapies. They will be asked to sign an informed consent form. The consent form will describe the purpose of the research, the procedures to be performed and the risks and benefits of participation. The investigator will explain the content of the informed consent and verify that the subject has understood the information provided. The investigator will be available to answer any question of clarification. Consent will be voluntary and free from coercion. The investigator will in turn sign the informed consent form. A copy of the consent form will be given to the patient and a copy will be kept in the medical record. Therefore, all patients who will give their consent and sign the informed consent will be enrolled in this study.

4.1 SELECTION CRITERIA

Patients will be enrolled according to the following eligibility criteria:

4.1.1 INCLUSION CRITERIA

1) diagnosis of peritoneal metastases from intestinal-type or mucinous colo-rectal adenocarcinoma, based on at least one of the following:
- histological or cytological confirmation;
- radiological evidence of peritoneal tumor localizations, with both intravenous contrast-enhanced multislice CT-scan and fluorodeoxyglucose PET-scan consistent with peritoneal tumor localizations, with or without concomitant elevation of circulating tumor markers.

2) limited to moderate extent of peritoneal involvement by PM: radiological and laparoscopic peritoneal cancer index (PCI) ≤ 20;[85]
3) peritoneal disease potentially amenable to complete surgical cytoreduction determined by abdominal-pelvic intravenous contrast-enhanced CT-scan;
4) no evidence of systemic metastases at CT-scan of the chest, abdomen and pelvis with an intravenous contrast medium and fluorodeoxyglucose PET-scan;
5) age >18;
6) performance status ≤2 according to the World Health Organization (WHO) score;
7) willingness to undergo preliminary laparoscopy to retrieve peritoneal tumor sample to develop TDO, perioperative systemic therapy and post-operative follow-up;
8) signature of informed consent.

Both patients with PM synchronous with the primary tumor and those with metachronus PM (diagnosed after a free interval from primary surgery) can be enrolled in the study. Patients with synchronous PM who have previously undergone primary resection and/or (partial) debulking of peritoneal disease will also be eligible. The presence vs. previous resection of the primary tumor is not included in the study inclusion/exclusion criteria. Enrolment is allowed for patient with radiologically non-measurable disease. Enrolment is also open for patient who are referred to Fondazione IRCCS Istituto Nazionale dei Tumori (Milan, Italy) after a macroscopically complete surgical resection of either synchronous or metachronous colorectal PM, based on the assumption that microscopic (and often macroscopic) disease is present.

4.1.2 EXCLUSION CRITERIA

1) active sepsis;
2) impaired cardiac function (history of previous heart failure or 40% FE);
3) impaired renal function (serum creatinine >1.5 normal value or creatinine clearance < 60 ml/min);
4) impaired liver function (AST, ALT, bilirubin > 1.5 normal value);
5) impaired bone marrow function (leukocytes <4000/mm^3, neutrophils <1500/mm^3, platelets <80000/mm^3);
6) impaired lung function (diagnosis of severe COPD or 50% FEV1 or 40% DLCO adjusted for age);

7) dehydropyrimidine dehydrogenase deficiency;
8) pregnancy or lactation in progress;
9) haemorrhagic diathesis or coagulopathy;
10) any other condition or comorbidity that prevents safe administration of systemic chemotherapy (e.g. severe diarrhoeia, stomatitis or ulceration in the mouth or gastrointestinal tract);
11) psychiatric or neurological conditions that preclude the procedures of the protocol;
12) any contraindication to laparoscopy;
13) known hypersensitivity to any of the chemotherapy agents used for HIPEC in the present study and/or to any of their excipients;
14) history of previous malignancies treated in the last three years, excluding cutaneous spinocellular carcinoma and/or basocellular carcinoma;
15) previous CRS/HIPEC

4.2 WITHDRAWAL OF SUBJECTS

4.2.1 REASONS FOR WITHDRAWAL

The investigator may withdraw a patient from study treatment and follow-up in the case of:
• death;
• intolerance to study drug;
• Toxicity not related to the drug(s) in study;
• Decision of the patient;
• Decision of the investigators.

Patients who fail to obtain results from drug sensitivity tests on CRC-PM-derived TDO, for any reason (e.g. failed laparoscopic access, adequate tumor tissue impossible to sample, failure in establishing TDO or performing *in vitro* drug sensitivity tests, patient refusal) will be included in Cohort 2. Nevertheless, these patients will be recorded to assess the feasibility of an approach of patient-tailored HIPEC by determining the rate of success (number of patients who will have patient-tailored HIPEC/total number of patients enrolled). Patients excluded from potentially curative-intent treatment for advanced disease at initial evaluation, disease progression during preoperative s-CT, or any other cause will be included in Cohort 1 Most importantly, patients included in Cohort 1 and Cohort 2 will be offered to be treated for their CRC-PM in our center, according to standard treatment protocols that may include CRS with mitomycin-C-based HIPEC or palliative s-CT, depending on the clinical scenario.

Patient accrual will be continued by the investigators until the predetermined number of subjects in Cohort 3 is reached.

Failure to start or complete the assigned perioperative s-CT is not a reason for withdrawal.

Patients are free to withdraw from the study at any time, at their request or at request of their legal representative.

4.2.2 MANAGEMENT OF WITHDRAWALS AND LOSSES AT FOLLOW-UP

When a patient withdraws from the study, the investigator shall record the reasons for withdrawal in the appropriate section of the data sheet. Whenever possible, all patients prematurely withdrawn from the study continue their treatment and/or scheduled follow-up visits. Withdrawn patients will not be replaced, with the exception of patients who fail to complete the phases of the present study protocol that come first of CRS and PtT-HIPEC (preliminary laparoscopic exploration of the abdominal cavity to provide adequate peritoneal tumor tissue sample, establishment of TDO cultures, in vitro drug sensitivity tests).

5. OPERATIVE PROCEDURES

5.1 ELIGIBILITY ASESSMENTS

The following assessments will be carried out to confirm patient eligibility to the study:

- clinical history, including the history of the current pathology, any previous pathologies and current pharmacological treatments;
- WHO Performance Status, height, weight and BMI determination;
- Evaluation of American Society of Anestesiologists (ASA) score;
- Circulating tumor marker determination: CEA, CA19.9;
- Full chest-abdomen CT-scan with intravenous contrast medium;
- Total-body fluorodeoxyglucose PET-scan (other studies, such as NMR, colonoscopy, gastroscopy will be performed if clinically indicated);
- Colonscopy;
- Renal function: creatinine, urea, sodium, potassium, chlorine;
- Hepatic function: AST, ALT, GGT, bilirubin, PT-PTT;
- Cardiac function: ECG and echocardiogram;
- Respiratory function: chest x ray, spirometry;
- Haematological function: hemochrome with formula;
- Nutritional status: albuminemia, protidemia;
- Pharmacogenomics testing: dihydropyrimidine dehydrogenase (DPD) and genetic variants in the dihydropyrimidine dehydrogenase gene (DPYD)
- Beta human corionic gonadotropin (in reproductive age women)
- Informed consent.

An additional 20 ml blood will be drawn and collected for biological determinations (see section 5.3). The diagnostic procedures included in the present study are summarized in table 4.

5.2 LAPAROSCOPIC EXPLORATION

The preliminary video laparoscopy (VLS) of the abdominal cavity is necessary to provide the peritoneal tumor samples needed to establish the TDO. Additionally, the procedure will make it possible to provide additional clinical information to better stage the disease. The laparoscopic exploration will be performed at the Fondazione IRCCS Istituto Nazionale dei Tumori (Milan, Italy).

5.2.1 TIMING OF LAPAROSCOPIC EXPLORATION

Enrolled patients will be allowed to undergo VLS at one of the following time points, depending on when they are enrolled during their clinical history (see also section 5.4.1, and Figure 2):

- Patients who are diagnosed with CRC-PM in our center, or referred to our center soon after CRC-PM diagnosis, but have not yet started s-CT, will have laparoscopic exploration before starting s-CT. VLS will be done within 4 weeks from enrollment. S-CT will be started from 1 to 3 weeks from VLS.

- Patients who are referred to our center after having already started s-CT will have laparoscopic exploration after the completion of either the first four 3-weekly cycles of XELOX, or six 2-weekly cycles of FOLFOX, FOLFIRI, or FOLFOXIRI. These patients will have contrast-enhanced thoracic-abdominal-pelvic CT-scan for oncological restaging before laparoscopic exploration.

- Patients who have already received more than four 3-weekly cycles of XELOX, or six 2-weekly cycles of FOLFOX, FOLFIRI, or FOLFOXIRI will have laparoscopic exploration after the completion of either eight 3-weekly cycles of XELOX, or twelve 2-weekly cycles of FOLFOX, FOLFIRI, or FOLFOXIRI.

- Alternatively, patients who do not receive bevacizumab can undergo laparoscopic exploration at any time during the s-CT, by setting an interval of two weeks from the last cycle and one-week before the next cycle.

Laparoscopic exploration will be done after 4-6 weeks from the completion of s-CT for patients who receive bevacizumab, or after 2 weeks from the completion of s-CT for patients who do not receive bevacizumab.

Table 4: Study procedures.

**Procedures**	**Enrollment**	**On admission for video laparoscopy**	**On admission for CRS and patient-tailored HIPEC**	**Long-term follow-up**
				Months 3, 6, 9, 12, 15, 18, 21, 24, 30, 36.
Informed consent	**X**			
Past medical history	**X**	**X**	Every day during hospital stay	**X**
Physical examination	**X**	**X**	Every day during hospital stay	**X**
Vital signs [1]	**X**	**X**	**X**	**X**
WHO Status	**X**	**X**	**X**	**X**
CEA /CA19.9	**X**		**X**	**X**
ECG, cardiologic assessment	**X**	If clinically indicated	If clinically indicated	If clinically indicated
Blood tests [2]	**X**	**X**	Every other day during hospital stay	**X**
DPD/DPYD testing	**X**			
Beta *(HCG)* [3]	**X**	**X**	**X**	
Chest X-ray	**X**	If clinically indicated	If clinically indicated	If clinically indicated
Thoracic and abdominal CT scan	**X**		**X**	Months 6, 12, 18, 24, 30, 36
Colonoscopy	**x**			Months 12, 36
Adverse event assessment		**X**	**X**	**X**
EORTC QLQ-C30 EORTC QLQ-CR29 questionnaires		**X**	**X**	

[1] Temperature, blood pressure, heart rate, breath rate, O_2 peripheral saturation; [2] White blood cell count, granulocytes, platelet count, and haemoglobin, Na, K, Ca, serum creatinine, BUN, bilirubin, AP, γGT, ASAT, ALAT, APTT, PTT, Total Protein, albumin; [3]: Human Chorionic Gonadotropin (in reproductive age women)

5.2.2 PRE-VIDEOLAPAROSCOPY ASSESSMENT

The following assessments will be carried out on admission for laparoscopic exploration:

• clinical history, including the history of the current pathology, any previous pathologies and current pharmacological treatments;
• Evaluation of WHO Performance Status and ASA score;
• Height, weight and BMI determination;
• Circulating tumor marker determination: CEA, CA19.9;
• Renal function: creatinine, urea, sodium, potassium, chlorine;
• Hepatic function: AST, ALT, GGT, bilirubin, PT-PTT;
• Cardiac function: ECG;
• chest x ray (unless chest CT-scan or x ray has been done during the last 30 days);
• Haematological function: hemochrome with formula;
• Nutritional status: albuminemia, protidemia;
• Informed consent.

5.2.3 PREOPERATIVE PROCEDURES

Antibiotic prophylaxis will be given 30 minutes before surgery with 2 gr cefazolin and 500 mg metronidazole intravenously. Antithrombotic prophylaxis will be administered with a daily dose of 3800 units of nadroparin from the evening prior to surgery. Nasogastric tube, urinary catheter, intravenous access and monitoring will be placed according to the standard procedures in use at the operating teather of the Fondazione IRCCS Istituto Nazionale dei Tumori (Milan, Italy).

5.2.4 TECHNIQUE OF LAPAROSCOPIC EXPLORATION

The purposes of the preliminary VLS will be:
- to provide representative samples of peritoneal tumor to develop CRC-PM-derived TDO;
- to confirm and integrate information from previous imaging on extent and distribution of PM;
- to confirm the possibility to perform a complete surgical cytoreduction;
- to exclude liver or other systemic metastases not detected by previous imaging.

Under general anesthesia, with the patient in gynecological position on the operating table, the laparoscopic procedure will begin with the introduction of a 12 mm trocar in the central quadrant of the abdomen. After induction of a 12 mm Hg CO2 pneumo-peritoneum, additional 5 or 12 mm trocars will be placed under vision. Complete dissection of adhesions (if any) and thorough inspection of the abdominal cavity will be performed, including the Douglas pouch, the sub-diaphragmatic spaces, the sub-hepatic region with the small omentum, the bilateral parieto-colic lodges, the entire small intestine with its mesentery.

Representative tissues of CRC-PM will be sampled from at least three different metastatic peritoneal or omental sites, where technically possible and with a reasonable surgical risk. Every sample should be ≥5 mm in smaller diameter. Samples of unaffected peritoneum will be also taken (> 10 cm from the metastatic lesions). Additional biopsies will be submitted to our Pathology Department for diagnosis confirmation. The presence of viable intraperitoneal tumor cell will be assessed by peritoneal lavage during laparoscopic exploration. Lavage will be performed with 80 ml of saline solution. Two samples of 10 ml will be taken away 5 min later for cytological examination.

The extent and distribution of peritoneal involvement by CRC-PM will be scored using the peritoneal cancer index (PCI). PCI is a semi-quantitative score that rates lesion size from 0 to 3 (no tumor,≤5 mm, >5–50 mm, or >50 mm) in 13 abdominal-pelvic regions, resulting in a numeric score (PCI 0-39).[85] (see annex 7) The

quantification of the peritoneal tumor load is relevant to determine the clinical indication for CRS/HIPEC, as it has become increasingly clear that there is a threshold above which even the most complete CRS is associated with poor prognosis.

The possibility to perform a complete surgical cytoreduction will be confirmed by assessing crucial anatomical areas whose involvement can preclude a compete tumor removal, typically the sub-hepatic region with the hepatic hilum, retroperitoeum, pancreatic head, small intestine with its mesentery, of which a minimal had to be spared to preserve postoperative function. Also, extensive gastric involvement has to be excluded, since total gastrectomy is currently not performed in patients with CRC-PM.

The presence of advanced and/or not surgically respectable peritoneal disease, or other metastatic locations (such as liver or lymph nodes metastases) will result in inclusion of the patient in Cohort 1. Biopsies of metastatic localizations for histo-pathological confirmation will be performed, where technically possible. In case of hepatic metastases or other parenchymal organs (such as the spleen) laparoscopic intraoperative ultrasound will be performed to complete the oncologic staging. Patients excluded from CRS and PtT-HIPEC because of peritoneal or systemic metastases detected during the preliminary laparoscopic exploration will be treated according to current guidelines.[75-77]

The presence of adhesions and their extension will be described in detail in the operator reports. The reasons for any failure in performing the laparoscopic access will be accurately recorded.

5.3 ORGANOID DEVELOPMENT AND *in vitro* DRUG ASSAYS

- **Tissue collection**

In all patients undergoing CRS-HIPEC, tissue specimens of CRCPM will be systematically collected and stored. Metastatic lesions and apparently normal tissue (> 10 cm from the metastatic lesions) will be collected and used for the development of CRC-PM-derived organoids. A second specimen will be frozen in liquid nitrogen for molecular and histopathological analyses. Formalin-fixed, paraffin-embedded (FFPE) blocks will be prepared for immunohistochemical (IHC) analyses, including the immune infiltrate. The remnant material will be used to study genetic alterations and gene/protein expression profiles in both the tumor and in its matched organoid line to identify new targetable pathways.

- **Isolation of peripheral-blood mononuclear cells (PBMCs)**

It is optional to collect PBMC samples at baseline in order to extract normal DNA and immune cells to study the role of the tumor immune microenvironment in PM disease. Ten ml EDTA whole blood for genotyping and PBMCs isolation will be resuspended in cryopreservation medium and stored in liquid nitrogen. PBMCs will be used for co-culture experiment combined with organoids to explore the role of the immune system in the response to HIPEC treatment.

- **Isolation of the ascites**

Ascites is useful to investigate some aspects of the tumor microenvironment and the host response. Ascites is collected by surgical staff in 15 ml or 50 ml tubes, (depending on fluid volume) in presence of heparin to prevent the formation of blood clots. In the lab, the fluid is transferred to conical centrifuge tubes and spun in a balanced centrifuge at 2000 rpm for 5 minutes to pellet the cellular component. Cell-free supernatant is transferred in 15 ml tube and five 1.5 ml microcentrifuge tubes for storage at -80 C°. Cell pellet is resuspended in enough freezing medium for storage at -80 C°. Prior to proteomic analysis, supernatants are centrifuged at 16000g for 30 min for cell debris removal and then 200 µl used for mass spectrometry as described in 6.3.7-9. Aliquots of 100 µl of supernatant are used for metabolite and lipid analysis using

specific mass spectrometry-based methods as described in 5.3.1. Cell pellets are lysed, and extracts used for mass spectrometry analysis as described in 6.3.7-9.

- **Development of CRCPM-derived organoids**

Surgical specimens will be processed and cultured according to the protocol developed by Fuji et al.[86] Biopsies will be digested with standard enzymatic methods and cultured in Matrigel and DMEM F12 medium, in serum free conditions and factors specific of the colonic niche.[86]

- ***In vitro* drug assays on CRC-PM-derived organoids**

To mimic HIPEC treatment, a 96-wells plate will be coated with Matrigel. TDOs will be resuspended in 2% Matrigel/growth media and dispensed into the wells. A dilution series of drugs suitable for HIPEC (OXL, cisplatin, MMC, irinotecan, carboplatin, doxorubicin, melphalan, bevacizumab, 5-fluorouracil) will be dispensed and cell viability assayed. Dose-response curves will be fitted to the luminescent signal intensities as in Garnet et al.[87] and the half-maximal inhibitory concentrations (IC50) will be determined as in van de Wetering et al.[88] The in-vitro drug concentrations, suitable also for the in vivo administration will be evaluated using bioinformatic tools.

5.3.1 TRANSLATIONAL RESEARCH

- **Development of 3D models that mimic CRCPM microenvironment**

CRCPM "lesions on a chip": CRCPM "lesions on a chip" will be obtained by culturing cells obtained from the digestion of CRCPM samples in chambers that reproduce a complex 3D environment. Briefly, new CRCPM samples will be fragmented and digested and the obtained cells will be loaded into A VITVO platform (RIGENERAND, Medolla, Italy).[89] An aliquot of the digested cells will be analysed by FACS, to determine the microenvironment components of the tumor. Subsequently, the model will be treated with different concentrations of therapeutic agents. To verify that the obtained CRCPM "lesions on a chip" reproduce the microenvironment of the patient's lesion, IHC for key lymphoid cell markers, myeloid cells, cancer-activated fibroblasts and mesenchymal-derived cells will be performed both on surgical samples and the "lesion on a chip".

This experimental section is aimed at identifying drug-induced changes and trace molecular vulnerabilities of TDOs derived by each CRCPM patient. We point to provide a basis for the assessment of molecular drug response phenotypes in TDO models. TDOs phenotyping are carried out using high resolution mass spectrometry-based methodology applying multiple approaches to profile proteins, metabolites and lipids. Treated or naive TDO suspensions are generated using enzymatic methods, aliquoted and frozen at -80°C. The proof-of-concept experiments are performed in 3 independent biological replicates and 3 independent technical replicates.

- **LC-MS/MS for metabolite analysis**.

Metabolites are extracted using 50% methanol/30% acetonitrile/20% water.[90] After centrifugation, the surnatants are transferred to mass spectrometry vials and directly analyzed using the UPLC 1290 (Agilent Technologies) coupled to the TripleTOF 5600+ mass spectrometer (SCIEX). MS acquisition is performed in both positive and negative mode. Bioinformatics tools (such as MasterView, SCIEX) and an accurate mass metabolite spectral library, created using the TripleTOF 5600+ LC-MS/MS system, is used for metabolite identification. Metaboanalyst software (www.metaboanalyst.ca) is used for metabolites analyses. An unpaired t-test using the Benjamini Hochberg procedure for controlling false discovery rates (FDR) is used to compare groups. Statistical significance is considered when $p < 0.05$.

- **GC-MS/MS for lipids analysis.**

Fatty acids are prepared from deproteinized TDO pellets in ethanol containing butylatedhydroxytoluene (BHT). Fatty acids are quantified by mass spectrometric methods using a Perkin Elmer Clarus 600D gas chromatograph mass spectrometer. The mass spectrometer operated in mass scan or selected ion-monitoring mode. Peak integration is performed manually, and lipids were quantified against internal standards using standard curves for all compounds as described in De Bortoli et al.[91] Metaboanalyst software (www.metaboanalyst.ca) is used for lipids analyses. An unpaired t-test using the Benjamini Hochberg procedure for controlling false discovery rates (FDR) is used to compare groups. Statistical significance is considered when $p < 0.05$.

Statistical analyses performed for metabolites and lipids datasets include univariate - fold change, t-test, volcano plot, ANOVA, correlation analyses; and multivariate principal component analysis (PCA). Clustering-dendrogram, heatmap, K-means, and self-organizing map (SOM); and supervised classification - random forests and support vector machine (SVM).

- **LC-MS/MS-based protein profiling of TDOs.**

Protein digestion and identification by LC-MS/MS are performed as described previously.[89] Briefly, liquid chromatography coupled with linear ion trap-orbitrap mass spectrometer (LTQ-Orbitrap MS). Mass spectra is analyzed using the MaxQuant software (http://www.maxquant.org). The spectra will be searched for identification by the Andromeda search engine against the human Uniprot sequence database.

For proteins, Enrichr (https://amp.pharm.mssm.edu/Enrichr/enrich) and Toppgene (https://toppgene.cchmc.org/) are used to analyze gene ontology (GO) enrichment in order to identify significant categories of biological processes, molecular functions and cellular components, and network features for prediction of drug targets. Drug Signatures Database (DSigDB, http://tanlab.ucdenver.edu/DSigDB), a free resource providing a list of candidate drugs/compounds enriched in the gene/protein lists, is used to identify interactions from enrichment pathways derived from a single-drug analysis. DSigDB resource is used to examine the enrichment of drug-related protein signature of the response to OXL, cisplatin, MMC, irinotecan, carboplatin, melphalan, bevacizumab, doxorubicin, 5-fluoruracil, to support the exploration of pathway interactions to discover possible interactions of drugs and pathways and identify suitable drugs for repurposing/repositioning. ConsensusPhatDB (http://cpdb.molgen.mpg.de) is interrogated to investigate binary and complex signaling, gene regulatory and drug-target interactions.

- **Study design**

At laparoscopy for PM diagnosis three peritoneal samples will be collected from all the patients enrolled in the phase II study and used for developing CRCPM organoids, constructing “lesion on a chip” models and for conducting genomic/proteomic analyses. CRCPM organoids and “lesion on chips” will be developed during the patient’s preoperative time. Organoids will be obtained from all patients, “lesion on chips” for only about 20 cases starting from the second year of the trial (month 24). The third sample will be used for mass spectrometry analyses. HIPEC treatment with standard drugs will begin as soon as the models are developed. Treatments with drugs that can be repurposed, EMT small molecule inhibitors and actionable targets based on the proteomic analyses conducted will be also tested.

The results of the different treatments will be compared with the reference treatment with Mitomicyn-C. The comparison between results on CRCPM-derived organoids and “lesion on chips” will allow to test the potential impact of tumor microenvironment on HIPEC response. Information about the HIPEC regimen for a patient’s tailored treatment will be achieved in 3 months after laparoscopy.

5.4 PREOPERATIVE SYSTEMIC CHEMOTHERAPY

Perioperative systemic chemotherapy will be administered according to current guidelines.[75-77] At the discretion of the treating medical oncologists, preoperative s-CT consists at least of either four 3-weekly cycles of capecitabine with oxaliplatin (XELOX), six 2-weekly cycles of 5-fluorouracil/leucovorin with oxaliplatin (FOLFOX), six 2-weekly cycles of 5-fluorouracil/leucovorin with irinotecan (FOLFIRI), or six 2-weekly cycles of FOLFOXIRI. Bevacizumab, or anti epidermal growth factor receptor (EGFR) agents in RAS/RAF wild-type tumors may be added.

In case of unacceptable toxicity or contra-indications to oxaliplatin or irinotecan, XELOX or FOLFOX may be switched to FOLFIRI and vice versa. In case of unacceptable toxicity or contraindications to oxaliplatin, XELOX of FOLFOX may be switched to fluoropyrimidine. Dose reduction, co-interventions, and escape medication are not specified *a priori*, but left to the discretion of the treating medical oncologists. Perioperative systemic therapy can be prematurely discontinued due to radiological or clinical disease progression, unacceptable toxicity, physicians decision, or at patients request.

All enrolled patients will have s-CT in the preoperative phase, to comply with our national guidelines,[75-77] and to not remain without treatment during the time interval needed for establishing TDO and performing *in vitro* drug-sensitivity assays. The choice between delivering s-CT exclusively in the preoperative phase, or partly in the preoperative phase and partly in the postoperative phase is left to the treating medical oncologists, because the current study is focused on PtT-HIPEC, and to avoid severe restrictions, in order to facilitate patient accrual.

Failure to start or complete the assigned perioperative s-CT is not a reason for exclusion. Disease progression during previous chemotherapies is also not a reason for exclusion, provided that disease control is achieved during the last s-CT line administered before assessment for CRS and PtT-HIPEC.

5.4.1 TIMING OF PREOPERATIVE SYSTEMIC CHEMOTHERAPY

Enrolled patients will be allowed to undergo preoperative s-CT at one of the following time points, depending on the timing of the preliminary laparoscopic exploration (see also section 5.2.1, and Figure 2):

- Preoperative s-CT can be done **after** laparoscopic exploration in patients who are enrolled in the study soon after CRC-PM diagnosis, and have not yet started their systemic treatment. s-CT will be started from 1 to 3 weeks from laparoscopic exploration. These patients will have either four 3-weekly cycles of XELOX, or six 2-weekly cycles of FOLFOX, FOLFIRI, or FOLFOXIRI before CRS and PtT-HIPEC. After CRS and PtT-HIPEC, they will be evaluated to start postoperative s-CT. Alternatively, these patients may have s-CT exclusively in the preoperative phase (i.e. either eight 3-weekly cycles of XELOX, or twelve 2-weekly cycles of FOLFOX, FOLFIRI, or FOLFOXIRI), at the discretion of the treating medical oncologists.

- Preoperative s-CT can be done **before** laparoscopic exploration. This may be the case of patients who are referred to our center after having already started their s-CT, or those for whom it may be deemed clinically indicated a prompt beginning of the systemic treatment. These patients will have laparoscopic exploration after the completion of either the first four 3-weekly cycles of XELOX, or six 2-weekly cycles of FOLFOX, FOLFIRI, or FOLFOXIRI. Contrast-enhanced thoracic-abdominal-pelvic CT-scan will be done for oncological restaging before laparoscopic exploration. After laparoscopic exploration, at the discretion of the treating medical oncologists, these patients will be allowed to:
    - have no more s-CT before CRS and PtT-HIPEC.

  - have either one to three 3-weekly cycles of XELOX, or two to four 2-weekly cycles of FOLFOX, FOLFIRI, or FOLFOXIRI, as they will be waiting for CRS and PtT-HIPEC. These additional cycles will be delivered without bevcizumab.
  - Complete either eight 3-weekly cycles of XELOX, or twelve 2-weekly cycles of FOLFOX, FOLFIRI, or FOLFOXIRI before CRS and PtT-HIPEC.

- Patients who have already received more than four 3-weekly cycles of XELOX, or six 2-weekly cycles of FOLFOX, FOLFIRI, or FOLFOXIRI will have laparoscopic exploration after the completion of either the eight 3-weekly cycles of XELOX, or twelve 2-weekly cycles of FOLFOX, FOLFIRI, or FOLFOXIRI. Contrast-enhanced thoracic-abdominal-pelvic CT-scan will be done for oncological restaging before laparoscopic exploration. After laparoscopic exploration, at the discretion of the treating medical oncologists, these patients will be allowed to:
  - have no more s-CT before CRS and PtT-HIPEC.
  - have either one to three 3-weekly cycles of XELOX, or two to four 2-weekly cycles of FOLFOX, FOLFIRI, or FOLFOXIRI, as they will be waiting for CRS and PtT-HIPEC. These additional cycles will be delivered without bevacizumab.

- Alternatively, patients who do not receive bevacizumab can undergo laparoscopic exploration **during** their preoperative s-CT, by setting an interval of two weeks from the last cycle and one-week before the next cycle. Analogously to the previous scenarios, these patients will be allowed to complete either four vs. eight 3-weekly cycles of XELOX, or six vs. twelve 2-weekly cycles of FOLFOX, FOLFIRI, or FOLFOXIRI (at the discretion of the treating medical oncologists) before undergoing CRS and PtT-HIPEC.

5.5 CYTOREDUCIVE SURGERY and PATIENT-TAILORED HIPEC

The combined procedure will be performed at the Fondazione IRCCS Istituto Nazionale dei Tumori (Milan, Italy) by the dedicated surgical team highly qualified in the comprehensive management of peritoneal surface malignancies. CRS and PtT-HIPEC will be scheduled within six weeks and at least 4 weeks after the completion of preoperative s-CT, and at least six weeks after the last administration of bevacizumab in order to minimize the risk of bevacizumab-related postoperative complications.[67]

5.5.1 PREOPERATIVE ASSESSMENT

The following assessments will be carried out on admission for cytoreducive surgery and PtT-HIPEC:

• clinical history, including the history of the current pathology, any previous pathologies and current pharmacological treatments;
• Evaluation of ECOG Performance Status and ASA score;
• Height, weight and BMI determination;
• Circulating tumor marker determination: CEA, CA19.9;
• Renal function: creatinine, urea, sodium, potassium, chlorine;
• Hepatic function: AST, ALT, GGT, bilirubin, PT-PTT;
• Cardiac function: ECG;
• chest x ray (unless chest CT-scan or x ray has been done during the last 30 days);
• Haematological function: hemochrome with formula;
• Nutritional status: albuminemia, protidemia;
• Informed consent.

5.5.2 PREOPERATIVE PROCEDURES

Antibiotic prophylaxis will be given 30 minutes before surgery with 2 gr cefazolin and 500 mg metronidazole intravenously. Antithrombotic prophylaxis will be administered with a daily dose of 3800 units of nadroparin from the evening prior to surgery. Nasogastric tube, urinary catheter, intravenous accesses and monitoring will be placed according to the standard procedures in use at the operating teather of the Fondazione IRCCS Istituto Nazionale dei Tumori (Milan, Italy).

5.5.3 CYTOREDUCTIVE SURGICAL PROCEDURES

Under general anesthesia, with the patient in gynecological position, the surgical procedure will begin with a midline laparotomy from the xyphoid to the pubis. Complete dissection of adhesions (if present) and thorough inspection of all the serosal surfaces of the abdominal cavity will be performed. The extent and distribution of peritoneal tumor will be scored using the PCI, and recorded (see annex 7).[85] Intraoperative biopsies for histo-pathological confirmation of suspect PM will be performed.

The presence of previously undetected systemic (extra-peritoneal) metastatic localizations, such as liver or lymph nodes metastases, will be carefully assessed and biopsies submitted for pathological examination. Intraoperative liver ultrasound will be performed to accurately stage hepatic disease.

The cytoreductive surgical procedure is aimed at removing all the macroscopic (visible) peritoneal tumor. Disease localizations will be removed by one or more of the following peritonectomy procedures originally described by Sugarbaker,[23] with further modifications introduced in our center by Deraco et al.[24]

1) left **lateral-anterior peritonectomy**: greater omentectomy, left sub-diaphragmatic and anterior parietal peritonectomy ± splenectomy;
2) **pelvic peritonectomy**: pelvic peritonectomy, total abdominal hysterectomy with bilateral adenessectomy in women, ± sigmoid colectomy;
3) **right upper quadrant peritonectomy**: right sub-diaphragmatic and anterior parietal peritonectomy, Morrison pouch peritonectomy, resection of the round, falciform and triangular hepatic legaments ± liver capsuletomy;
4) **sub-hepatic region peritonectomy**: lesser omentectomy, dissection of the peritoneum covering the epato-duodenal legament, “stripping” of the omental bursa, ± cholecistectomy ± gastric antrectomy;
5) **other procedures/resections:** right, transverse, left or total colectomy, small bowel resection(s), appendectomy, partial emi-diaphragm resection, other tumor resections.

All surgical dissections will be performed according to the technique of high-voltage electro-surgery, using a 2-4 mm ball-tip electro-cautery.[23] This technique allows the surgeon to perform the dissection along a safe plane between the peritoneal layer and the underlying tissues, leaving a margin of heat necrosis that is devoid of viable tumor cells. Electro-surgery minimizes blood loss and, at the same time, the likelihood to disseminate malignant cells.

Small and superficial tumor implants on visceral surfaces will be preferably removed by local excision or cytoreduction by electro-fulguration, to preserve the organs and their postoperative function. Visceral resection will be performed in case of extensive involvement by the tumor. Colon resections to remove primary tumors will be performed according to the oncologic principles of adequate lymph-adenectomy. According to the current practice in our center, bowel anastomoses will be performed before HIPEC to prevent seeding of suture lines, and because it has not been demonstrated in the literature that such a timing is related to increased postoperative complications.[24] All patients will be prepared for the possibility of a bowel stoma. Stomas will be created after the HIPEC, to avoid the loss of perfusate through the abdominal wall during the peritoneal perfusion.

A feeding tube is inserted in selected cases with extensive bowel resections and/or compromised nutritional conditions. Every cytoreductive surgical procedure, including both peritonectomies and organ resections, the total length of the operation, and blood loss were also recorded.

The completeness of the surgical cytoreduction will be classified at the end of the cytoreductive procedures according to the completeness of cytoreduction (CCR) score described by Sugarbaker.[85] The CCR score rates the largest diameter of residual peritoneal tumor implants as follows:

- CCR-0 (macroscopically complete, no visible residual disease);
- CCR-1 (residual disease ≤2.5 mm in any region);
- CCR-2 (residual disease >2.5 mm and ≤25 mm in any region);
- CCR-3(residual disease >25 mm in any region).

The diameter and anatomic site of every residual tumor localization after the surgical cytoreduction will be thoroughly and prospectively recorded, as well as disease extent before the surgical cytoreduction.

5.5.4 HIPERTERMIC INTRA-PERITONEAL CHEMOTHRAPY (HIPEC)

HIPEC will be performed according to the closed-abdomen technique. At the end of the cytoreduction procedure two inflow silicon catheters and two outflow silicon catheters are placed, through separate stab wounds. Inflow catheters are placed in dependent areas, such as the sub-hepatic region, and small pelvis, respectively. Outflow catheters are placed centrally in the abdomen, and in the left sub-diaphragmatic space, respectively, to prevent a preferential flow from inflow to outflow catheters, and allow optimal perfusate and temperature distribution during the perfusion. Temperature sensors are placed in both the inflow and outflow systems, the pelvis and upper abdomen. The skin of the abdominal incision is closed using looped suture in a running, locking fashion. The patient's position is changed every 15 min from Trendelenberg to normal position to reverse Trendelenberg while rotating from left to right.

- **Perfusion device**

The Performer RT (Rand, Medolla, Italy) device will be used to perform HIPEC. This extra-corporeal circulation device has been specifically developed by the manufacturer to perform both peritoneal and limb hyperthermic perfusion with antiblastic agents. The Performer RT is schematically composed of a heat exchanger, two pumps, and an advanced electronic component to manage and monitor in real time inflow and outflow temperature, pressure, volume, and flow rate, to ensure the maximal patient safety during the HIPEC procedure.

- **Carrier solution**

According to our institutional protocol,[24] an isotonic carrier solution is used, consisting of 0.9% NaCl. The carrier solution volume will be calculated on the base of the body surface area, as 3 l/mq. Factors such as the abdominal-pelvic cavity volume, abdominal wall compliance, type and number of abdominal-pelvic organs removed during the CRS (that translate into increased empty space) will be taken into consideration to adjust the calculated volume. The carrier solution volume could be further adjusted depending on anesthesiology issues, and factors related to the HIPEC conduction, as the volume should be large enough to obtain an optimal flow rate and homogeneous fluid and heat distribution throughout the abdomen, without affecting cardiovascular and pulmonary functions, as a consequence of an excessive intra-abdominal pressure. Generally, 4-6 l. of carrier solution are needed.

- **Temperature control**

After filling the abdomen with the carrier solution, and once the whole abdomen is heated to 42-42.5^{o}C, the chemotherapy drug is added. The temperature of the perfusate is measured in the inflow tract, lower abdomen, upper abdomen, and outflow tract. The heat exchanger is set to produce a temperature at the inflow tract up to approximately 44^{0}C. At a perfusion speed of 600/700 ml per minute this translates into a temperature next to the inflow catheter of 42-42.5^{o}C. The temperature in the upper and lower abdomen will take about 30 minutes to rise to its maximum, and stay after that at about 42-42.5^{o}C. To prevent heat trauma to normal tissue the temperature of the inflow catheter will not be increased over 44° C. If temperature does not exceed 40^{0}C at any area, this is remedied by increasing the flow rate of perfusion up to 1l/min., and by, if needed, a change in the position of the catheters to improve distribution.

5.5.5 PERSONALIZED DRUG SCHEDULES

The drug to be administered during HIPEC to the individual patients will be chosen among a panel of potentially effective agents, according to the results of the drug sensitivity tests conducted on patients-derived TDO in an *in vitro* HIPEC model. Drug doses and duration of perfusion for patients enrolled in the present study are based on the results of published dose-finding trials or, if data from phase I studies are not available, from large literature series reporting safety, tolerability, and efficacy of intraperitoneally administered drugs. The intraperitoneal chemotherapy agents will be delivered according to the following schedules, as shown in table 4.

Table 4. HIPEC drug schedules

Drug	Dose	Time	Total dose[1]	Ref.	Note
Oxaliplatin	360 mg/mq	30 m	648 mg	92,39	• The dose-finding trial was performed with the open-abdomen technique. The dose of 360mg/mq for closed-abdomen technique was assessed in a large randomized trial (Prodige-7 trial).<br>• 5-fluoruracil (400 mg/mq) and folinic acid (20 mg/mq) are given intravenously 30 min. before HIPEC
Cisplatin	300 mg/mq<br>max 240mg	60	240 mg	96-98	• cisplatin dose more than 240 mg was demonstrated to increase both surgical morbidity and systemic toxicity
Carboplatin	800 mg/mq	60	1440 mg	99	
Mitomycin-C	35 mg/mq	60	63 mg	93	
Irinotecan	300 mg/ml	30	720 mg	94	• The dose-finding trial was performed with the open-abdomen technique with irinotecan combined with intraperitoneal oxaliplatin.
Melphalan	50 mg/ml	90	92,5 mg	100	
Doxorubicin	15 mg/l	90	75 mg	95	• The dose-finding trial was performed with doxorubicin combined with intraperitoneal cisplatin.

[1]: calculated in an average male patients of 175 cm. height and 70 kg weigth; [2]: calculated on an average carrier solution volume of 5 l.

Patients for whom the results from the drug sensitivity tests on CRC-PM-derived TDO are not available for any reason (e.g. failed laparoscopic access, adequate tumor tissue impossible to sample, failure in establishing TDO or performing in vitro drug sensitivity tests, patient refusal) will be treated with the current HIPEC regimen of choice in our center, consisting of mitomycin-C at a dosage of 35 mg/mq for 60 minutes.

In case two or more drugs result equally active for a given patient, based on the drug sensitivity tests on CRC-PM-derived TDO, the drug to be administered during PtT-HIPEC will be chosen for that individual patient according to the following priority order, based on outcome results and safety data from the literature:

- mitomycin-C
- oxaliplatin
- irinotecan
- cisplatin
- carboplatin
- melphalan
- doxorubicin

5.5.6 END OF COMBINED PROCEDURE

At the end of the perfusion period the inflow of perfusate is stopped and the catheters are allowed to empty the abdominal cavity as much as possible. Than the temporary abdominal skin suture is removed, and the remaining perfusate is manually evacuated. The temperature probes are removed, as well as inflow and outflow catheters. The abdomen is carefully re-explored after HIPEC completion, and every source of bleeding or possible damage to bowel walls or other intra-abdominal organs, as a consequence of mechanical or thermal injury, is accurately controlled. Four silicon drains are placed, through the same stab wounds of inflow and outflow catheters, and used to drain the abdomen postoperatively. Following completion of surgery, the abdomen is closed in the usual way.

All contaminated instruments and tubing were passed off and placed in biohazard containers according to the standard chemotherapy proto-col. The gowns and gloves of all operating staff are changed.

5.6 POSTOPERATIVE SURVEILLANCE

Whenever possible, patients will be awakened immediately after the end of the operation and then kept on intensive monitoring in the intensive care unit until they are deemed stable and ready to return to the hospital ward. Enteral nutrition will be started as soon as possible using the jejunum tube. The central line will be removed as soon as enteral nutrition exceeds 50% of total requirement. Patients discharged from the intensive care unit will continue their postoperative course in the hospital ward. The nasal-gastric tube will be removed as soon as the gastrointestinal function has recovered, to start early food oral intake. The urinary catheter will be removed as soon as adequate level of physical activity and walking autonomy is restored. The abdominal drain will be removed depending on their output and the clinical picture.

If bowel leakage is suspected, a relaparotomy is directly carried out and the leak dealt with. The main danger in this period is a bowel leak concurrent with leucopenia, which usually reaches its nadir on day 10 to 12. Any infectious problems are better solved before that time. Patients who have splenectomy will be given pneumovax, conjugated Hib polysaccharide vaccine and quadrivalent polysaccharide meningococcal vaccine within 10 days after surgery. Admission time is usually around 3 weeks.

During the post-operative period, the following procedures will be performed daily:

- Clinical evaluation with recording of any symptoms reported by the patient;

- Detection of vital parameters (body temperature, blood pressure, heart rate, respiratory rate, peripheral O2 saturation);
- Complete objective examination with inspection of surgical wounds;
- Determination of ECOG Performance Status.

The following tests will be performed on the first post-operative day and then repeated every other day until discharge:

- Renal function: creatinine, urea, sodium, potassium, chlorine.
- Hepatic function: AST, ALT, bilirubin, PT-PTT;
- Haematological function: blood count with formula.

Patient will be discharged on the basis of the following criteria, according to the regular clinical practice in our center: adequate level of physical activity (walking autonomy, self-care, etc.), recovery of sufficient oral food and drink intake, absence of nausea or other gastroenteric disorders, well controlled pain with oral analgesics, patient's willingness to be discharged.

After discharge, patients will be evaluated in the outpatient clinic on the 30° postoperative day to monitor the possible occurrence of adverse events within 30 days

The following procedures will be carried out:

- Clinical evaluation with recording of any symptoms reported by the patient;
- Detection of vital parameters (body temperature, blood pressure, heart rate, respiratory rate, peripheral O2 saturation);
- Complete objective examination with inspection of surgical wounds;
- Determination of ECOG Performance Status;
- Renal function: creatinine, urea, sodium, potassium, chlorine;
- Hepatic function: AST, ALT, bilirubin, PT-PTT;
- Haematological function: blood count with formula.

5.7 POSTOPERATIVE SYSTEMIC CHEMOTHERAPY

Postoperative s-CT will be administered according to current guidelines.[75-77] The choice of s-CT regimen, and the number of cycles to be delivered, will be left to the discretion of the medical oncologist, based on factors such as the preoperative regimens delivered, number of preoperative cycles, response to previous treatment, previous s-CT-related toxicities, and patient conditions after CRS and PtT-HIPEC. Bevacizumab and anti-EGFR agents may be added to postoperative s-CT.

As for preoperative s-CT, postoperative XELOX or FOLFOX may be switched to FOLFIRI and vice versa, and XELOX of FOLFOX may be switched to fluoropyrimidine In case of unacceptable toxicity or contraindications to oxaliplatin. Dose reduction, co-interventions, and escape medication are not specified *a priori*, but left to the discretion of the treating medical oncologist. Postoperative systemic therapy can be prematurely discontinued due to radiological or clinical disease progression, unacceptable toxicity, physicians decision, or at patients request.

5.8 ONCOLOGICAL FOLLOW-UP

Oncological follow-up will be performed on an outpatient basis at the Fondazione IRCCS Istituto Nazionale dei Tumori (Milan, Italy). Follow-up visit will be scheduled four-monthly starting from the date of CRS/HIPEC during the first two years, and six-monthly thereafter until the completion of the third year. During postoperative s-CT, visits will be scheduled at the discretion of treating medical oncologists.

The following procedures will be performed at each control visit
Complete clinical examination;

- Determination of the ECOG Performance Status;
- Circulating tumor marker determination: CEA, CA19.9;
- Renal function: creatinine, urea, sodium, potassium, chlorine;
- Hepatic function: AST, ALT, bilirubin, PT-PTT;
- Haematological function: haemochrome with formula;
- CTCAE (Common Terminology criteria for adverse Events);
- Thoracic and abdominal CT-scan with intravenous contrast medium and/or PET CT scan.

Colonoscopy will be performed at 12, and 36 months postoperatively. Other studies, such as NMR, total-body fluorodeoxyglucose PET-scan, gastroscopy will be performed if clinically indicated. After three years, the patients will be offered to continue postoperative follow-up in the dedicated outpatient clinic of the Fondazione IRCCS Istituto Nazionale dei Tumori (Milan, Italy), according to our institutional policy.

5.9 QUALITY OF LIFE ASSESSMENT

The EORTC QLQ-C30 is a generic quality of life questionnaire, composed of 30 questions, which includes a global health status scale; five scales of functionality (physical, role-playing, emotional, cognitive and social); three scales of symptoms (tiredness, nausea/vomiting and pain), and six single-demand scales (dyspnoea, insomnia, loss of appetite, constipation, diarrhea and financial impact).[83]

QLQ-CR29 is a specific questionnaire for colorectal cancer patients. It consists of 29 questions related to symptoms related to treatment and disease. This questionnaire is structured in 4 scales (body image, frequency of urination, blood and mucus in faeces, frequency of discharges) and 19 single demand scales (anxiety, weight, sexual interest, urinary incontinence, dysuria, abdominal pain, pain in the anal area, swelling, dry mouth, hair loss, taste, flatulence, fecal incontinence, skin irritation, embarrassment, problems in the treatment of stomachs, impotence, dyspareunia).[84]

The questionnaires will be completed by the patient at the time of signature of the informed consent, at the discharge and at the follow-up visits provided.

6 ASSESSMENT OF ADVERSE EVENTS

6.1 ADVERSE EVENTS

An adverse event (AE) is any undesirable medical event in a person who has been given a pharmaceutical product. It does not necessarily have a causal relationship to this treatment. An AE may therefore be any adverse and undesirable sign or symptom (including an altered laboratory finding), or a disease temporally associated with the use of a drug, regardless of whether it is considered to be drug-related.

Events occurring during pre- and post-treatment should be designated as AE. Therefore, safety surveillance (reporting of adverse events) starts when the subject is enrolled in the study (date of signature of informed consent) until the last visit required by the study protocol (end of study, EOS) has been carried out. Therefore, events occurring during the period between the signed informed consent and the start of study drug administration should be designated as adverse events.

6.1.2 ADVERSE DRUG REACTION (ADR)

All adverse and unintended responses to a drug, regardless of its dosage, should be considered as adverse drug reactions (ADR). The term "drug response" indicates that a causal relationship between a medicinal product and an AE is at least a reasonable possibility, that the causal relationship cannot be excluded.

6.1.3 ADVERSE EVENT OR SERIOUS ADVERSE EVENT (SAE)

A serious adverse event (experience) or serious reaction is an undesirable medical event that at any dose of the drug administered:

• hesitating in patient death;
• It is life-threatening;
• Requires hospitalization or prolongation of hospitalization;
• Invalidity or persistent/significant incapacity;
• It is a congenital abnormality or birth defect;
• It is a clinically important event.

Appropriate medical and scientific evaluation will be required to decide whether early warning is appropriate in cases of major medical events which may not be immediately life-threatening or cause death or hospitalization, but may potentially endanger the subject or may require action to prevent one of the other outcomes listed in the above definition. These should usually be considered serious.

6.1.4 OTHER EVENTS TO BE TREATED AS SAE

Exposure to the drug during pregnancy or lactation. In principle, pregnancy and breast-feeding are criteria for exclusion from the trial. If pregnancy occurs during a study, the subject should be immediately discontinued from study medication. The event should be reported promptly and the subject should be followed throughout the course of pregnancy and postpartum. Prenatal and neonatal outcomes should be recorded even if they are completely normal and without AE. The SAE reporting form must be completed, even if pregnancy is not considered as an SAE. In the section of the CRF dedicated to AEs, the entry “no” should be checked in the severity box of the AE.

6.1.5 EVENTS NOT TO BE TREATED AS SAE

The disease progression should not be considered as AE; therefore, it is not an SAE. However, signs and symptoms of tumor progression may meet the criteria for establishing a SAE and, if so, should be reported as such. In case the investigator finds a relationship between drug administration or clinical protocol design and disease progression, the event should be reported as SAE. The occurrence of a new tumor, unrelated to the study disease, should be reported as SAE.

Death itself is a result and therefore should not be considered as SAE. The primary cause of death (the event leading to death) should be recorded and reported as SAE. "Dead/Fatal" will be reported as a result of the respective event. In case no cause of death can be reported (e.g., unexplained death), the death itself could be reported as SAE.

Due to the severity of the pathological condition covered by this study, some events defined as SAE will not be reported rapidly using the SAE related form for monitoring the safety of medicines, that is to say:

- hospitalization for surgery for the treatment of the disease
- hospitalization in order to simplify treatment or study procedures.

However, events should be included in the adverse events section of the CRF

6.2 RECORDING AND ASSESSMENT OF ADVERSE EVENTS

All AEs must be documented in the dedicated section of the CRF. In addition, an SAE reporting form (initial or during follow-up) shall be completed for each SAE.
The following data shall be recorded for each event in the CRF:

- A description of the AE in medical terms, not as reported by the subject;
- The date of onset (starting date);
- The termination date (end date);
- The severity of the sign and/or symptom or clinically significant abnormal laboratory value according to NCI-CTCAE v.5.04.0.[82] If severity classification for a given abnormal sign, symptom or laboratory value is available, the investigator will classify severity as mild (1), moderate (2), severe (3), or life-threatening or disabling

Note: Death (grade 5) as defined by NCI-CTCAE v.5.0 is considered primarily as a result and will be documented accordingly (see below).

For each test product, the following shall be recorded:

- Time of onset of AE for administration of the test product (if applicable);
- The causal relationship with each test product, as assessed by the investigator. One of the decisive factors in the decision is the temporal relationship between AE and the administration of the product concerned. The investigator shall decide whether there is a reasonable possibility that the product under investigation was the cause of EA. The question "Is this AE suspected to be reasonably related to the test product?" It will answer "NO (unrelated)" or "YES (related)", where "related" is defined in the sense that the AE could be medically, pharmacologically or clinically attributed to the experimental product being studied in this protocol.

The actions undertaken for the treatment of AE, according to the following definitions:

- No change in administration;
- Reduction of the dose;
- Temporary interruption;
- Permanent interruption;
- Not applicable.

The outcome of the EEA, as defined below:

- Resolved (missing AE);
- Resolved by sequel (AE has resulted in invalidity / permanent incapacity);
- Not solved yet;
- Not resolved at time of patient death;
- Change in severity (e.g., an EA with no change in severity but re-classified as SAE due to hospitalization);
- fatal (AE causing death).

In the case of severe AE, it shall be reported on which of the following criteria the definition of severe AE is based:

- Dead subject;
- Risk of life;
- Rew or prolonged hospitalization;
- Persistent/significant disability;
- Congenital abnormalities;
- Important medical event.

Main event: only for SAE, should be reported if the SAE is the main event (i.e., the main medical reason for the SAE alert)

If the same adverse events occur on different occasions in any individual, the AE in question should be documented and re-evaluated each time.

Only abnormal laboratory values considered clinically significant by the investigator will be documented in section AE of the CRF. Laboratory parameters which are not within the normal range and which are significantly different from the reference values will be followed, if necessary, until the anomaly has been resolved or the aetiology has been clarified according to the investigator's judgement.

6.3 REPORTING OF ADVERSE EVENTS

Any SAE, related or unrelated to study treatment, should be reported to the Pharmacovigilance Unit of the sponsor institution of the study (Fondazione IRCCS Istituto Nazionale dei Tumori) by the Investigator or a delegated member of the personal investigation within 24 hours of their knowledge of the event.
[E-mail of pharmacovigilance of the institute: Farmacovigilanza.Studispontanei@istitutotumori.mi.it]

All SAEs that will occur during the treatment period and within 30 days of the protocol last treatment should be reported. Any adverse reaction to the late severe drug (SADR), which will occur after this 30-day period, should follow the same reporting procedure.
The investigator will decide whether these events are related to the treatment of the study (i.e., unrelated, unlikely, possible, likely, definitely and un-assessable) and the decision will be recorded on the SAE modules.

6.4 MONITORING OF SUBJECTS WITH ADVERSE EVENTS

Any AE that will occur during a clinical trial and is considered to be related to IMP should be monitored and followed until the outcome is known, whenever possible. Reasonable attempts to obtain this information will have to be documented. It will be the responsibility of the investigator to perform all necessary additional therapeutic measures and follow-up procedures

7 STATISTICS

7.1 STUDY POPULATION

All patients screened for the present study will be recorded and included in three prospective cohorts:

- Cohort 1: patients screened for potentially curative-intent treatment (CRS/HIPEC), but excluded for advanced disease, not surgically resectable PM, disease progression during preoperative s-CT, or any other cause;
- Cohort 2: patients potentially eligible to curative-intent treatment (CRS/HIPEC) who do not complete all the study protocol due to patient refusal, logistic reasons (e.g. failure in performing the laparoscopic access, retrieving adequate tumor samples, establishing TDO, performing in vitro drug sensitivity tests), or any other cause. These patients will be offered CRS with mitomycin-C-based-HIPEC, according to our institutional routine practice.
- Cohort 3: patients who complete all the study protocol and have CRS with PtT-HIPEC.

Cohort 2 and Cohort 3 will be defined as Intention To Treat (ITT) population: includes any person enrolled in the study, regardless of whether they have received the study treatment. Patients who are initially deemed as eligible to potentially curative-intent treatment, but are subsequently excluded due to disease

progression during preoperative s-CT, or any other cause, will be included in Cohort 1, but remain in ITT population.

Cohort 3 will be defined as Per-Protocol (PP) population: includes any patient who has been enrolled and received treatment as required by the protocol.

Enrolled patients may fail to complete the procedures included in the present study protocol for several reasons: failure to perform the preliminary laparoscopic access, retrieve adequate tumor tissue, establish CRC-PM-derived TDO, perform in vitro drug sensitivity tests, disease progression, toxicity or deteriorated clinical conditions during preoperative s-CT, disease found to be not amenable to complete cytoreduction at laparotomy for intended CRS and PtT-HIPEC. The feasibility of CRS with PtT-HIPEC will be calculated as the rate between the actual number of patients who have CRS with PtT-HIPEC (PP population) and the total number of enrolled patients (ITT population).

Patients who are not able to complete the study procedures because they fail to obtain reliable results from drug sensitivity tests on CRC-PM-derived TDO, but are potentially amenable to complete CRS and HIPEC by clinical/radiological assessment, will be treated by CRS/HIPEC according to our routine institutional protocol (i.e. by mitomycin-C-based HIPEC). Patients who are not able to complete the study procedures because of disease-progression during preoperative s-CT, or are otherwise not amenable to complete CRS/HIPEC, irrespective of the results of drug sensitivity tests on CRC-PM-derived TDO, will be treated by palliative s-CT or BSC, according to our current guidelines.[75-77]

The primary study aim (peritoneal metastasis-free survival) and the remaining secondary aims (overall and progression-free survival, toxicity of CRS and PtT-HIPEC, QoV) will be assesses on PP population (i.e. patients who have CRS and PtT-HIPEC). Accrual of patients will continue until the predetermined sample size is reached. Patients who fail to start or complete the assigned perioperative s-CT will not be excluded from PP population. Peritoneal metastasis-free survival, overall survival, progression-free survival, treatment-related toxicity, and QoV will be assesses also in Cohort 2, to allow explorative comparative analyses between Cohort 2 (CRS and mitomycin-C-based HIPEC on a routine base) and Cohort 3 (CRS and PtT-HIPEC).

7.2 SAMPLE SIZE

Based on literature data (see Table 2), about 50-65% of patients are expected to be free of CRC-PM at one year from CRS/HIPEC. In a recent multinstitutional Italian study, in which we participated, 12 month peritoneal disease-free survival was 60% (Sommariva A. Micro-Satellite and RAS/RAF mutational status as prognostic factors in colorectal peritoneal metastases treated with Cytoreductive Surgery and Hyperthermic Intraperitoneal Chemotherapy (HIPEC). Submitted for publication)

Based on these data, a conservative estimation of the benefit expected from experimental intervention is therefore an absolute reduction of PM incidence by 20% (from 40% to 20%) with a relative risk reduction of developing PM by 50%.

To demonstrate an absolute increase of 20% in 12-month peritoneal free-survival (from 60% to 80%), with a type I error rate (alpha) =0.1, and power (1-ß) =0.8, 24 patients will be treated in 3 years. Due to disease-progression during preoperative s-CT or failure to establish PM-derived organoids, it is expected that 70% of registered patients will be eligible, resulting in a total of 35 patients to enroll in 3 years.

The study will be performed according to the two-stage design described by Fleming [101] and Yung [102]. In short, this design envisages that the first stage of the study will be conducted with the aim of excluding from further evaluation treatments that are certainly ineffective, or those that have a success rate below a predetermined threshold level, treating as few patients as possible. The second stage aims to determine

that the treatment being evaluated has a success rate above a designated threshold level and therefore merits further evaluation.

In the present study, treatment success is defined as the absence of clinically and radiologically demonstrable CRC-PM 12 months after primary cancer surgery. The design of the study will follow the model "minimax" described by Simon,[103] that is the preferred design in case it is likely expected that the treatment may give a favorable outcome. It also ensures a reduced sample size in the first phase of the study, so the second phase can be started more quickly.

The null hypothesis that PM-free survival is less than or equal to 60% will be tested against the alternative assumption that MP-free survival is greater than 80% in patients undergoing CRS and patient-tailored HIPEC. To demonstrate a reduction in the relative risk of developing MP by 50%, which is an absolute benefit of 20% in MP-free survival at 12 months, 16 patients will be enrolled in the first stage of the study. If 10 MP-free patients are observed at 18 months, the study will be discontinued and the second phase will not proceed. If, however, >10 MP-free patients at 12 months are observed in the first phase, the second phase will begin with the enrolment of 24 total patients (including the 16 first phase patients). The null hypothesis will be rejected when a total of 18 MP-free patients are observed at 12 months. The design of the study ensures a probability of type 1 error (α) of 5% (level of significance 0.05), and a power (1-β) of 80%. In other words, the likelihood of accepting ineffective treatment as valid will be less than 5%, while the likelihood of erroneously rejecting effective treatment will be less than 20%. These parameters are considered acceptable for a phase II study.

7.3 STATISTICAL ANALYSES

The results of the analyses will be reported in accordance with the CONSORT guidelines.[104] The study population (both ITT population and PP population) will be characterized by descriptive statistics. Continuous variables will be described using the mean, standard deviation, median and range, when appropriate. The categorical variables will be described using frequencies and percentages. The number of patients registered, as well as those actually treated and evaluated, will be summarized by contingency tables. The number of patients who discontinue active treatments and the reasons for discontinuation will also be summarized in the contingency tables.

Progression-free survival is the time between the date of CRS and PtT-HIPEC and the first date of relapse or death, whichever occurs first. Recurrence is defined as the appearance of one or more new lesions, which may be peritoneal, systemic (extra-peritoneal), by imaging or histopathological confirmation. The occurrence of lesions or clinical conditions that cannot be clearly assessed as progression will be discussed among radiologists, surgeons and oncologists. Progression-free survival will be determined based on the date of occurrence provided by the investigator. All deaths with no previous diagnosis of recurrence will be included as an event of disease progression, regardless of the cause or how long it has been since the last known disease assessment. For subjects who remain alive and without recurrence, progression-free survival will be censured at the date of the last evaluable assessment of the disease.

Overall survival is the interval between the date of CRS and PtT-HIPEC and the date of death for any cause. The living subjects will be censored at the last date they were known to be alive.

Peritoneal metastasis-free survival, overall survival, and progression-free survival will be calculated using the Kaplan-Meier method.[105] Results will be expressed in terms of 1-year, 2-year, and 3-year survival rates with 95% confidence interval. Peritoneal metastasis-free survival, overall survival, and progression-free survival will be compared between Cohort 2 and Cohort 3 using two-tailed log-rank test. Patient, tumor and treatment-related variables (including complication rates) will be compared among cohorts using Chi-square test, Fisher's exact test, 1-way ANOVA test, or Student T test, as appropriate. All statistical

analyses will be conducted via SPSS, version 22.0.0 for Windows (SPSS, Chicago, IL). No interim analysis is planned.

8 DATA MANAGEMENT

8.1 COLLECTION OF DATA

Collected data will be compared with the original data from the study monitor. The data contained in the data sheets will be recorded in an electronic database by the data manager of the coordination centre. Checks will be carried out to assess the accuracy of data recording in the electronic database in relation to the data collected. No analysis of the results will be carried out before the accuracy of the recorded data is verified.

8.2 STORAGE OF DATA

Electronic and paper data storage will be entrusted to the Study Coordinator Centre.

8.3 CONSERVATION OF STUDY DOCUMENTS

In order to ensure the confidentiality of the data collected in electronic form, the database will be protected by a known access password to the data manager of the study, the statistician and the head of the Coordination Centre. Data protection against possible damage and/or loss will be ensured by periodic backup.

9. ETHICAL ASPECTS

9.1 ETHICS COMMITTEE APPROVAL

Before initiating the trial, investigators must obtain a written favorable opinion of the Internal Review Board (IRB) and the Local Ethics Committee for the Study Protocol, the informed written consent form, the recruitment procedures of the subject and any other written information to be provided to the subjects. All correspondence with IRB should be kept in the investigator's file.

Before implementing any modification of the protocol, it is necessary to obtain the written approval IRB. The only circumstance in which an amendment may be initiated prior to the IRB approval is that the modification is necessary to eliminate immediate apparent dangers for patients. In such a case, IRB shall be informed as soon as possible. The investigator responsible shall ensure that the study is conducted in accordance with both the Helsinki Declaration and the laws of the country. The protocol has been written and the study will be conducted according to the International Council for Harmonization of Technique Requirements for Pharmaceuticals for Human Use (ICH) guidelines for good clinical practice. The protocol and its annexes are subject to review and approval by the independent ethics committee of the Fondazione IRCCS Istituto Nazionale dei Tumori. The study may begin to recruit subjects only after official approval from the competent authorities, Ethics Committee and AIFA.

9.2 INFORMED CONSENT

Each patient will be informed in advance during an interview during which the doctor responsible will:
a) inform the patient about the state of his illness;
b) inform the patient about conventional therapeutic opportunities;

c) inform the patient of the availability of alternative treatment, and in particular define the treatment objectives, the specific risks associated with treatment and the likelihood of success;
d) clarify to the patient that a more intensive diagnostic and therapeutic program is needed than conventional ones.

The patient, after being informed about the course of treatment, must sign a written consent recognising full awareness of the risk/benefit of the proposed treatment and thus accepting the proposed treatment program.

10. ADMINISTRATIVE ASPECTS

10.1 CONFIDENTIALITY

The confidentiality of patients enrolled in the study should be protected by all trial investigators. The study protocol, documentation, data collected and all other information will be kept strictly confidential. No information about the study or data will be given to unauthorized third parties without prior written permission of the study director.

All data sheets, reports and other documents leaving the trial site should only be identified by an identification code to maintain subject confidentiality. No clinical information will be released without the subject’s written permission, except for what is necessary for monitoring.

10.2 AMENDMENTS TO THE PROTOCOL

This study will be conducted in accordance with the current version of the protocol. Any changes to the document or informed consent form relating to the scientific purpose, study design, patient safety, or which may influence the willingness of the participants to continue the study participation is considered an amendment, and therefore should be described and presented as an amendment to the protocol and/ or informed consent. All amendments will be submitted to the IEC for approval before they become operational.

10.3 DEVIATIONS FROM THE PROTOCOL

All protocol deviations should be recorded in the patient’s and CRF medical records and reported to the main investigator, who will assess their relevance. Deviations that may affect the validity of the study results, patient safety or the ethics of the trial will be reported to CEI. When deviations from the protocol require a revision of the protocol, the protocol will be amended as indicated in Section 11.2.

11. BENEFIT AND RISK ASSESSMENT FOR PARTICIPATING PATIENTS

The foreseeable benefit of the patient-tailored HIPEC approach is a possible improvement of the site-regional control of the tumor, that is a reduction of the risk of developing recurrent peritoneal metastases after perioperative systemic chemotherapy and resection to curative intent combined with HIPEC to treat the microscopic residual peritoneal tumor. As a result, an improvement in survival is expected, as compared to the standard treatment of moderate extent and surgically respectable CRC-PM consisting of perioperative s-CT, complete cytoreductive surgery and HIPEC with administration of an antiblastic agent on a routine base (i.e. the same drug or drug combination for all patients). The resistance to chemotherapy, based on the individual tumor biologic and molecular features, translates into the inefficiency in eliminating microscopic residual disease at least in a subset of patients that explains the high relapse rates after CRS and traditional HIPEC

In addition to the normal risks related to perioperative systemic chemotherapy, extensive cytoreductive surgery and HIPEC, there may be an additional risk of complications related to the preliminary laparoscopic access to stage the disease and provide the peritoneal tumor samples to develop CRC-PM-derived organoids. However, given the limited invasiveness of such a procedure, the operative risk has to be deemed as relatively low. Another potential risk for patients participating to the present study is that the *in vitro* sensitivity tests performed on CRC-PM-derived organoids could fail to provide reliable results. In this case, a drug identified *in vitro* as the most active antiblastic agent for that individual patient could not actually be the most active one *in vivo*, or at least it could be less active than a drug administered on a routine base.

12. REFERENCE

1. Ferlay J, Soerjomataram II, Dikshit R, Eser S, Mathers C, Rebelo M, et al. Cancer incidence and mortality worldwide: sources, methods and major patterns in GLOBOCAN 2012. Int J Cancer. 2014;136:E359–86.
2. Lemmens VE, Klaver YL, Verwaal VJ, et al. Predictors and survival of synchronous peritoneal carcinomatosis of colorectal origin: a population-based study. Int J Cancer. 2011;128:2717-25.
3. van Gestel YR, et al. Metachronous peritoneal carcinomatosis after curative treatment of colorectal cancer. Eur J Surg Oncol. 2014; 40: 963–9.
4. Segelman J, Granath F, Holm T, et al. Incidence, prevalence and risk factors for peritoneal carcinomatosis from colorectal cancer. Br J Surg. 2012;99:699-70.
5. Koppe MJ, Boerman OC, Oyen WJ, Bleichrodt RP. Peritoneal carcinomatosis of colorectal origin: incidence and current treatment strategies. Ann Surg. 2006;243:212-22.
6. Sugarbaker, PH, Ryan, DP. Cytoreductive surgery plus hyperthermicperioperative chemotherapy to treat peritoneal metastases from colorectalcancer: standard of care or an experimental approach. Lancet Oncol. 2012;13,e362–9.
7. Tomlinson JS, Jarnagin WR, DeMatteo RP, Fong Y, Kornpra, P, Gonen M, et al. Actual 10-year survival after resection of colorectal livermetastases defines cure. J. Clin. Oncol. 2007;25, 4575–4580.
8. Baratti D, Kusamura S, Pietrantonio F, Guaglio M, Niger M, Deraco M. Progress in treatments for colorectal cancer peritoneal metastases during the years 2010-2015. A systematic review. Crit Rev Oncol Hematol. 2016;100:209-22.
9. Chu DZ, Lang NP, Thompson C, Osteen PK, Westbrook KC. Peritoneal carcinomatosis in nongynecological malignancies: a prospective study ofprognostic factors. Cancer 1989;63, 364–367.
10. Jayne Fook DGS, Loi C, Seow-Choen F. Peritoneal carcinomatosis fromcolorectal cancer. Br. J. Surg. 2002;89, 1545–1550.
11. Sadeghi B, Arvieux C, Glehen O, Beaujard AC, Rivoire M, Baulieux J, et al. Peritoneal carcinomatosis from non-gynecologic malignancies: results ofthe EVOCAPE 1 multicentric prospective study. Cancer 2000;88, 358–363.
12. Verwaal VJ, van Ruth S, de Bree E, et al. Randomized trial of cytoreduction and hyperthermic intraperitoneal chemotherapy versus systemic chemotherapy and palliative surgery in patients with peritoneal carcinomatosis of colorectal cancer. J Clin Oncol. 2003;21:3737-43.
13. Pietrantonio F, Cremolini C, Petrelli F, Di Bartolomeo M, Loupakis F, Maggi C, et al. First-line anti-EGFR monoclonal antibodies in panRAS wild-typemetastatic colorectal cancer: a systematic review and meta-analysis. Crit. Rev.Oncol. Hematol. 2015;96, 156–166.
14. Loupakis F, Cremolini C, Masi G, Lonardi S, Zagonel V, Salvatore L, et al. Initial therapy with FOLFOXIRI and bevacizumab for metastatic colorectalcancer. N. Engl. J. Med. 2014;371, 1609–1618.
15. Chua TC, Morris DL, Saxena A, Esquivel J, Liauw W, Doerfer J, et al. Influence of modern systemic therapies as adjunct to cytoreduction and perioperative intraperitoneal chemotherapy for patients

with colorectal peritoneal carcinomatosis: a multicenter study. Ann. Surg. Oncol. 2011;18,1560–1567.

16. Désolneux G, Mazière C, Vara J, Brouste V, Fonck M, Béchade D, et al. Cytoreductive surgery of colorectal peritoneal metastases: outcomes after complete cytoreductive surgery and systemic chemotherapy only. PLoS One2015; 10:e0122816.
17. Franko J, Ibrahim Z, Gusani NJ, Holtzman MP, Bartlett DL, Zeh 3rd, HJ. Cytoreductive surgery and hyperthermic intraperitoneal chemoperfusion versus systemic chemotherapy alone for colorectal peritoneal carcinomatosis. Cancer 2010;116, 3756–3762.
18. Franko J, Shi Q, Goldman CD, Pockage B, Nelson B, Goldberg R, et al. Treatment of colorectal peritoneal carcinomatosis with systemic chemotherapy: a pooled analysis of north central cancer treatment groupphase III trials N9741 and N9841. J. Clin. Oncol. 2012;30, 263–267.
19. Klaver YL, Simkens LH, Lemmen, VE, Koopman M, Teerenstra S, Bleichrodt RP, et al. Outcomes of colorectal cancer patients with peritoneal carcinomatosis treated with chemotherapy with and without targeted therapy. Eur. J. Surg. Oncol. 2012;38:617–623.
20. Klaver YL, Leenders BJ, Creemers GJ, Rutten HJ, Verwaal VJ, Lemmens VE, et al. Addition of biological therapies to palliative chemotherapy prolongs survival in patients with peritoneal carcinomatosis of colorectal origin. Am. J. Clin. Oncol. 2013;36:157–161.
21. Zani S, Papalezova K, Stinnett S, Tyler D, Hsu D, Blazer 3rd, DG. Modest advances in survival for patients with colorectal-associated peritonealcarcinomatosis in the era of modern chemotherapy. J. Surg. Oncol. 2013; 107,307–311.
22. Franko J, Shi Q, Meyers JP, Maughan TS, Adams RA, Seymour MT, Saltz L, Punt CJA, Koopman M, Tournigand C, Tebbutt NC, Diaz-Rubio E, Souglakos J, Falcone A, Chibaudel B, Heinemann V, Moen J, De Gramont A, Sargent DJ, Grothey A; Analysis and Research in Cancers of the Digestive System (ARCAD) Group. Prognosis of patients with peritoneal metastatic colorectal cancer given systemic therapy: an analysis of individual patient data from prospective randomised trials from the Analysis and Research in Cancers of the Digestive System (ARCAD) database. Lancet Oncol. 2016;17:1709-1719.
23. Sugarbaker PH. Peritonectomy procedures. Ann Surg. 1995;221, 29–42.
24. Deraco M, Baratti D, Kusamura S, Laterza B, Balestra MR. Surgical technique of parietal and visceral peritonectomy for peritoneal surfacemalignancies. J. Surg. Oncol. 2009;100, 321–328.
25. Bao P, Bartlett D. Surgical techniques in visceral resection and peritonectomy procedures. Cancer J. 2009;15:204–211
26. Mehta SS, Bhatt A, and Glehen O. Cytoreductive Surgery and Peritonectomy Procedures. Mehta SS, Bhatt A, Glehen O. Cytoreductive Surgery and Peritonectomy Procedures. Indian J Surg Oncol. 2016;7:139-151.
27. Van der Speeten K, Stuart OA, Sugarbaker PH. Pharmacology of perioperative intraperitoneal and intravenous chemotherapy in patients with peritoneal surface malignancy. Surg. Oncol. Clin. N. Am. 2012; 21, 577–597.
28. Sticca RP, Dach BW. Rationale for hyperthermia with intraperitnealintraoperative chemotherapy agents. Sug. Oncol. Clin. N. Am. 2005;12, 689–700.
29. Kusamura S, Elias D, Baratti D, Younan R, Deraco M. Drugs carrier solutions and temperature in hyperthermic intraperitoneal chemotherapy. J.Surg. Oncol. 2008;98, 247–252.
30. Elias D, Gilly F, Boutitie F, Quenet F, Bereder JM, Masvelt B, et al. Peritoneal colorectal carcinomatosis treated with surgery and peri-operative intraperitoneal chemotherapy: retrospective analysis of 523 patients from a multicenteric French study. J Clin Oncol. 2010; 28: 63-68.
31. Elias D, Lefevre JH, Chevalier J, Brouquet A, Marchal F, Classe JM, et al. Complete cytoreductive surgery plus intraperitoneal chemohyperthermia with oxaliplatin for peritoneal carcinomatosis of colorectal origin. J Clin Oncol. 2009;27: 681-685.
32. Esquivel J, Lowy AM, Markman M, Chua T, Pelz J, Baratti D, et al. The American Society of Peritoneal Surface Malignancies (ASPSM) multiinstitution evaluation of the Peritoneal Surface Disease Severity

Score (PSDSS) in 1013 patients with colorectal cancer with peritoneal carcinomatosis. Ann Surg Oncol. 2014; 21:4195-4201.

33. Mahteme H, Hansson J, Berglund A, Påhlman L, Glimelius B, Nygren P, Graf W. Improved survival in patients with peritoneal metastases from colorectal cancer: a preliminary study. Br J Cancer. 2004;90:403-7
34. Cashin PH, Mahteme H, Spång N, Syk I, Frödin JE, Torkzad M, Glimelius B, Graf W. Cytoreductive surgery and intraperitoneal chemotherapy versus systemic chemotherapy for colorectal peritoneal metastases: A randomised trial. Eur J Cancer. 2016;53:155-62.
35. Hompes D, D'Hoore A, Van Cutsem E, Fieuws S, Ceelen W, Peeters M, et al. The treatment of peritoneal carcinomatosis of colorectal cancer withcomplete cytoreductive surgery and hyperthermic intraperitonealperoperative chemotherapy (HIPEC) with oxaliplatin: a Belgian multicentreprospective phase II clinical study. Ann. Surg. Oncol. 2012;19, 2186–2194.
36. Kozman MA, Fisher OM, Rebolledo BJ, Parikh R, Valle SJ, Arrowaili A, et al. CEA to peritoneal carcinomatosis index (PCI) ratio is prognostic in patients with colorectal cancer peritoneal carcinomatosis undergoing cytoreduction surgery and intraperitoneal chemotherapy: a retrospective cohort study.J Surg Oncol. 2018;117: 725-736
37. Beal EW, Suarez-Kelly LP, Kimbrough CW, Johnston FM, Greer J, Abbott DE, et al. Impact of neoadjuvant chemotherapy on the outcomes of cytoreductive surgery and hyperthermic intraperitoneal chemotherapy for colorectal peritoneal metastases: a multi-institutional retrospective review. J Clin Med. 2020;9:3
38. Baratti D, Kusamura S, Azmi N, Guaglio M, Montenovo M, Deraco M. Colorectal Peritoneal Metastases Treated by Perioperative Systemic Chemotherapy and Cytoreductive Surgery With or Without Mitomycin C-Based HIPEC: A Comparative Study Using the Peritoneal Surface Disease Severity Score (PSDSS). Ann Surg Oncol. 2020;27:98-106.
39. http://ascopubs.org/doi/abs/10.1200/JCO.2018.36.18_suppl.LBA3503?af=R.
40. Baratti D, Kusamura S, Iusco D, Bonomi S, Grassi A, Virzì S, et al. Postoperative complications after cytoreductive surgery and hyperthermic intraperitoneal chemotherapy affect long-term outcome of patients with peritoneal metastases from colorectal cancer: a two-center study of 101 patients. Dis. Colon Rectum. 2014;57: 858-868.
41. Chua TC, Yan TD, Saxena A, Morris DL. Should the treatment of peritoneal carcinomatosis by cytoreductive surgery and hyperthermic intraperitoneal chemotherapy still be regarded as a highly morbid procedure?: a systematic review of morbidity and mortality. Ann Surg. 2009;249: 900-907.
42. Ceelen W. HIPEC with oxaliplatin for colorectal peritoneal metastasis: The end of the road? ELSO 2019;45:400-2.
43. Quenet F. Colorectal peritoneal carcinomatosis: What is the future of HIPEC? EJSO. 2018;44: 1847-1848
44. Evrard S. Autopsy of an expert consensus: End of hyperthermic intraperitoneal chemotherapy in colorectal carcinomatosis. EJSO. 2018;44:1845-1846.
45. Tuveson D, Clevers H. Cancer modeling meets human organoid technology. Science. 2019;364.952-955.
46. Sachs N, et al. A Living Biobank of Breast Cancer Organoids Captures Disease Heterogeneity. Cell. 2018;172:373-386.e10.
47. Lee SH, et al.Tumor Evolution and Drug Response in Patient-Derived Organoid Models of Bladder Cancer. Cell. 2018 ;173:515-528.e17.
48. Hill ASJ, et al. Prediction of DNA Repair Inhibitor Response in Short-Term Patient-Derived Ovarian Cancer Organoids. Cancer Discov. 2018;8:1404-1421.
49. Tiriac H, et al. Organoid Profiling Identifies Common Responders to Chemotherapy in Pancreatic Cancer. Cancer Discov. 2018;8:1112-1129.
50. Vlachogiannis G, et al. Patient-derived organoids model treatment response of metastatic gastrointestinal cancers. Science. 2018;359:920-926.

51. Pauli C, et al. Personalized In Vitro and In Vivo Cancer Models to Guide Precision Medicine. Cancer Discov. 2017;7:462-477.
52. Bozzi F, et al. MIF/CD74 axis is a target for novel therapies in colon carcinomatosis. J Exp Clin Cancer Res. 2017;36:16.
53. Ubink I, et al. Organoids from colorectal peritoneal metastases as a platform for improving hyperthermic intraperitoneal chemotherapy. Br J Surg. 2019;106:1404-1414.
54. Yurttas C, Hoffmann G, Tolios A, et al. Systematic Review of Variations in Hyperthermic Intraperitoneal Chemotherapy (HIPEC) for Peritoneal Metastasis from Colorectal Cancer. J Clin Med 2018; 7:567-598.
55. Van Ruth S, Verwaal VJ, Zoetmulder FAN. Pharmacokinetics of intraperitoneal mitomycin C. Surg. Oncol. Clin. N. Am. 200312, 771–780.
56. Prada-Villaverde A, Esquivel J, Lowy AM, Markman M, Chua T, Pelz J, et al. The American Society of Peritoneal Surface Malignancies evaluation ofHIPEC with Mitomycin C versus Oxaliplatin in 539 patients with colon cancerundergoing a complete cytoreductive surgery. J. Surg. Oncol. 2014; 110, 779–785.
57. Hompes D, D'Hoore A, Wolthuis A, Fieuws S, Mirck B, Bruin S, et al. The use of Oxaliplatin or Mitomycin C in HIPEC treatment for peritonealcarcinomatosis from colorectal cancer: a comparative study. J. Surg. Oncol. 2014;109, 527–532.
58. Leung V, Huo YR, Liauw W, Morris DL. Oxaliplatin versus mitomycin C for HIPEC in colorectal cancer peritoneal carcinomatosis. Eur J Surg Oncol. 2017;43:144–9.
59. Quenet F, Goéré D, Mehta S, Roca L, Dumont F, Hessissen M, et al. Results of two bi-institutional prospective studies using intraperitonealoxaliplatin with or without irinotecan during HIPEC after cytoreductivesurgery for colorectal carcinomatosis. Ann. Surg. 2011;254, 294–301.
60. Yang XJ, Huang CQ, Suo T, Mei LJ, Yang GL, Cheng FL, Zhou YF, Xiong B, Yonemura Y, Li Y. Cytoreductive surgery and hyperthermic intraperitoneal chemotherapy improves survival of patients with peritoneal carcinomatosis from gastric cancer: final results of a phase III randomized clinical trial. Ann Surg Oncol. 2011;18:1575-81.
61. van Driel WJ, Koole SN, Sikorska K, Schagen van Leeuwen JH, Schreuder HWR, Hermans RHM, de Hingh IHJT, van der Velden J, Arts HJ, Massuger LFAG, Aalbers AGJ, Verwaal VJ, Kieffer JM, Van de Vijver KK, van Tinteren H, Aaronson NK, Sonke GS. Hyperthermic Intraperitoneal Chemotherapy in Ovarian Cancer. N Engl J Med. 2018;378:230-240.
62. Braam HJ, van Oudheusden TR, de Hingh IH, Nienhuijs SW, Boerma D, Wiezer MJ, et al. Patterns of recurrence following complete cytoreductive surgery and hyperthermic intraperitoneal chemotherapy in patients with peritoneal carcinomatosis of colorectal cancer. J Surg Oncol. 2014;109:841–7.
63. Passot G, Vaudoyer D, Cotte E, You B, Isaac C, Gilly FN, et al. Progression following neoadjuvant systemic chemotherapy may not be a contraindication to a curative approach for colorectal carcinomatosis. Ann Surg. 2012;256:125–9.
64. Passot G, You B, Boschetti G, Fontaine J, Isaac S, Decullier E, et al. Pathological response to neoadjuvant chemotherapy: a new prognosis tool for the curative management of peritoneal colorectal carcinomatosis. Ann Surg Oncol. 2014;21:2608–14.
65. Hompes D, Aalbers A, Boot H, van Velthuysen ML, Vogel W, Prevoo W, et al. A prospective pilot study to assess neoadjuvant chemotherapy for unresectable peritoneal carcinomatosis from colorectal cancer. Colorectal Dis. 2014;16:264–72.
66. Kwakman R, Schrama AM, van Olmen JP, Otten RH, de Lange-De Klerk ES, de Cuba EM, et al. Clinicopathological parameters in patients election for cytoreductve surgery and hyperthermic intraperitoneal chemotherapy for colorectal cancer metastases: a meta-analysis. Ann Surg. 2016;263:1102–11.
67. Eveno C, Passot G, Goéré D, Soyer P, Gayat E, Glehen O, et al. Bevacizumab doubles the early postoperative complication rate after cytoreductive surgery with hyperthermic intraperitoneal

chemotherapy (HIPEC) for peritoneal carcinomatosis of colorectal origin. Ann Surg Oncol. 2014;21:1792–800.

68. Devilee RA, Simkens GA, van Oudheusden TR, Rutten HJ, Creemers GJ, ten Tije AJ, et al. Increased survival of patients with synchronous colorectal peritoneal metastases receiving preoperative chemotherapy before cytoreductive surgery and hyperthermic intraperitoneal chemotherapy. Ann Surg Oncol. 2016;23:2841–8.
69. Ceelen W, van Nieuwenhove Y, Putte DV, Pattyn P. Neoadjuvant chemotherapy with bevacizumab may improve outcome after cytoreduction and hyperthermic intraperitoneal chemoperfusion (HIPEC) for colorectal carcinomatosis. Ann Surg Oncol. 2014;21:3023–8.
70. Kuijpers AM, Mehta AM, Boot H, van Leerdam ME, Hauptmann M, Aalbers AG, et al. Perioperative systemic chemotherapy in peritoneal carcinomatosis of lymph node positive colorectal cancer treated with cytoreductive surgery and hyperthermic intraperitoneal chemotherapy. Ann Oncol. 2014;25:864.
71. van Eden WJ, Kok NF, Józwiak K, Lahaye ML, Geets GL, van Leerdam ME, et al. Timing of systemic chemotherapy in patients with colorectal peritoneal carcinomatosis treated with cytoreductive surgery and hyperthermic intraperitoneal chemotherapy. Dis Colon Rectum. 2017;60:477–87.
72. Rovers KP, Bakkers C, van Erning FN, Burger JWA, Nienhuijs SW, Simkens GAAM, Creemers GM, Hemmer PHJ, Punt CJA, Lemmens VEPP, Tanis PJ, de Hingh IHJT. Adjuvant Systemic Chemotherapy vs Active Surveillance Following Up-front Resection of Isolated Synchronous Colorectal Peritoneal Metastases. JAMA Oncol. 2020;6:e202701.
73. Rovers KP, Simkens GA, Punt CJ, van Dieren S, Tanis PJ, de Hingh IH. Perioperative systemic therapy for resectable colorectal peritoneal metastases: sufficient evidence for its widespread use? A critical systematic review. Crit Rev Oncol Hematol. 2017;114:53–62.
74. Waite K, Youssef H.The Role of Neoadjuvant and Adjuvant Systemic Chemotherapy with Cytoreductive Surgery and Heated Intraperitoneal Chemotherapy for Colorectal Peritoneal Metastases: A Systematic Review. Ann Surg Oncol. 2017;24:705-720.
75. http://www.progettorol.it/ROL/lineeguida_LLGG.asp
76. https://www.aiom.it/linee-guida-aiom-2018-tumori-peritoneali-primitivi-e-secondari/
77. https://www.aiom.it/linee-guida-aiom-2018-tumori-del-colon/
78. Willaert W, van der Speeten K, Liberale G, Ceelen W. BEV-IP: perioperative chemotherapy with bevacizumab in patients undergoing cytoreduction and intraperitoneal chemoperfusion for colorectal carcinomatosis. BMC Cancer. 2015;15:980.
79. Leimkühler M, Hemmer PHJ, Reyners AKL, de Groot DJA, van Ginkel RJ, Been LB, de Bock GH, van Leeuwen BL. Neoadjuvant chemotherapy followed by cytoreductive surgery and hyperthermic intraperitoneal chemotherapy for colorectal cancer: a feasibility and safety study. World J Surg Oncol. 2019;17:14.
80. Rovers KP, Bakkers C, Simkens GAAM, et al. Perioperative systemic therapy and cytoreductive surgery with HIPEC versus upfront cytoreductive surgery with HIPEC alone for isolated resectable colorectal peritoneal metastases: protocol of a multicentre, open-label, parallel-group, phase II-III, randomised, superiority study (CAIRO6) BMC Cancer 2019 ;19:390.
81. https://recist.eortc.org/recist-1-1-2/
82. http://evs.nci.nih.gov/ftp1/CTCAE/CTCAE_4.03_2010-06-14_QuickReference_5x7.pdf.
83. Aaronson NK, Bullinger M, Ahmedzai S. A modular approach to quality-of-life assessment in cancer clinical trials. Recent results in cancer research Fortschritte der Krebsforschung 1988; 111:231-49.
84. Whistance RN, Conroy T, Chie W, Costantini A, et al. Clinical and psychometric validation of the EORTC QLQ-CR29 questionnaire module to assess health-related quality of life in patients with colorectal cancer. Eur J Cancer 2009, 45: 3017-26.
85. Jaquet P, Sugarbaker PH. Current methodologies for clinical assessment of patients with peritoneal carcinomatosis. J Exp Clin Cancer Res. 1996;15:49-58.
86. Fujii M, et al. A colorectal tumor organoid library demostrates progressive loss of niche factor requirements during tumorigenesis. Cell Stem Cell 2016;18:827-838.

87. Garnett MJ, et al. Systematic identification of genomic markers of drug sensitivity in cancer cells. Nature. 2012;483:570-5.van de Wetering M, et al. Cell. 2015;161:933-45.
88. van de Wetering M, et al. Prospective derivation of a living organoid biobank of colorectal cancer patients. Cell. 2015;161:933-45.
89. Candini O, et al. A Novel 3D In Vitro Platform for Pre-Clinical Investigations in Drug Testing, Gene Therapy, and Immuno-oncology. Sci Rep. 2019;9:7154.
90. Zheng, L. et al. Fumarate induces redox-dependent senescence by modifying glutathione metabolism. Nat. Commun. 2015; 6, 6001.
91. De Bortoli M, et al. Lipid accumulation in human breast cancer cells injured by iron depletors. J Exp Clin Cancer Res. 2018;37:75.
92. Elias D, Bonnay M, Puizillou J, Antou, S, Demrdijan S, El OA, et al. Heated intraoperative intraperitoneal oxaliplatin after complete resection of peritoneal carcinomatosis: pharmacokinetic and tissue distribution. Ann.Oncol. 200213, 267–272.
93. Witkamp A. Dose finding study of hyperthermic intraperitoneal chemotherapy with mitomycin C in patients with carcinosis of colorectal origin. Eur J Surg Oncol 1998;136:699–708.
94. Elias D, Matsuhisa T, Sideris L, Liberale G, Drurrad-Troalen L, Ranard B, et al. Heated intra-operative intraperitoneal oxaliplatin plus irinotecan aftercomplete resection of peritoneal carcinomatosis: pharmacokinetics, tissuedistribution and tolerance. Ann Oncol. 2004;15, 1558–1565.
95. Rossi CR, Foletto M, Mocellin S, et al. Hyperthermic intraoperative intraperitoneal chemotherapy with cisplatin and doxorubicin in patients who undergo cytoreductive surgery for peritoneal carcinomatosis and sarcomatosis: Phase I study. Cancer 2002;94:492–499.
96. Park BJ, Alexander HR, Libutti SK, et al. Treatment of primary peritoneal mesothelioma by continuous hyperthermic peritoneal perfusion (CHPP). Ann Surg Oncol 1999;6:582–590.
97. Kusamura S, Baratti D, Younan R, et al. Impact of cytoreductive surgery andhyperthermic intraperitoneal chemotherapy on systemic toxicity. Ann Surg Oncol. 2007;14:2550–2558.
98. Baratti D, Kusamura S, Mingrone E. Balestra MR, Laterza B. Deraco M. Identification of a Subgroup of Patients at Highest Risk for Complications After Surgical Cytoreduction and Hyperthermic Intraperitoneal Chemotherapy Ann Surg. 2012;256: 334–341
99. Steller MA, Egorin MJ, Trimble EL, et al.: A pilot phase I trial of continuous hyperthermic peritoneal perfusion with high-dose carboplatin as primary treatment of patients with small-volume residual ovarian cancer. Cancer Chemother Pharmacol 1999;43: 106–114.
100. Sardi A, Jimenez W, Nieroda C, Sittig M, Shankar S, Gushchin V. Melphalan: a promising agent in patients undergoing cytoreductive surgery and hyperthermic intraperitoneal chemotherapy Ann Surg Oncol 2014;21:908-14.
101. Fleming TR. One-sample multiple testing procedure for phase II clinical trials. Biometrics 1982;38: 143-151.
102. Jung SH, Lee TY, Kim KM, George S. Admissible two-stage designs for phase II cancer clinical trials, Statistics in Medicine 2004;23: 561-569.
103. Simon R. Controlled Clinical Trials 1989;10: 1-10.
104. Schulz KF, Altman DG, Moher D CONSORT 2010 statement: updated guidelines for reporting parallel group randomised trials. BMJ (Clinical research ed. 340: c332).
105. Kaplan EL, Meier P: Nonparametric estimation from incomplete observations. J Am Stat Soc 1958; 53:457–481.

Annex 1. World Health Organization (WHO) and Karnovski performance scores.

	ECOG score		Karnovski score
**0**	Asymptomatic (Fully active, able to carry on all predisease activities without restriction)	**100 %**	Normal; no complaints; no evidence of disease.
		**90 %**	Able to carry on normal activity; minor signs or symptoms of disease.
**1**	Symptomatic but completely ambulatory (Restricted in physically strenuous activity but ambulatory and able to carry out work of a light or sedentary nature. For example, light housework, office work)	**80 %**	Normal activity with effort; some signs or symptoms of disease.
		**70 %**	Cares for self; unable to carry on normal activity or to do active work.
**2**	Symptomatic, <50% in bed during the day (Ambulatory and capable of all self care but unable to carry out any work activities. Up and about more than 50% of waking hours)	**60 %**	Requires occasional assistance, but is able to care for most of their personal needs.
		**50 %**	Requires considerable assistance and frequent medical care.
**3**	Symptomatic, >50% in bed, but not bedbound (Capable of only limited self-care, confined to bed or chair 50% or more of waking hours)	**40 %**	Disabled; requires special care and assistance.
		**30 %**	Severely disabled; hospital admission is indicated although death not imminent.
**4**	Bedbound (Completely disabled. Cannot carry on any self-care. Totally confined to bed or chair)	**20 %**	Very sick; hospital admission necessary; active supportive treatment necessary.
		**10 %**	Moribund; fatal processes progressing rapidly.
**5**	Death	**0 %**	Dead.

Zubrod C, et al. Appraisal of methods for the study of chemotherapy in man: Comparative therapeutic trial of nitrogen mustard and thiophosphoramide. *Journal of Chronic Diseases*; 1960:11:7-33.

Karnofsky D, Burchenal J, The clinical evaluation of chemotherapeutic agents in cancer. In: MacLeod C, ed. Evaluation of Chemotherapeutic Agents. New York, NY: Columbia University Press; 1949:191–205.

Annex 2: American Society of Anestesiology (ASA) physical status classification system.

I **Patient is a completely healthy fit patient.**

(Healthy, non-smoking, no or minimal alcohol use)

II **Patient has mild systemic disease.**

(Mild diseases only without substantive functional limitations. Examples include (but not limited to): current smoker, social alcohol drinker, pregnancy, obesity (30 < BMI < 40), well-controlled DM/HTN, mild lung disease)

III **Patient has severe systemic disease that is not incapacitating.**

(Substantive functional limitations; One or more moderate to severe diseases. Examples include (but not limited to): poorly controlled DM or HTN, COPD, morbid obesity (BMI ≥40), active hepatitis, alcohol dependence or abuse, implanted pacemaker, moderate reduction of ejection fraction, ESRD undergoing regularly scheduled dialysis, premature infant PCA < 60 weeks, history (>3 months) of MI, CVA, TIA, or CAD/stents)

IV **Patient has incapacitating disease that is a constant threat to life.**

(Examples include (but not limited to): recent (< 3 months) MI, CVA, TIA, or CAD/stents, ongoing cardiac ischemia or severe valve dysfunction, severe reduction of ejection fraction, sepsis, DIC, ARD or ESRD not undergoing regularly scheduled dialysis)

V **A moribund patient who is not expected to live 24 hour with or without surgery.**

(Examples include (but not limited to): ruptured abdominal/thoracic aneurysm, massive trauma, intracranial bleed with mass effect, ischemic bowel in the face of significant cardiac pathology or multiple organ/system dysfunction)

Annex 3. EORTC QLQ-C30 quality of life questionnaire

ENGLISH

EORTC QLQ-C30 (version 3)

We are interested in some things about you and your health. Please answer all of the questions yourself by circling the number that best applies to you. There are no "right" or "wrong" answers. The information that you provide will remain strictly confidential.

Please fill in your initials: |__|__|__|__|
Your birthdate (Day, Month, Year): |__|__|__|__|__|__|__|__|
Today's date (Day, Month, Year): 31 |__|__|__|__|__|__|__|__|

	Not at All	A Little	Quite a Bit	Very Much
1. Do you have any trouble doing strenuous activities, like carrying a heavy shopping bag or a suitcase?	1	2	3	4
2. Do you have any trouble taking a <u>long</u> walk?	1	2	3	4
3. Do you have any trouble taking a <u>short</u> walk outside of the house?	1	2	3	4
4. Do you need to stay in bed or a chair during the day?	1	2	3	4
5. Do you need help with eating, dressing, washing yourself or using the toilet?	1	2	3	4

**During the past week:**	Not at All	A Little	Quite a Bit	Very Much
6. Were you limited in doing either your work or other daily activities?	1	2	3	4
7. Were you limited in pursuing your hobbies or other leisure time activities?	1	2	3	4
8. Were you short of breath?	1	2	3	4
9. Have you had pain?	1	2	3	4
10. Did you need to rest?	1	2	3	4
11. Have you had trouble sleeping?	1	2	3	4
12. Have you felt weak?	1	2	3	4
13. Have you lacked appetite?	1	2	3	4
14. Have you felt nauseated?	1	2	3	4
15. Have you vomited?	1	2	3	4
16. Have you been constipated?	1	2	3	4

<u>Please go on to the next page</u>

SPECIMEN

ENGLISH

During the past week:	Not at All	A Little	Quite a Bit	Very Much
17. Have you had diarrhea?	1	2	3	4
18. Were you tired?	1	2	3	4
19. Did pain interfere with your daily activities?	1	2	3	4
20. Have you had difficulty in concentrating on things, like reading a newspaper or watching television?	1	2	3	4
21. Did you feel tense?	1	2	3	4
22. Did you worry?	1	2	3	4
23. Did you feel irritable?	1	2	3	4
24. Did you feel depressed?	1	2	3	4
25. Have you had difficulty remembering things?	1	2	3	4
26. Has your physical condition or medical treatment interfered with your family life?	1	2	3	4
27. Has your physical condition or medical treatment interfered with your social activities?	1	2	3	4
28. Has your physical condition or medical treatment caused you financial difficulties?	1	2	3	4

For the following questions please circle the number between 1 and 7 that best applies to you

29. How would you rate your overall health during the past week?

1 2 3 4 5 6 7

Very poor Excellent

30. How would you rate your overall quality of life during the past week?

1 2 3 4 5 6 7

Very poor Excellent

SPECIMEN

© Copyright 1995 EORTC Quality of Life Group. All rights reserved. Version 3.0

Annex 4. EORTC QLQ-C29 quality of life questionnaire

ENGLISH

EORTC QLQ – CR29

Patients sometimes report that they have the following symptoms or problems. Please indicate the extent to which you have experienced these symptoms or problems during the past week. Please answer by circling the number that best applies to you.

During the past week:	Not at All	A Little	Quite a Bit	Very Much
31. Did you urinate frequently during the day?	1	2	3	4
32. Did you urinate frequently during the night?	1	2	3	4
33. Have you had any unintentional release (leakage) of urine?	1	2	3	4
34. Did you have pain when you urinated?	1	2	3	4
35. Did you have abdominal pain?	1	2	3	4
36. Did you have pain in your buttocks/anal area/rectum?	1	2	3	4
37. Did you have a bloated feeling in your abdomen?	1	2	3	4
38. Have you had blood in your stools?	1	2	3	4
39. Have you had mucus in your stools?	1	2	3	4
40. Did you have a dry mouth?	1	2	3	4
41. Have you lost hair as a result of your treatment?	1	2	3	4
42. Have you had problems with your sense of taste?	1	2	3	4

During the past week:	Not at All	A Little	Quite a Bit	Very Much
43. Were you worried about your health in the future?	1	2	3	4
44. Have you worried about your weight?	1	2	3	4
45. Have you felt physically less attractive as a result of your disease or treatment?	1	2	3	4
46. Have you been feeling less feminine/masculine as a result of your disease or treatment?	1	2	3	4
47. Have you been dissatisfied with your body?	1	2	3	4
48. Do you have a stoma bag (colostomy/ileostomy)? (please circle the correct answer)	Yes		No	

SPECIMEN

ENGLISH

Please go on to the next page

**During the past week:**	**Not at All**	**A Little**	**Quite a Bit**	**Very Much**
**Answer these questions ONLY IF YOU HAVE A STOMA BAG, if not please continue below:**				
49. Have you had unintentional release of gas/flatulence from your stoma bag?	1	2	3	4
50. Have you had leakage of stools from your stoma bag?	1	2	3	4
51. Have you had sore skin around your stoma?	1	2	3	4
52. Did frequent bag changes occur during the day?	1	2	3	4
53. Did frequent bag changes occur during the night?	1	2	3	4
54. Did you feel embarrassed because of your stoma?	1	2	3	4
55. Did you have problems caring for your stoma?	1	2	3	4
**Answer these questions ONLY IF YOU DO NOT HAVE A STOMA BAG:**				
49. Have you had unintentional release of gas/flatulence from your back passage?	1	2	3	4
50. Have you had leakage of stools from your back passage?	1	2	3	4
51. Have you had sore skin around your anal area?	1	2	3	4
52. Did frequent bowel movements occur during the day?	1	2	3	4
53. Did frequent bowel movements occur during the night?	1	2	3	4
54. Did you feel embarrassed because of your bowel movement?	1	2	3	4

**During the past 4 weeks:**	**Not at All**	**A Little**	**Quite a Bit**	**Very Much**
**For men only:**				
56. To what extent were you interested in sex?	1	2	3	4
57. Did you have difficulty getting or maintaining an erection?	1	2	3	4
**For women only:**				
58. To what extent were you interested in sex?	1	2	3	4
59. Did you have pain or discomfort during intercourse?	1	2	3	4

SPECIMEN

© QLQ-CR29 Copyright 2006 EORTC Quality of Life Group. All rights reserved. Version 2.1

Annex 5. EORTC QLQ-C30 quality of life questionnaire (Italian Translation)

EORTC QLQ-C30 (version 3)

Con questo questionario vorremmo sapere alcune cose su di Lei e sulla Sua salute. La preghiamo di rispondere a tutte le domande ponendo un cerchio attorno al numero che meglio corrisponde alla Sua risposta. Non esiste una risposta "giusta" o "sbagliata". Le Sue informazioni verranno tenute strettamente riservate.

Per favore scriva solo le iniziali del Suo nome: |____|

Data di nascita (g, m, a): |__|__||__|__||__|__|__|__|

La data di oggi (g, m, a): |__|__||__|__||__|__|__|__|

	No	Un po'	Parecchio	Moltissimo
1. Ha difficoltà nel fare lavori faticosi, come sollevare una borsa della spesa pesante o una valigia?	1	2	3	4
2. Ha difficoltà nel fare una lunga passeggiata?	1	2	3	4
3. Ha difficoltà nel fare una breve passeggiata fuori casa?	1	2	3	4
4. Ha bisogno di stare a letto o su una sedia durante il giorno?	1	2	3	4
5. Ha bisogno di aiuto per mangiare, vestirsi, lavarsi o andare in bagno?	1	2	3	4

**Durante gli ultimi sette giorni:**	**No**	**Un po'**	**Parecchio**	**Moltissimo**
6. Ha avuto limitazioni nel fare il Suo lavoroo i lavori di casa?	1	2	3	4
7. Ha avuto limitazioni nel praticare i Suoi passatempi hobby o altre attività di divertimento o svago?	1	2	3	4
8. Le è mancato il fiato?	1	2	3	4
9. Ha avuto dolore?	1	2	3	4
10. Ha avuto bisogno di riposo?	1	2	3	4
11. Ha avuto difficoltà a dormire?	1	2	3	4
12. Si è sentito debole?	1	2	3	4
13. Le è mancato l'appetito?	1	2	3	4
14. Ha avuto un senso di nausea?	1	2	3	4
15. Ha vomitato?	1	2	3	4

**Durante gli ultimi sette giorni:**	**No**	**Un po'**	**Parecchio**	**Moltissimo**
16. Ha avuto problemi di stitichezza?	1	2	3	4
17. Ha avuto problemi di diarrea?	1	2	3	4
18. Si è sentito stanco?	1	2	3	4
19. Il dolore ha interferito con le Sue attività quotidiane?	1	2	3	4
20. Ha avuto difficoltà a concentrarsi su cose come leggere un giornale o guardare la televisione?	1	2	3	4
21. Si è sentito teso?	1	2	3	4
22. Si è preoccupato?	1	2	3	4
23. Si è sentito irritabile?	1	2	3	4
24. Si è sentito depresso?	1	2	3	4
25. Ha avuto difficoltà a ricordare le cose?	1	2	3	4
26. Le Sue condizioni fisiche o il Suo trattamento medico hanno interferito con la Sua vita familiare?	1	2	3	4
27. Le Sue condizioni fisiche o il Suo trattamento medico hanno interferito con le Sue attività sociali?	1	2	3	4
28. Le Sue condizioni fisiche o il Suo trattamento medico Le hanno causato difficoltà finanziarie?	1	2	3	4

Per le seguenti domande ponga un cerchio intorno al numero da 1 a 7 che meglio corrisponde alla Sua risposta

29. Come valuterebbe in generale la Sua salute durante gli ultimi sette giorni?

Pessima 1 2 3 4 5 6 7 **Ottima**

30. Come valuterebbe in generale la Sua qualità di vita durante gli ultimi sette giorni?

Pessima 1 2 3 4 5 6 7 **Ottima**

Annex 6. EORTC QLQ-C29 quality of life questionnaire (Italian Translation)

EORTC QLQ – CR29

Talvolta i pazienti accusano i seguenti sintomi. La preghiamo di indicare il grado con cui ha provato questi sintomi durante gli ultimi sette giorni. Risponda tracciando un cerchio intorno al numero che meglio definisce la Sua situazione.

**Durante la settimana scorsa:**	**No**	**Un po'**	**Parecchio**	**Moltissimo**
31. Ha urinato spesso durante il giorno?	1	2	3	4
32. Ha urinato spesso durante la notte?	1	2	3	4
33. Ha subito una perdita involontaria di urina?	1	2	3	4
34. Ha provato dolore nell'urinare	1	2	3	4
35. Ha provato dolore addominale?	1	2	3	4
36. Ha provato dolori alle natiche o alla zona dell'ano o del retto?	1	2	3	4
37. Ha avvertito una sensazione di gonfiore all'addome?	1	2	3	4
38. Ha trovato sangue nelle feci?	1	2	3	4
39. Ha trovato muco nelle feci?	1	2	3	4
40. Ha provato secchezza alla bocca?	1	2	3	4
41. Ha perduto i capelli in seguito alla terapia?	1	2	3	4
42. Ha riscontrato problemi con il senso del gusto?	1	2	3	4

**Durante la settimana scorsa:**	**No**	**Un po'**	**Parecchio**	**Moltissimo**
43. Ha avuto preoccupazioni per la Sua salute futura?	1	2	3	4
44. Ha avuto preoccupazioni riguardo al peso?	1	2	3	4
45. Si è sentito/a fisicamente meno attraente in conseguenza della malattia o della terapia?	1	2	3	4
46. Si è sentito/a meno virile/femminile in conseguenza della malattia o della terapia?	1	2	3	4
47. Si è sentito/a insoddisfatto/a del Suo corpo?	1	2	3	4
48. Ha una sacca per stomia (colostomia/ileostomia)? (cerchiare la risposta corretta)	Sì	No		

Durante la settimena scorsa (Rispondere a queste domande **SOLO SE SI UTILIZZA UNA SACCA PER STOMIA**, altrimenti passare oltre):

	**No**	**Un po'**	**Parecchio**	**Moltissimo**
49. Ha riscontrato perdite involontarie di gas o flatulenze dalla sacca per stomia?	1	2	3	4
50. Ha riscontrato perdite di feci dalla sacca per stomia?	1	2	3	4
51. La pelle intorno allo stoma si è irritata?	1	2	3	4
52. Ha dovuto cambiare la sacca molto spesso durante il giorno?	1	2	3	4
53. Ha dovuto cambiare la sacca molto spesso durante la notte?	1	2	3	4
54. Ha provato imbarazzo riguardo allo stoma?	1	2	3	4
55. Ha avuto problemi nell'occuparsi della sacca per stomia?	1	2	3	4
Rispondere a queste domande **SOLO SE NON SI UTILIZZA UNA SACCA PER STOMIA:**				
49. Ha riscontrato perdite involontarie di gas o flatulenze dall'ano?	1	2	3	4
50. Ha riscontrato perdite di feci dall'ano?	1	2	3	4
51. La pelle intorno all'ano si è irritata?	1	2	3	4
52. È andato/a spesso di corpo durante il giorno?	1	2	3	4
53. È andato/a spesso di corpo durante la notte?	1	2	3	4
54. Ha provato imbarazzo nell'andare di corpo?	1	2	3	4

Durante le ultime 4 settimane:

	**No**	**Un po'**	**Parecchio**	**Moltissimo**
**Solo pero per gli uomini:**				
56. In che misura ha provato interesse per il sesso?	1	2	3	4
57. Ha incontrato difficoltà a ottenere o mantenere un'erezione?	1	2	3	4
**Solo per le donne:**				
58. In che misura ha provato interesse per il sesso?	1	2	3	4
59. Ha provato dolore o fastidio durante il rapporto sessuale?	1	2	3	4

ANNEX 7. Peritoneal cancer index (PCI)

Abdomino-pelvic regions

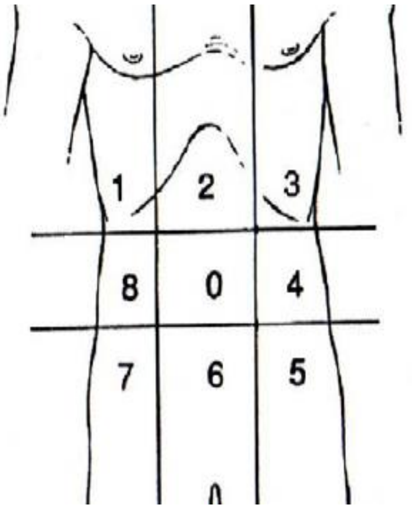

0 central
1 right upper
2 epigastrium
3 left upper
4 left flank
5 left lower
6 pelvis
7 right lower
8 right flank

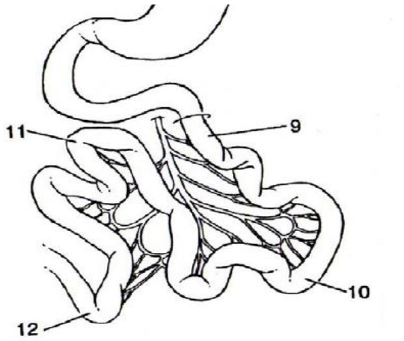

9 upper jejunum
10 lower jejunum
11 upper ileum
12 lower ileum

Peritoneal cancer index (PCI). The upper transverse line is located at the costal margin and the lower at the anterior superior iliac spine. Two sagittal lines divide the abdomen into three equal sectors. Nine regions are defined. The small bowel is divided into additional 4 regions. The anatomic structures involved in the 13 abdominopelvic regions are detailed. In each region, the greatest diameter of peritoneal tumor implants is rated according to the following semi-quantitative score:

- lesion size (LS)-0= no tumor;
- LS-1= ≤5mm;
- LS-2= >5mm and ≤50mm;
- LS-3= >50mm or confluent smaller tumor nodules.

The disease extent within all regions is indicated by a numerical score from 0 to 39, obtained by summing the LS-score of each region.
