# Supplementary material for: Tailoring HIPEC with Patient-Derived Organoids in Colorectal Peritoneal Metastases: Results from the First Stage of the Prospective Phase II OrganoHIPEC Clinical Trial (Clinicaltrials.gov NCT06057298)
Source: Cancers (Basel). 2026 Aug 21;18(16):2722. doi: 10.3390/cancers18162722 (PMC13511742; doi:10.3390/cancers18162722)
Supplement: Supplementary file 1 [file cancers-18-02722-s001.zip › Supplement S2.pdf]

Supplement S2

Patient ID	*PDO ID*	*PCI*	*First organoids (days)*	*First passage (days)*	*Assay readiness (days)*	*Passages before assay*
1	PM1	9	3	10	25	4
2	PM2	14	2	7	21	4
3	PM3	10	4	12	30	6
4	PM4	4	2	7	20	3
5	PM5	10	2	7	20	3
6	PM6	7	3	11	27	5
7	PM7	8	4	12	29	6
8	PM8	6	3	10	24	4
9	PM9	9	5	16	35	8
10	PM10	12	2	7	21	3
11	PM11	12	2	8	21	4
12	PM12	9	4	12	28	5
13	PM13	7	4	13	31	7
14	PM14	5	3	9	22	3
15	PM15	10	3	10	25	4
16	PM16	3	5	16	35	9
17	PM17	14	2	7	21	4
18	PM18	15	2	8	20	3
19	PM19	13	3	10	26	5
20	PM20	8	3	11	26	5
21	PM21	4	5	16	35	9
22	PM22	2	5	18	40	13
23	PM23	12	4	12	28	5
24	PM24	5	4	13	30	6
25	PM25	9	3	10	23	4
26	PM26	5	3	10	23	4
27	PM27	17	2	7	20	3
28	PM28	26	2	6	18	3
29	PM29	5	4	13	32	8
30	PM30	16	3	9	22	4
31	PM31	22	3	10	25	5

Table 1. Patient-by-patient PDO growth and expansion parameters, alongside the Peritoneal Cancer Index (PCI), in successfully established colorectal peritoneal metastasis-derived organoid lines. All time intervals are expressed in days from initial tissue processing. Time to first organoids was defined as the interval to the identification of visible organoids structures at the microscope. Time to first passage was defined as the interval to the first passage. Time to assay readiness was defined as the interval to the availability of a sufficient number of PDOs to perform the complete panel of in vitro HIPEC drug-sensitivity assays. Passages before assay was defined as to the total number of passages performed before the culture reached sufficient quantity for performing drug-sensitivity assays.
